# Spatial Au^+^ Mapping Reveals a Self‐Amplifying Anti‐Tumor Immunity Loop Driven by Au(I) Complexes

**DOI:** 10.1002/advs.77777

**Published:** 2026-10-11

**Authors:** Zhongren Xu, Zhiyue Fan, Lin Lv, Wei Lu, Zhenfan Wen, Wukun Liu

**Affiliations:** ^1^ Jiangsu Collaborative Innovation Center of Chinese Medicinal Resources Industrialization School of Medicine Nanjing University of Chinese Medicine Nanjing People's Republic of China

**Keywords:** bis‐*N*‐heterocyclic carbene Au(I) complex, chemoimmunotherapeutic, ferroptosis, immunogenic cell death

## Abstract

Although many metal complexes exhibit promising chemoimmunotherapeutic effects by inducing immunogenic cell death (ICD), the precise regulation of how these complexes interact with immune cells remains unclear. Herein, we introduced **Au9** as the first representative gold complex to directly associate with immune cells in the tumor immune microenvironment (TIME). Our study delineated the mechanism underlying **Au9**‐driven anti‐tumor response and “hot” TIME remodeling, anchored in Au^+^ spatial mapping. Of note, macrophages, dendritic cells (DCs), and tumor cells ranked as the top three in Au^+^ enrichment. Further investigation showed that **Au9** treatment stimulated the phagocytic activity of M1 macrophages, as confirmed by 3D scanning reconstructions. Importantly, **Au9** treatment triggered a self‐amplifying anti‐tumor immunity loop. This study pioneered critical insights into the mechanisms of TIME‐remodeling and laid the groundwork for the rational design of next‐generation gold complexes with enhanced chemoimmunotherapeutic efficacy.

## Graphical abstract

The first report of spatial Au^+^ mapping of a representative gold complex (**Au9**). Macrophages, DCs, and tumor cells ranked as the top three in Au^+^ enrichment after **Au9** treatment. Interestingly, **Au9** treatment could induce ferroptosis and suppress cellular cholesterol, further eliciting a self‐amplifying anti‐tumor immunity loop.

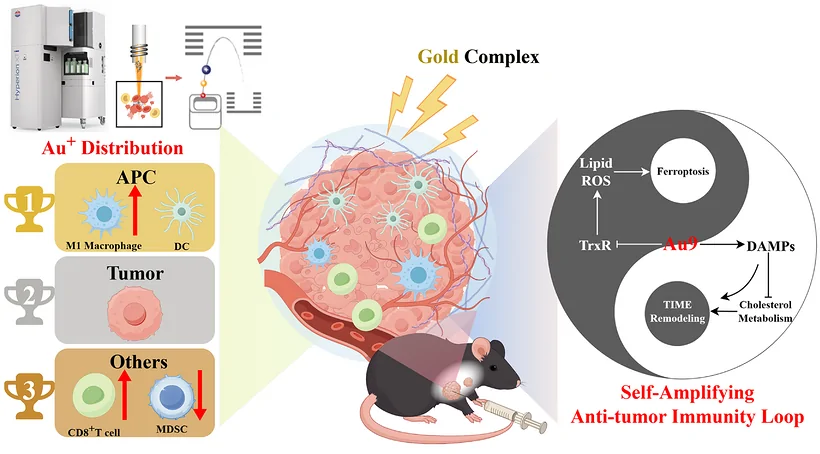

## 1 Introduction

Immunotherapy encompasses both experimental and standard clinical therapies and has emerged as a promising approach to stimulate the immune system to suppress tumors. Immune checkpoint inhibitors are widely used in the clinic [1, 2]. “Cold tumors” are characterized by loss of T cell infiltration or dysfunction, mainly due to immunosuppressive cells (regulatory T cells, myeloid‐derived suppressor cells, and M2 macrophages). Unfortunately, due to the “cold” TIME in most patients, therapeutic effects are minimal. A poor immunogenic tumor microenvironment, including decreased or dysfunctional immune cells and “cold” tumor signatures (non‐inflammatory gene transcriptional signatures), is associated with worse prognosis and a poor response to anti‐tumor immunotherapy [3]. Novel immunotherapies are still being actively explored, and some have emerged as promising applications, such as chemoimmunotherapy. Successful chemoimmunotherapy, which integrates chemotherapy with immunotherapy, can produce synergistic anti‐tumor effects [4]. To date, combining programmed cell death 1 (PD‐1) and programmed death ligand 1 (PD‐L1) inhibitors with platinum drugs has presented significant improvement (pathologic response and survival rates) in several tumors [5, 6]. These successful combined therapies underscore the importance of the continuous development of chemoimmunotherapeutic agents for clinical practice. The continuous development of platinum‐based and non‐platinum‐based anti‐tumor drugs is necessary for future chemoimmunotherapeutic applications.

Over the past decade, metal complexes have emerged as promising inducers of ICD, garnering significant interest in the field of chemoimmunotherapy. Numerous researchers have devoted efforts to developing ICD inducers, including platinum, gold, iridium, ruthenium, rhenium, copper, manganese, and gallium complexes [7, 8, 9, 10, 11, 12, 13, 14, 15, 16, 17, 18, 19, 20, 21, 22, 23, 24, 25, 26, 27, 28, 29, 30, 31, 32, 33]. Several ICD inducers were successfully developed, and the potential anti‐tumor mechanisms of these metal complexes were investigated. Previous studies have also shown that metal complexes, as promising ICD inducers, could enhance tumor‐derived antigens and promote CD8^+^ T cell responses. However, the details of the accurate regulation of TIME after metal complex treatment remain unclear, as do the distribution of metal ions and the immune cell response, especially in solid tumors. The delivery process of these special neoantigens needs to be investigated to inform the development of novel chemoimmunotherapeutic metal complexes in the future. However, to our knowledge, no study has yet directly investigated the role of metal complexes in relation to TIME. Addressing this knowledge gap would represent a significant advance for the field.

Recently, some gold complexes were discovered as potential anti‐tumor agents for chemotherapeutics or chemoimmunotherapeutics. Dinuclear orthometallated gold(I)‐gold(III) exhibited promising in vivo anti‐tumor effects in a dependent manner of ROS generation [34]. Sulfonamide‐derived dithiocarbamate gold(I) complexes induced apoptosis of colon cancer cells by inducing activation of Caspase 3 and ROS generation [35]. Two hydrophobic, gold‐containing anticancer agents varying in aromatic ligands with peptide nanofilaments were investigated [36]. Some effective microbiome‐modulating gold(I) complexes were discovered as an avenue to optimize cancer treatment outcomes and mitigate side effects [37]. 1,3‐diethyl‐4,5‐diphenyl‐4,5‐dihydro‐1H‐imidazol‐2‐ylidenes were discovered as novel ligands for *N*‐heterocyclic carbene (NHC)‐gold(I) complexes as selective anti‐tumor agents [38]. The gold monocarbene complex NHC‐gold(I)‐Cl could interact with human H ferritin (HuHf) as a potential anti‐tumor agent [39]. Some NHC‐gold(I) complexes were discovered and investigated as novel ferroptosis inducers for anti‐tumor therapy [40]. A new family of backbone‐functionalized NHC‐gold(I) complexes was designed and synthesized. The superior gold(I) complex exhibited more effective anti‐tumor effects than cisplatin and induced oxidative stress and ferroptosis [41]. So far, most gold(I) and gold (III) complexes have been identified as ROS generation inducers for chemotherapeutics [42]. The cyclometalated gold(III) complexes reprogrammed the TIME by targeting mitochondrial VDAC1 [43].

Our team has been engaged in developing gold complexes as chemotherapeutic agents for a long time. Most gold complexes have been proven to be promising ICD inducers. Notably, some gold complexes exhibited novel anti‐tumor mechanisms for sensitizing specific neoantigens to stimulate anti‐tumor immune response [23, 44, 45]. Although some chemotherapeutic gold complexes stimulated anti‐tumor immunity by inducing ferroptosis or ICD, the subsequent and precise analysis of TIME‐remodeling effects was not carried out. Currently, the accurate regulation of ferroptosis‐mediated ICD for TIME‐remodeling (distribution of immune cells) after gold complex treatment still remains unclear. Notably, we recently proposed a new theory, “golden immunology” for the design and development of next‐generation gold complexes as chemoimmunotherapeutic agents [46]. Continuously, this work was undertaken with the dual objectives of mapping the distribution of gold complexes across immune cell populations and deciphering the ferroptosis‐mediated mechanisms responsible for their anti‐tumor efficacy. Gold complexes are widely considered as effective thioredoxin reductase (TrxR) inhibitors and exhibit potential TIME‐remodeling activities. Besides, we previously found that the natural guaianolide sesquiterpene lactone micheliolide (MCL) induced ICD of hepatocellular carcinoma (HCC) by inhibiting TrxR activity via forming a covalent bond with the Sec498 residue of TrxR [47]. Inspired by our previous research, we have developed a series of dual pharmacophore TrxR inhibitors as representative gold complexes (Scheme 1). The superior gold complex, **Au9**, was selected for further investigation into TIME‐remodeling. The distribution of Au^+^ of **Au9** was mainly in macrophages, DCs, and tumor cells. Of note, there was no significant toxicity toward immune cells compared with the classic ICD inducer, oxaliplatin (OXA). Interestingly, a positive feedback loop was triggered by **Au9**, concurrently potentiating ferroptosis via cholesterol suppression and stimulating the TIME. To our knowledge, this study provides the first chemical‐biological evidence for the direct regulatory role of gold complexes in immune cells. By directly mapping their distribution, this study provides a molecular framework for TIME remodeling, informing the rational development of advanced gold‐based chemoimmunotherapeutic agents.

**SCHEME 1:**
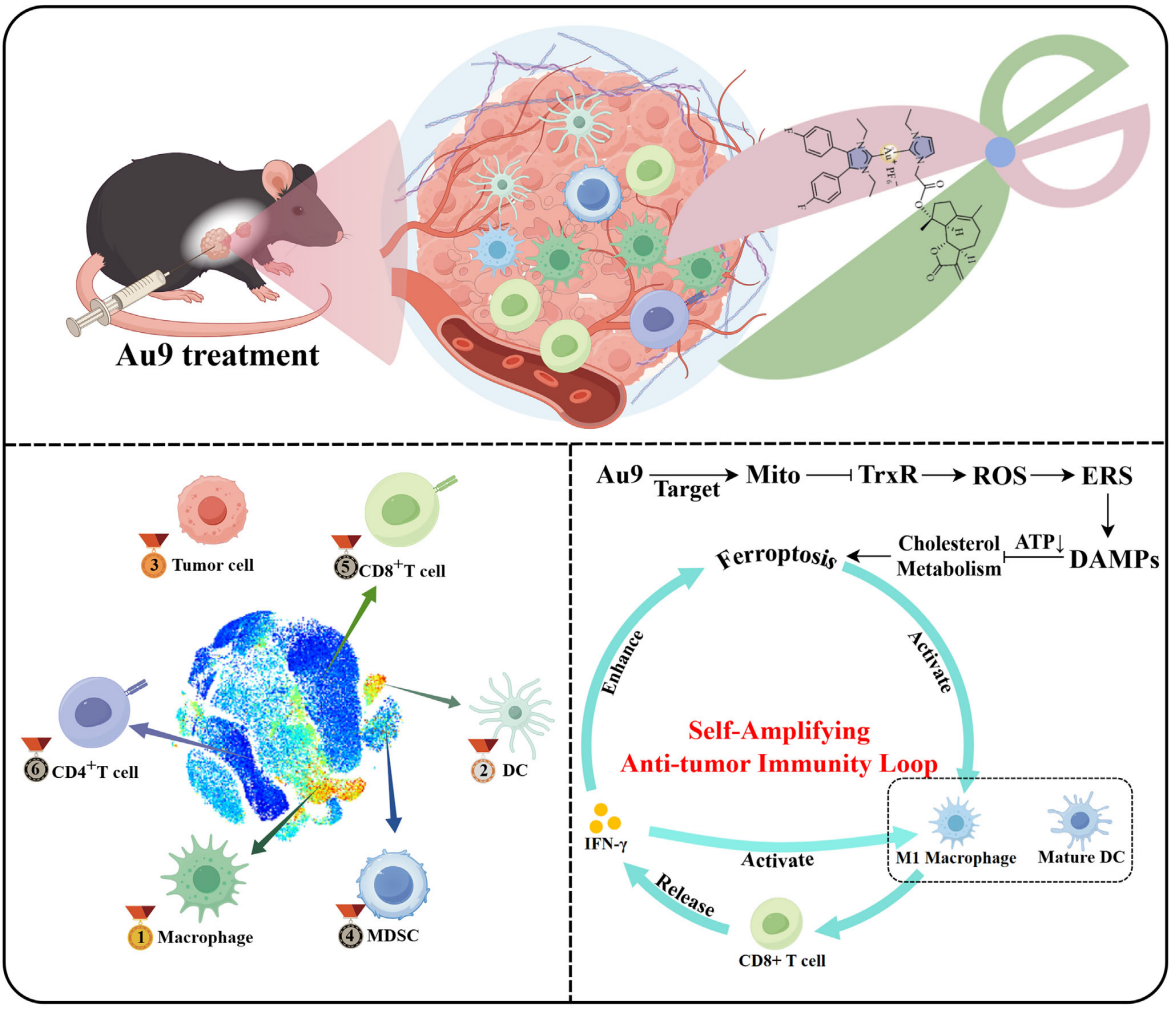
The representative gold complex **Au9** for direct association with immune cells by spatial Au^+^ mapping, and revealing a feedback loop of anti‐tumor immunity.

## 2 Results and Discussion

We draw upon the advantageous characteristics of NHC ligands established in our prior studies—namely their readily tunable molecular architecture, pronounced σ‐donating ability, weak π‐accepting nature, and propensity to form robust metal–carbon bonds [48, 49]. Notably, NHC can serve as an alternative to the triethylphosphine group, the active moiety in auranofin (AF). The NHC‐Au bond is one of the strongest carbene‐metal bonds. Gold‐NHC complexes have shown outstanding anti‐cancer activity [50]. MCL has been widely documented for its remarkable anti‐tumor and immunomodulatory activities, involving inhibition of TrxR and inhibition of macrophage ferroptosis [47, 51, 52]. Inspired by this, we propose a synergistic “dual‐blade scissors” design concept. The NHC‐gold(I) complexes and the MCL are covalently linked, resembling two independent blades of a pair of scissors. The NHC‐gold(I) blade efficiently inhibits TrxR and activates anti‐tumor immunity, whereas the MCL blade assists in TrxR inhibition and synergistically potentiates the immune response. When integrated into a single molecular entity, the two blades collaboratively “clip” the tumor defense machinery, leading to enhanced anti‐tumor activity, superior TrxR inhibition, and stronger immune activation. Compared to the classical TrxR inhibitor (AF), the “dual‐blade scissors” gold(I) complex (**Au9**) exhibited better TrxR inhibition and more effectively induced damage associated molecular patterns (DMAPs), which were intended to confer enhanced anti‐tumor activity and potential chemoimmunotherapy. Additionally, our group recently discovered novel gold(III) complexes as pyroptosis inducers by targeting mitochondrial DNA for anti‐tumor immunity [53]. In this study, we first introduced a representative series of NHC‐gold(I) complexes to investigate their engagement with immune cells in the TIME. Accordingly, a series of NHC‐gold(I) complexes incorporating MCL (**Au1**‐**Au6**) was synthesized. Following an initial activity assay, the ligand exhibiting the highest anti‐tumor potential was selected to prepare heteroleptic gold complexes (**Au7**‐**Au9**). Finally, the superior complex was functionalized with an aggregation‐induced emission (AIE)‐active tetraphenylethylene unit [54], yielding a fluorescent derivative designated as **Au10** (Figure 1A).

**FIGURE 1:**
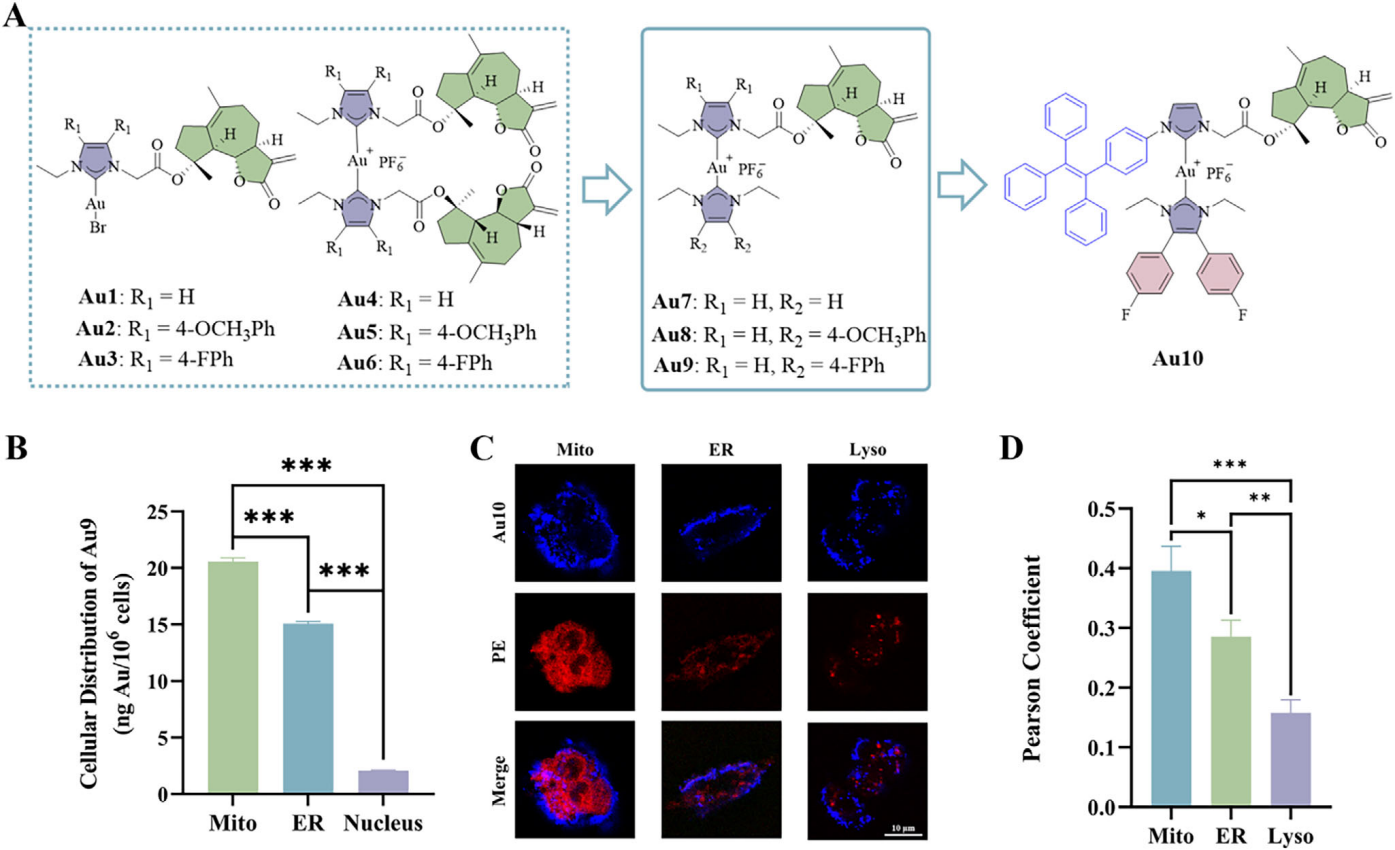
(A) The structures of ten gold complexes. (B) HepG2 cells were treated with **Au9** for 24 h. Cells were harvested, separated, and fractionated to extract Mito, Nucleus, and ER. The Au content in the samples was determined using inductively coupled plasma mass spectrometry (ICP‐MS). (C) The confocal laser scanning microscopy (CLSM) was used to analyze the co‐localization of **Au10** with Mito, ER, and Lyso in HepG2 cells. Scale bar: 10 µm. (D) Pearson correlation coefficient for co‐localization. *n* = 3; “^*^: *p* < 0.05”; “^**^:*p* < 0.01”; “^***^: *p* < 0.001”.

The synthesis of MCL was performed according to the previously reported procedure [55]. Subsequently, intermediate (**a**) was obtained via the esterification reaction of MCL with chloroacetyl chloride at room temperature. Refluxing intermediate (**a**) with NHCs (**b1‐b3**) afforded ligand (**c1‐c3**). The mono‐NHC‐gold(I) complexes incorporating MCL (**Au1**‐**Au3**) were synthesized via the silver carbene transfer method. Following this, the ligand substitution strategy was employed under alkaline conditions, wherein the free ligands (**c1‐c3**) reacted with **Au1**‐**Au3** to form cationic bis‐NHC‐gold(I) complexes incorporating MCL (**Au4**‐**Au6**). The heteroleptic bis‐NHC‐gold(I) complexes (**Au7**‐**Au10**) were synthesized following a protocol analogous to that used for **Au4**‐**Au6**. All chemical structures and synthetic routes of **Au1**‐**Au10** were depicted in Schemes S1‐S3. All gold(I) complexes were characterized by ^1^H nuclear magnetic resonance (NMR) spectroscopy, ^13^C NMR spectroscopy, electrospray ionization mass spectrometry (ESI‐MS), and high‐performance liquid chromatography (HPLC) (Figures S1–S38). In the ^1^H NMR spectra, the ligand exhibited a characteristic CH═N peak at δ = 10–11 ppm. However, this peak disappeared in both the mono‐NHC‐gold(I) complexes and bis‐NHC‐gold(I) complexes, indicating successful coordination of the gold center. In the ^1^ ^3^C NMR spectra, the chemical shift of Au‐C in the bis‐NHC‐gold(I) complexes showed a significant downfield shift compared to that of the mono‐NHC‐gold(I) complexes, with the chemical shift moving from approximately 175 to around 185 ppm. In addition, the purities of **Au1**‐**Au10** were greater than 95%.

For the above NHC‐gold(I) complexes, CCK‐8 assays were performed to investigate anti‐proliferation of complexes (Table S1). Most gold(I) complexes exhibited excellent cytotoxicity against HepG2, Hepa 1–6, and Huh7 cells within the range of IC_50_ 0.19–7.87 µM. The mono‐NHC‐gold(I) complexes (**Au1‐Au3**) exhibited slight anti‐proliferation activities. The cytotoxicity of bis‐NHC‐gold(I) complexes (**Au4‐Au6**) was further enhanced. Among them, **Au4** exhibited the most pronounced activity. Based on the findings above, we retained the ligand from **Au4** and synthesized the heteroleptic bis‐NHC‐gold(I) complexes (**Au7‐Au9**). The substituents also exerted a significant influence on the biological activity of the complexes. Specifically, in the mono‐NHC‐gold(I) complexes, the activity order was H > 4‐FPh > 4‐OCH_3_Ph. For the bis‐NHC‐gold(I) complexes, the order shifted to H > 4‐OCH_3_Ph > 4‐FPh. Interestingly, in the heteroleptic complexes, the order shifted to 4‐FPh > 4‐OCH_3_Ph > H. These complexes exhibited better anti‐proliferative activity than MCL and the positive controls. Among all the tested gold complexes, **Au9** showed the best inhibition against three tumor cell lines (HepG2: 0.20 ± 0.02 µM; Hepa 1–6: 0.41 ± 0.05 µM; Huh7: 0.23 ± 0.02 µM). Collectively, **Au9** was selected as a representative gold complex for further investigation of chemoimmunotherapy. The uptake distribution of the Au element in HepG2 cells was analyzed after exposure to **Au9** by the ICP‐MS process. As shown in Figure 1B, **Au9** mainly accumulated in the mitochondria (Mito) and endoplasmic reticulum (ER), and the least in the nucleus. To better investigate the cellular distribution and potential anti‐tumor mechanisms of **Au9**, the AIE‐active moiety was incorporated into **Au9**. The fluorescent complex of **Au9** was successfully synthesized, named **Au10**. Quantitative co‑localization was performed using commercial organelle probes for Mito, ER, and lysosomes (Lyso) (Figure 1C). The Pearson's correlation coefficients clearly revealed that **Au10** displayed the highest co‑localization with the mitochondrial marker (approximately 0.5), which was statistically significantly higher than that with ER (∼0.25) and Lyso (∼0.1) (^***^ *p* < 0.001) (Figure 1D). The colocalization results indicated that **Au10** mainly localized in the Mito and ER of HepG2 cells, which was almost consistent with the uptake of **Au9**. Collectively, these results indicated that **Au9** mainly targeted Mito and ER to suppress HepG2 cells, which guided subsequent investigation of anti‐tumor mechanisms.

To evaluate the stability of **Au9**, its ^1^H NMR spectra were recorded in the presence of mouse plasma, deuterium oxide (D_2_O), glutathione (GSH), *N*‐acetylcysteine (NAC), and L‐ascorbic acid (VC). As shown in Figures S39–S41, the ^1^H NMR spectra showed that **Au9** (5 mM) was stable in the presence of mouse plasma, D_2_O (10%), or NAC (5 mM) for at least 72 h, with no new peaks appearing. In the presence of GSH (5 mM), new peaks began to appear around *δ* = 3.11, 3.43, and 4.52 ppm after 12 h, accompanied by a gradual decrease in the intensity of the peak at *δ* = 3.35 ppm. However, this change remained minimal even after 72 h (Figure S42). After incubation with VC (5 mM) for 6 h, a new peak began to emerge around δ = 4.38 ppm, yet the variation remained slight after 72 h (Figure S43). These results indicated that **Au9** exhibited good stability under physiological conditions.

The TIME‐remodeling of gold complexes has been reported only sparingly. According to our previous studies and other research [56, 57, 58, 59], NHCs can effectively enhance the anti‐tumor activity of metal complexes, especially gold complexes. The promising ICD inducers of NHC‐gold complexes were first developed in 2020 [26]. Additionally, some bis‐NHC‐gold complexes have been identified as overcoming cisplatin resistance in ovarian cancers [60]. However, the key regulation of NHC‐gold complexes toward TIME has rarely been reported. Most research on novel NHC‐gold complexes has still focused on anti‐tumor or ICD‐inducing effects. The direct analysis of NHC‐gold complexes toward immune cells needs to be investigated, which would be critical for future chemoimmunotherapy of gold complexes in clinical application.

Encouraged by the promising anti‐tumor immune response observed with previous gold complexes, the specific immune cell signature following **Au9** treatment was worth investigating. OXA is a representative platinum chemotherapeutic drug, as a classical ICD inducer in in vivo experiments [61]. OXA treatment group was used as the positive control in tumor‐bearing models. The bilateral tumor‐bearing model was used to investigate the TIME‐remodeling of the superior **Au9**. As depicted in Figure 2A, the combination of anti‐PD‐L1 with **Au9** treatment was used to evaluate the potential of **Au9** as a novel chemotherapeutic agent. Both primary and distant tumors were suppressed after **Au9** treatment (Figure 2B,C). There was no significant change in body weight (Figure S44A) or organ damage across all groups (Figure S45). Interestingly, the combination group (**Au9** + anti‐PD‐L1) showed the greatest inhibition of distant tumor growth (incidence 60%), indicating promising anti‐tumor immune responses and potential TIME‐remodeling (Figure S44B).

**FIGURE 2:**
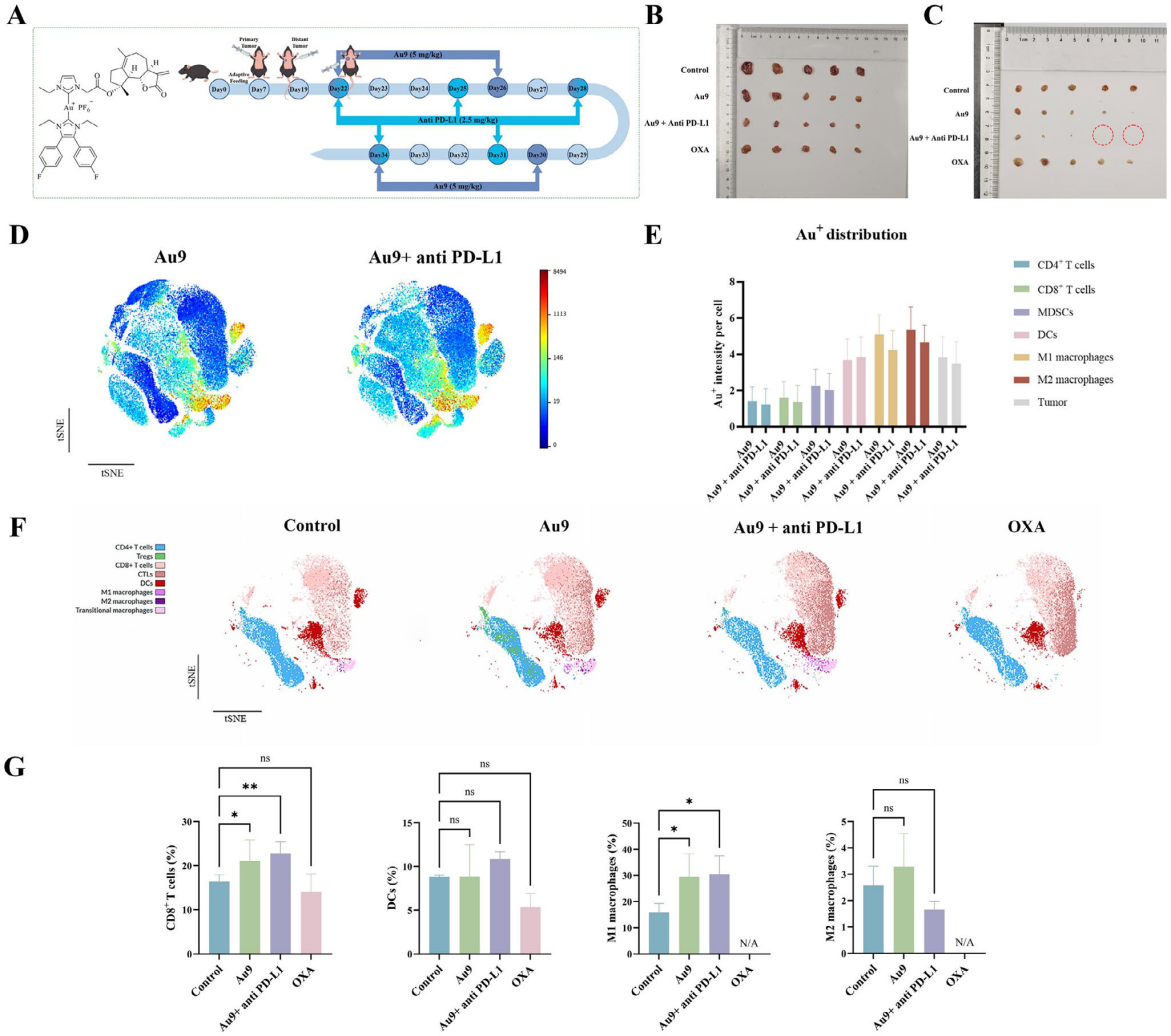
(A) Schematic illustrating the construction of a bilateral tumor model utilizing C57BL/6 mice. (B, C) The difference in size between primary and distant tumors following different treatment regimens. n = 5. (D, E) Tumor tissue samples from each group were subjected to CyTOF to determine the distribution and quantification of Au ions across different immune cell types. (F, G) Quantification of the relative abundance of immune cells in tumor tissues in four groups. *n* = 3; “ns: *p* > 0.05”;“^*^: *p* < 0.05”; “^**^:*p* < 0.01”. Compared with the control group.

To analyze the details of immune cells, a high‐accuracy CyTOF was used to assess the density and composition of tumor‐infiltrating immune cells. Single‐cell suspensions from mouse tumors were processed and stained with antibody panels. Importantly, this was the first time the Au^+^ distribution was directly detected by the metal label (Au‐sensor in CyTOF) (Figure S44C). As depicted in Figure 2D,E, the Au^+^ ions derived from the **Au9** were found to predominantly localize within antigen‐presenting cells (macrophages and DCs) as well as tumor cells. The Au^+^ uptake of both CD4^+^ and CD8^+^ T cells was almost half compared to the three kinds of cells above. It is well established that macrophages are the most abundant immune cells (30%–50%) in TIME, which can not only directly phagocytose and eliminate tumor cells, but also clear the tumor cell debris killed by T cells. We speculated that T cells killed the tumor cells, and macrophages may scavenge the tumor cell debris, because **Au9** was originally designed as a chemotherapy candidate for tumor treatment. Interestingly, there was no significant toxicity against macrophages after **Au9** treatment compared to OXA treatment (Figure 2F,G and Figure S44D), indicating the potential application of gold complexes in minimizing the toxic side effects of platinum drugs. Then, the results of the investigation into infiltrated immune cells indicated that there was substantial CD8^+^ T cell activation in both **Au9** treatment and combination groups, with more activated effector CD8^+^ T cells observed in the combination with anti‐PD‐L1. The percentage of M1/M2 macrophages was obviously increased in both **Au9** and the combination treatment. Notably, OXA treatment showed anti‐tumor activity, as evidenced by the photographs (Figure 2C). However, the number of infiltrating anti‐tumor immune cells was markedly reduced after intratumoral injection of OXA.

The tumor‐bearing model in C57BL/6 mice was further employed to investigate the potential chemoimmunotherapeutic effects of **Au9** treatment. As depicted in Figure S46A, Hepa 1–6 cells were injected into the left armpit of mice and randomly divided into four groups. The combination group (**Au9** and anti PD‐L1) exhibited the best inhibition of tumor growth when compared with the control group. Both **Au9** and OXA groups exhibited moderate tumor growth inhibition (Figure S46B,D). Also, there were no obvious changes in body weight in the three groups; a slight decrease in weight was observed in the OXA group, indicating the potential toxicity of OXA treatment (Figure S46C). The combination group also obviously reduced the PD‐L1 expression more than the other three groups. Interestingly, **Au9** treatment significantly decreased PD‐L1 expression in tumor tissues, indicating that **Au9** could directly suppress tumor growth and reduce T cell exhaustion, turning “cold” tumors into “hot” tumors (Figure S46E). As shown in Figure S47A, the TrxR expression was obviously decreased in the three groups. This observation in the **Au9** and combination groups was consistent with the in vitro cellular TrxR. We speculated that OXA treatment may degrade the TrxR protein. In addition, CRT exposure and decreased GPX4 were observed in the **Au9** alone and combination groups (Figure S47B,C).

Besides, the anti‐tumor immunity of the four groups was further investigated. The proportion of CD279^+^ cells in tumor tissues was significantly reduced in the **Au9** and combination groups, indicating that **Au9** treatment effectively reduced PD‐1 expression in immune cells. Importantly, the PD‐1 expression was higher after OXA treatment than in the other three groups (Figure S48A). The mature DCs (CD80^+^ and CD86^+^) were present in a higher proportion in the three treatment groups than in the control group (Figure S48B). The myeloid‐derived suppressor cells (MDSCs) (CD11b^+^ and Ly‐6C/Ly‐6G^+^) in the combination group were significantly reduced, but the proportion of MDSCs was increased after OXA treatment (Figure S48C). The augmented infiltration of CD8^+^ T cells (Figure S49A,B) and CD4^+^ T cells (Figure S49C,D) in tumor tissues from **Au9** and combination treatment was significantly higher than in the control group. In addition, **Au9** and combination treatment increased the proportion of M1/M2 in the tumor, while OXA treatment increased it slightly (Figure S49E,F). Collectively, these results strongly indicated that **Au9** treatment suppressed tumor growth and remodeled TIME under “hot” conditions more than OXA. The combination treatment also suggested **Au9** may be employed as a chemoimmunotherapeutic agent for further clinical application.

According to the in vivo investigation of immune cells, CD8^+^ T cells and M1 macrophages were identified as key effective anti‐tumor immune cells following **Au9** treatment. To further elucidate the M1/M2 macrophages and their phagocytic capabilities, a co‐culture procedure was employed (Figure 3A). To verify the stability of THP‐1‐derived macrophages, the photography of macrophages was observed, and CD11b was stained (Figure S50A,B). It's been proven that MCL could inhibit macrophage ferroptosis by competitively binding to the Arg483 site of KEAP1 [51]. The novel **Au9** may selectively suppress the M2 macrophages according to the observation of lactate dehydrogenase (LDH) analysis (Figure 3B), indicating the potential M2 macrophage targeting of the Au(I) group. The distribution of M1 macrophage infiltration into HepG2 cells was employed to evaluate the **Au9** effector as a potential chemotherapeutic agent. As shown in Figure 3C,D, CD86 (M1 polarization) macrophages were increased after the **Au9‐**treatment. Accurate analysis of the phagocytosis by M1 macrophages was employed through real‐time monitoring. **Au9**‐treated tumor cells (HepG2) were recognized, and accumulation of M1 macrophages at 3 h, and most HepG2 cells were phagocytosed from 6 h to the terminal 15 h (Figure 3E). The flow cytometric analysis results remained consistent (Figure S50C), and treatment with **Au9** augmented infiltration of macrophages to approximately1.5 times. Additionally, the pro‐inflammatory of macrophages was analyzed. The gene expression of three cytokines (TNF‐α, IL‐1β, and IL‐6) in macrophages in co‐culture systems was investigated. As shown in Figure 3F, the debris from **Au9** treatment could induce elevated gene expression as compared to the positive control (LPS + IFN‐γ), indicating that macrophages engulfing **Au9** treated debris exhibited increased secretion of pro‐inflammatory. The mono‐phagocytic process was recorded (Red‐M1 macrophages; Green‐HepG2 cells) (Figure 3G,H). The phagocytosis from 3D imaging was approximately the same as 2D imaging, and therefore we speculated that **Au9**‐treatment obviously induced M1 polarization and stimulated the phagocytosis of M1 macrophages. The above promising findings indicated that **Au9** not only served as a novel anti‐tumor agent but also possessed significant potential as a chemotherapeutic agent that could selectively induce M1 macrophages and stimulate phagocytic activities.

**FIGURE 3:**
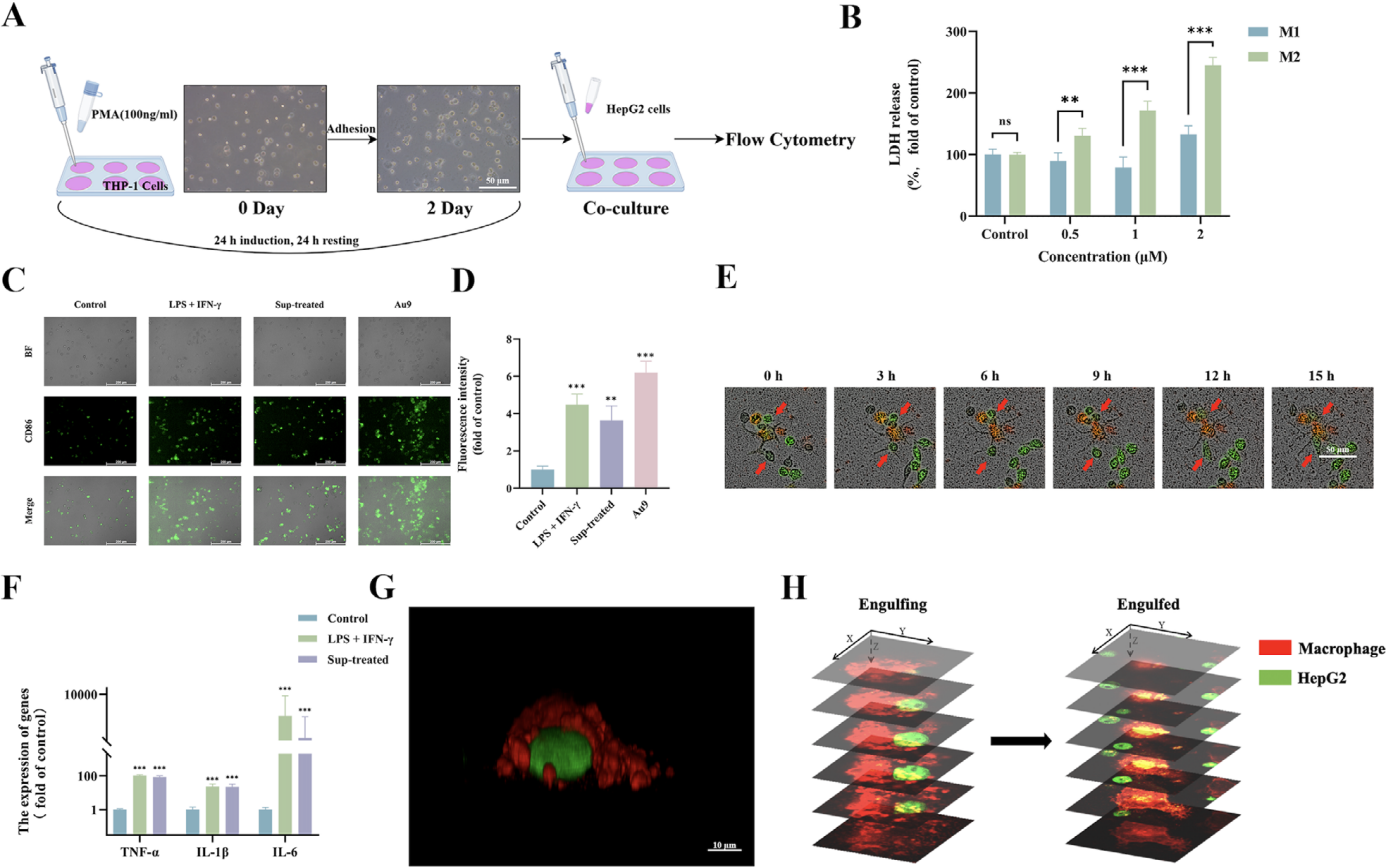
(A) THP‐1 cells were seeded in confocal microplates and cultured for 24 h. M0 macrophages were then cultured for 24 h in medium containing HepG2‐GFP drug‐treated supernatant. Following 20 min of fluorescent probe staining, digested and collected HepG2‐GFP cells were added to the confocal microplates at a 2:1 ratio for co‐culture for 12 h. Scale bar: 50 µm. (B) After treating with complexes at different concentrations for 24 h, LDH release in M1/M2 macrophages was analyzed. (C, D) Fluorescence microscope analysis of macrophages. Scale bar: 200 µm. (E) Long‐term dynamic live‐cell imaging and data analysis system for observing tumor phagocytosis. Scale bar: 50 µm. (F) The analysis of three genes in macrophages. (G, H) The dynamic process of co‐culture between M1 macrophages and HepG2 cells. Scale bar: 10 µm. *n* = 3; “ns: *p* > 0.05”; “^**^:*p* < 0.01”; “^***^: *p* < 0.001”. Compared with the control group.

It is widely recognized that gold complexes exhibit promising ICD‐inducing effects by targeting the TrxR in tumor cells. To investigate the precise role of TrxR in tumor progression and survival (especially in liver cancers), data mining of the latest clinical tumor databases was performed. According to the analysis of The Cancer Genome Atlas (TCGA), we observed that TrxR was highly expressed in liver cancer compared to normal liver tissues (Figure S51A). The observation of immunohistochemical (IHC) staining of normal liver tissues and tumor samples was consistent (Figure S51B). Importantly, TrxR was not only a clinical biomarker characteristic of liver cancer, but also negatively correlated with the survival prognosis of liver cancer patients (Figure S51C). We have previously demonstrated that the TrxR blocking elicited ROS‐mediated ER stress (ERS) stimulation in liver cancer cells, and this dual oxidative stress could further induce ICD effects [47]. Therefore, TrxR was overexpressed in liver cancer, which may be attributed to be potential target for TIME‐remodeling (“Cold” transforming to “Hot”). The investigation of isolated TrxR activities indicated that the superior **Au9** suppressed TrxR activities in a concentration‐dependent manner (Figure S51E). The IC_50_ value of **Au9** toward TrxR was 0.31 ± 0.04 µM (Figure S51F). As shown in Figure S52A, the expression of TrxR in HepG2 cells was decreased when the sample lysates were heated from 62°C–82°C. Obviously, the melt curve of the TrxR protein was reduced after **Au9** treatment, which may indicate that **Au9** broke the TrxR conformation. A specific TrxR‐green probe was used to analyze intracellular TrxR activities. Compared to the positive control (AF), decreased fluorescence intensity indicated intracellular TrxR activity inhibition in a concentration‐dependent manner (Figures S51D and S52B). But this inhibition did not affect TrxR protein expression levels (Figure S52C). Collectively, we identified a novel dual pharmacophore TrxR inhibitor that exhibited profound TrxR destabilization and may regulate TIME stimulation. Intracellular oxidative stress analysis indicated that obvious ROS generation was observed after **Au9** treatment (Figure S52D). After pretreatment with a selective inhibitor of eIF2α dephosphorylation (salubrinal), the dramatic anti‐proliferation of **Au9** was reversed (Figure S53A). Additionally, the results of the ERS‐related targets investigation indicated that **Au9** treatment increased the phosphorylated‐eIF2α and CHOP as compared to the control group, and pretreatment with salubrinal also reversed this stimulation (Figure S53B,C). This obvious observation demonstrated that **Au9** induced dual oxidative stress (ROS‐ERS). Dying tumor cells would transform into DAMPs when exposed to continual intracellular oxidative stress (especially ERS). We observed significant CRT exposure and HMGB1 release after **Au9** treatment of HepG2 cells, with levels more pronounced than those in the positive control AF group (Figure S53D).

According to the observation of Au^+^ spatial mapping, the regulation of **Au9** toward TIME was investigated. Therefore, the accurate anti‐tumor mechanisms of **Au9** needed to be analyzed, which was helpful in clarifying the anti‐tumor immunity of **Au9** treatment. The transcriptomic analysis was conducted to explore the anti‐tumor mechanisms of the **Au9**, to identify differentially expressed genes (DEGs) and altered biological pathways. KEGG pathway enrichment analysis revealed that cholesterol metabolism was among the most significantly enriched (Figure 4A). In addition, the ferroptosis and PD‐L1‐related pathways were also in the top‐ranked (Figure 4A). Potential genes of ferroptosis and cholesterol metabolism were analyzed by the transcriptome volcano plot   (Figure 4B,C). A small but notable subset of 9 genes of cholesterol metabolism (0.36%) exhibited siginifiacnt changed after **Au9** treatment, and  some targets (11 genes) were investigated by quantitative reverse transcription‐PCR (qRT‐PCR).  Among these targets, 8 genes were significantly changed in **Au9** group. Strikingly, a highly focused set of 6 genes of ferroptosis (0.24%) demonstrated differential expression. qRT‐PCR was also employed to investigate the accurate regulation of the **Au9**, 6 genes were also obviously changed after **Au9** treatment (Figure S54A,B).

**FIGURE 4:**
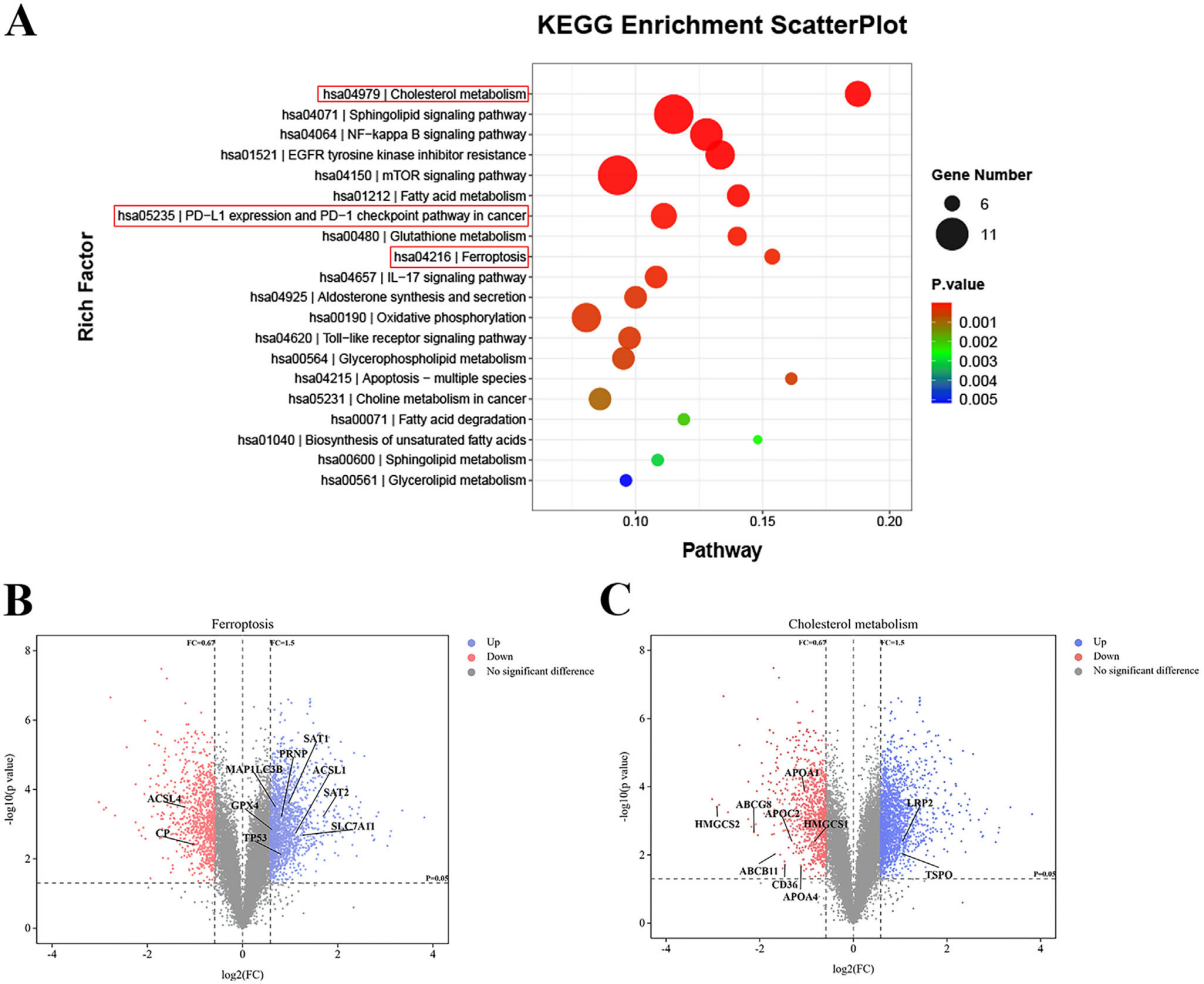
(A) KEGG enrichment pathway bubble chart derived from transcriptomic analysis. (B, C) Transcriptome volcano plot reveals differential genes in ferroptosis pathways and cholesterol metabolism.

Based on our discovery of the anti‐tumor mechanisms of the superior complex **Au9**, subsequent experiments investigated the effects of **Au9** treatment on ferroptosis and cholesterol metabolism. HepG2 cells were pretreated with different inhibitors (autophagy inhibitor: 3‐MA, pyroptosis inhibitor: disulfiram, and ferroptosis inhibitor: Fer‐1) before exposure to **Au9**. As shown in Figure S55A, a significant reversal with Fer‐1 was observed compared with the other two inhibitors, indicating that **Au9** could induce ferroptosis in HepG2 cells. The novel “ferroptosis suppression‐monitoring” integration probe (ferfluor‐1) was performed [62], and a vast generation of lipid peroxidation in the ER of HepG2 cells was observed in the **Au9** group compared to the positive control (RSL3) (Figure S55B). Accumulated Fe^2+^ was also observed after exposure to **Au9** (Figure S55C). In addition, we further performed CETSA to determine the interaction between **Au9** and the classic ferroptosis marker GPX4. A stable interaction with the GPX4 protein was observed at 74°C and 78°C after incubation with **Au9** (Figure S55D,E). IFN‐γ was generally considered a global regulatory factor in TIME. It's been shown that CD8^+^ T cell infiltration and IFN‐γ secretion can induce ferroptosis [63]. Interestingly, we also observed that pretreatment with IFN‐γ could sensitize **Au9** to ferroptosis induction, and this significant synergy could be reversed by the ferroptosis blocker (Fer‐1). IFN‐γ impaired cystine uptake and promoted ferroptosis in tumor cells via STAT1, downregulating SLC3A2 and SLC7A11 [64]. The STAT1 knockdown model of HepG2 cells was employed (Figure 5B and Figure S55F–I). As presented in Figure 5C, both **Au9** (2 µM) and **Au9** (4 µ) combined with IFN‐γ could synergistically suppress the proliferation of HepG2 cells, but there was an obvious reversal in the two groups when STAT1 was knocked down. We further analyzed the ferroptosis‐related markers (GPX4 and SLC7A11) in WT and si‐STAT1 HepG2 cells. The results indicated consistency with the above cell viability analysis. IFN‐γ could enhance the downregulation of the expression of GPX4 and SLC7A11, and these effects were reversed in the si‐STAT1 model (Figure S55J,K). Therefore, these promising results indicated that **Au9** elicited a classical positive anti‐tumor loop by inducing ferroptosis through the axis (IFN‐γ/STAT1/SLC7A11) (Figure 5A).

**FIGURE 5:**
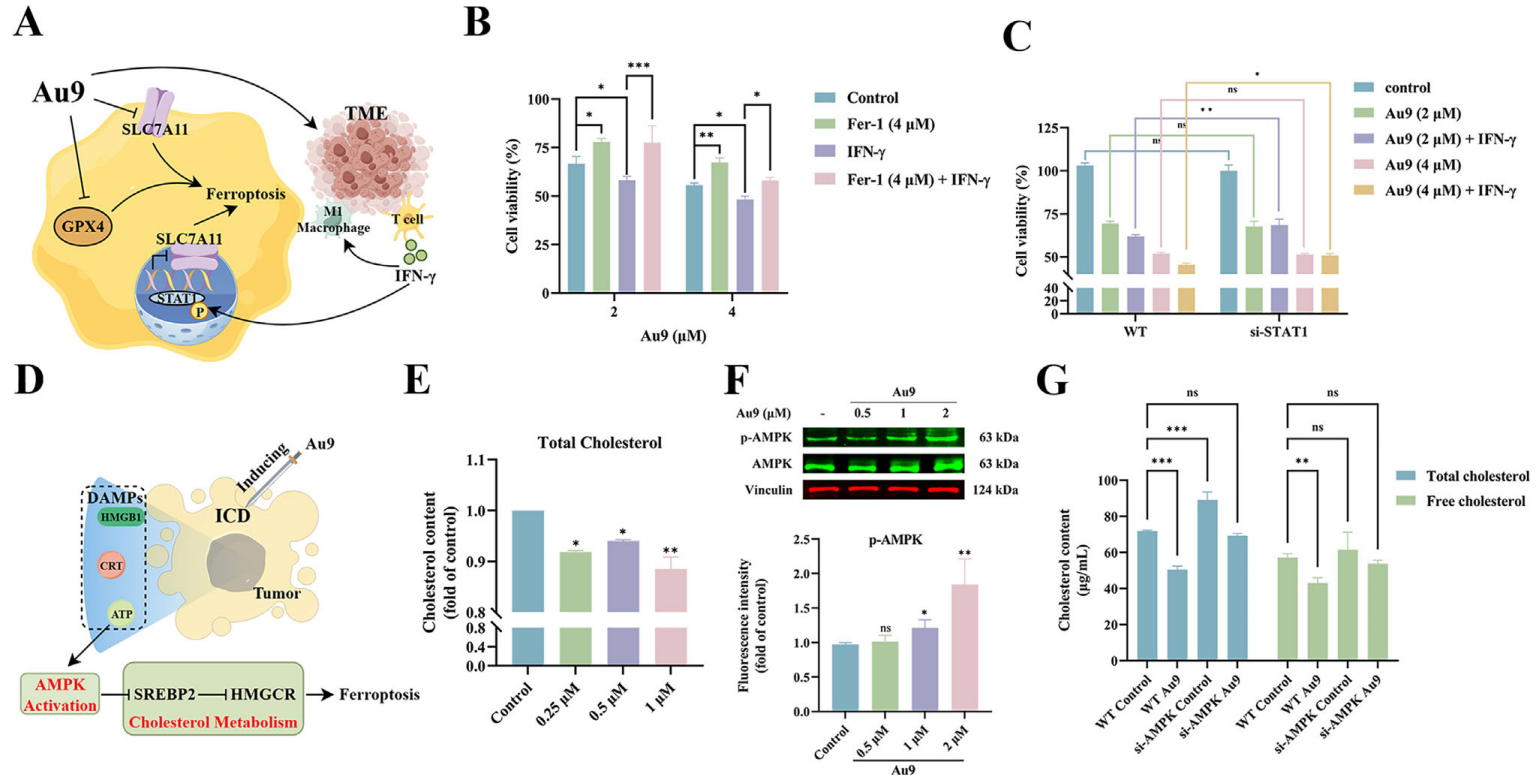
(A) The schematic mechanism of **Au9**‐induced ferroptosis. (B) Cell viability was investigated after **Au9** treatment under different processing conditions. (C) Cell viability was assessed after **Au9** treatment in STAT1 knockdown vs. wild‐type HepG2 cells. (D) The schematic mechanism of cholesterol depletion sensitizes ferroptosis induction by **Au9**. (E) Total cholesterol content in HepG2 cells after 24 h treatment with **Au9** at different concentrations. (F) The analysis of P‐AMPK/AMPK in HepG2 cells after exposure to **Au9** at different concentrations by fluorescent western blotting. (G) The investigation of intracellular total cholesterol and free cholesterol levels in AMPK knockdown and wild‐type HepG2 cells. *n* = 3; “ns: *p* > 0.05”; “^*^: *p* < 0.05”; “^**^:*p* < 0.01”; “^***^: *p* < 0.001”. Compared with the control group.

According to the KEGG pathway enrichment analysis, cholesterol metabolism was among the most significantly enriched pathways. Suppression of the total cholesterol level of HepG2 cells was observed following **Au9** in a dose‐dependent manner (Figure 5E). Notably, obvious DAMPs and ROS were observed in HepG2 cells following **Au9** treatment. Electron transfer reactions in Mito were associated with intracellular ROS production. The results of mitochondrial potential changes showed obvious green fluorescence (JC‐1 monomers) in the **Au9** group, indicating that **Au9** dissipated the mitochondrial membrane potential (Figure S56A). Previous research revealed that overloading intracellular iron accompanies the distribution of mitochondrial electron transport, which inhibits the ATP levels and activates AMPK pathways [65, 66]. AMPK, a key regulator, is activated when intracellular ATP levels decrease. More importantly, the activation of AMPK inhibits cellular cholesterol and fatty acid synthesis [67]. Following the observation that **Au9** induced ATP release (Figure S56B), we also found that **Au9** induced AMPK phosphorylation in a dose‐dependent manner (Figure 5F). Given the central role of AMPK in cholesterol metabolism, a si‐AMPK model of HepG2 cells was generated (Figure S56C,D). The total cholesterol level in HepG2 cells was significantly reduced following **Au9** treatment; this inhibition was completely prevented in AMPK knockdown (si‐AMPK) conditions. The changes in free cholesterol levels in both WT and si‐AMPK HepG2 cells were consistent (Figure 5G). It's noteworthy that while **Au9** could suppress the proliferation of HepG2 cells in WT conditions, the suppression of HepG2 cells by **Au9** could be reversed in si‐AMPK (Figure S56E). We also observed synergistic anti‐proliferation against HepG2 cells with **Au9** combined with the AMPK activator (A769662) (Figure S56F). Previous studies identified that activated AMPK induced the phosphorylation of HMGCR, while phosphorylated HMGCR prevented the increase in cholesterol levels [68, 69]. As shown in Figure S56G, the increased expression of phosphorylated HMGCR was detected after exposure to **Au9** in a dose‐dependent manner. SREBP2 is a key transcriptional regulator of cholesterol synthesis and selectively regulates cholesterologenic enzymes. The decrease in AMPK phosphorylation is accompanied by increased nuclear SREBPs accumulation, which leads to elevated cholesterol levels [70, 71]. Based on our previous findings, we observed increased phosphorylated AMPK after **Au9** treatment. The results of western blot indicated that both SREBP2 precursor and nuclear SREBP2 were significantly decreased following **Au9** in a dose‐dependent manner. Importantly, **Au9** combined with the AMPK activator (A769662) could synergistically promote SREBP2 degradation (Figure S56H). Therefore, **Au9** treatment not only induced a classic positive feedback loop between ferroptosis and anti‐tumor immunity, but also elicited a novel enhanced loop by inhibiting cholesterol synthesis to sensitize ferroptosis induction (Figure 5D).

## 3 Conclusion

Spotlight on anti‐tumor immunity is promising for discovering chemoimmunotherapeutic agents. However, the focus on tumor cells and ICD effects limits the development of metal complexes, especially gold complexes. Further research may focus on metal complexes toward TIME, especially on the chain reactions underlying their anti‐tumor activity (in simple terms, TIME remodeling). In this study, we discovered the novel dual pharmacophore TrxR inhibitor (**Au9**), which exhibited effective anti‐tumor activity and remodeled TIME for “hot” tumors. More importantly, this study reported the first systematic analysis of Au^+^ distribution in the whole TIME. Based on the results of CyTOF, we systematically identified that Au^+^ was mainly taken up by the macrophages, DCs, and tumor cells. The Au^+^ distribution in CD4^+^ and CD8^+^ T cells was half that in the three cell types above. The high concentration of Au^+^ in macrophages may result from M1 macrophages engulfing cancer cell debris or direct cellular uptake of **Au9**. Importantly, we also found that **Au9** treatment induced ferroptosis and stimulated anti‐tumor immunity, which formed a positive feedback loop of ferroptosis induction and durable immune response. Interestingly, **Au9**‐mediated cholesterol suppression could sensitize tumor cells to ferroptosis, providing sustained anti‐tumor immunity. Collectively, the enhanced feedback loop of anti‐tumor immunity was first identified after **Au9** treatment. The **Au9** exhibited potential as a promising chemoimmunotherapeutic agent for further tumor treatment. This study first reported a representative gold complex, **Au9**, and we progressively elucidated the accurate regulation of gold complexes toward anti‐tumor immune cells. This work represents just the beginning of exploring gold complexes as chemoimmunotherapeutic agents. We are excited to have taken the first step toward this goal, paving the way for the future development of gold complexes that target anti‐tumor immune cells.

## Author Contributions

**Zhongren Xu**: conceptualization, investigation, writing – original draft, writing – review and editing, visualization, methodology, validation, software, formal analysis, project administration, data curation. **Zhiyue Fan**: conceptualization, investigation, Writing – review and editing, visualization, validation, methodology, software, formal analysis, data curation. **Lin Lv**: conceptualization, methodology, software, data curation, writing – original draft, writing – review and editing, visualization, validation, investigation. **Wei Lu**: conceptualization, investigation, methodology, validation, software, writing – review and editing, data curation. **Zhenfan Wen**: conceptualization, writing – review and editing, visualization, validation, data curation, investigation, methodology. **Wukun Liu**: writing – review and editing, resources, supervision, data curation, software, formal analysis, project administration, visualization, validation, methodology, conceptualization, investigation, funding acquisition, writing – original draft

## Conflicts of Interest

The authors declare no conflicts of interest.

## Supporting information




**Supporting File**: advs77777‐sup‐0001‐SuppMat.pdf.

## Data Availability

Research data are not shared.
