# Supplementary material for: Spatial Au+ Mapping Reveals a Self‐Amplifying Anti‐Tumor Immunity Loop Driven by Au(I) Complexes
Source: Adv Sci (Weinh). 2026 Oct 11:e77777. Online ahead of print. doi: 10.1002/advs.77777 (PMC13654858; doi:10.1002/advs.77777)
Supplement: Supplementary file 1 [file ADVS-9999-e77777-s001.pdf]

Supporting Information

Spatial Au⁺ Mapping Reveals a Self-Amplifying Anti-tumor Immunity Loop Driven by Au(I) Complexes

Zhongren Xu[a, +], Zhiyue Fan[a, +], Lin Lv[a, +], Wei Lu[a], Zhenfan Wen[a], Wukun Liu[a,*]

Synthesis and Characterization S2

Biological Methods S9

^{1}H NMR and ^{13}C NMR Spectra S18

HPLC Chromatograms S32

Stability Analysis S36

Figure S39. The ^{1}H NMR spectra of **Au9** (5.0 mM) in DMSO-d_6 / plasma / D_2O = 19 : 4 : 1 over 72 h at 298 K. S36

Figure S40. The ^{1}H NMR spectra of **Au9** (5.0 mM) in DMSO-d_6 / D_2O = 9 : 1 over 72 h at 298 K. S36

Figure S41. The ^{1}H NMR spectra of **Au9** (5.0 mM) and NAC (5.0 mM) in DMSO-d_6 / Acetonitrile-d_3 = 1 : 1 over 72 h at 298 K.. .... S37

Figure S42. The ^{1}H NMR spectra of **Au9** (5.0 mM) and GSH (5.0 mM) in DMSO-d_6 / D_2O = 9 : 1 over 72 h at 298 K. S37

Figure S43. The ^{1}H NMR spectra of **Au9** (5.0 mM) and VC (5.0 mM) in DMSO-d_6 / D_2O = 9 : 1 over 72 h at 298 K. S38

Table S1. The anti-proliferation of complexes against three tumor cells (72 h). S38

Figure S44. Animal experiments: body weight, tumor volume, and mass cytometry results S39

Figure S45. The five organs were analyzed after four groups of treatments by HE staining S40

Figure S46. Evaluation of tumor volume, body weight changes, and PD-L1 expression in C57BL/6 mouse tumor models across treatment groups. S40

Figure S47. Immunofluorescence staining of TrxR/CRT/GPX4 protein expression S41

Figure S48. Flow cytometry detection of intratumoral immune-related indicators (PD-1, DCs, MDSCs). S42

Figure S49. Immunofluorescence staining of immunomarker expression within tumors across different treatment groups. S43

Figure S50. Differentiation of THP-1 cells into macrophages and tumor/macrophage co-culture S44

Figure S51. Evaluation of TXNRD differential expression, protein immunohistochemistry,

survival prognostic association, and intracellular TrxR activity in cancers and liver tissues. S44
Figure S52. Detection of TrxR and ROS levels. S45
Figure S53. Immunofluorescence detection of ERS markers (CHOP/p-eIF2α) and immunogenic cell death indicators (CRT/HMGB1) S46
Figure S54. Transcriptomic analysis of HepG2 cells after **Au9** treatment. S47
Figure S55. The investigation of mechanisms of ferroptosis of HepG2 cells. S48
Figure S56. Detection of mitochondrial function, AMPK regulation, and expression of downstream cholesterol metabolism-related proteins. S49
References S50

Synthesis and Characterization

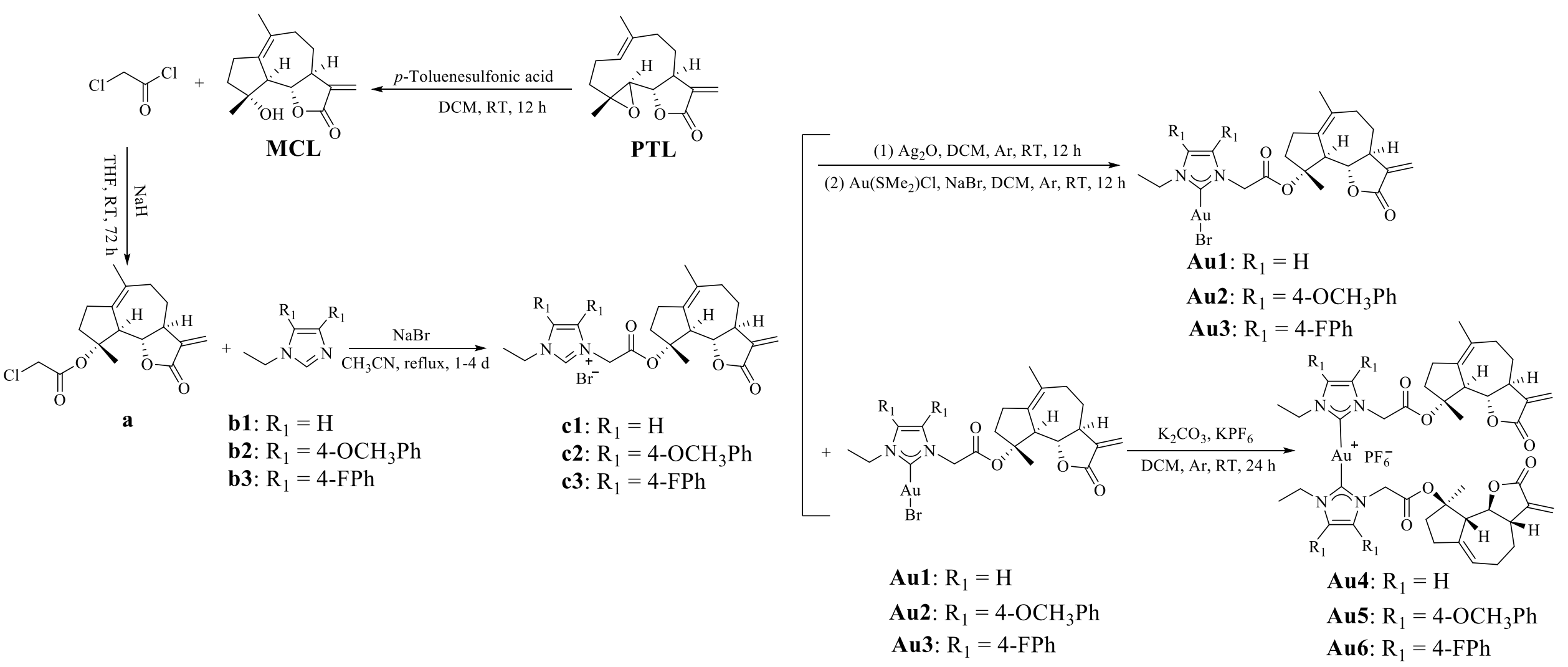

Scheme S1. Syntheses of **Au1**-**Au6**.

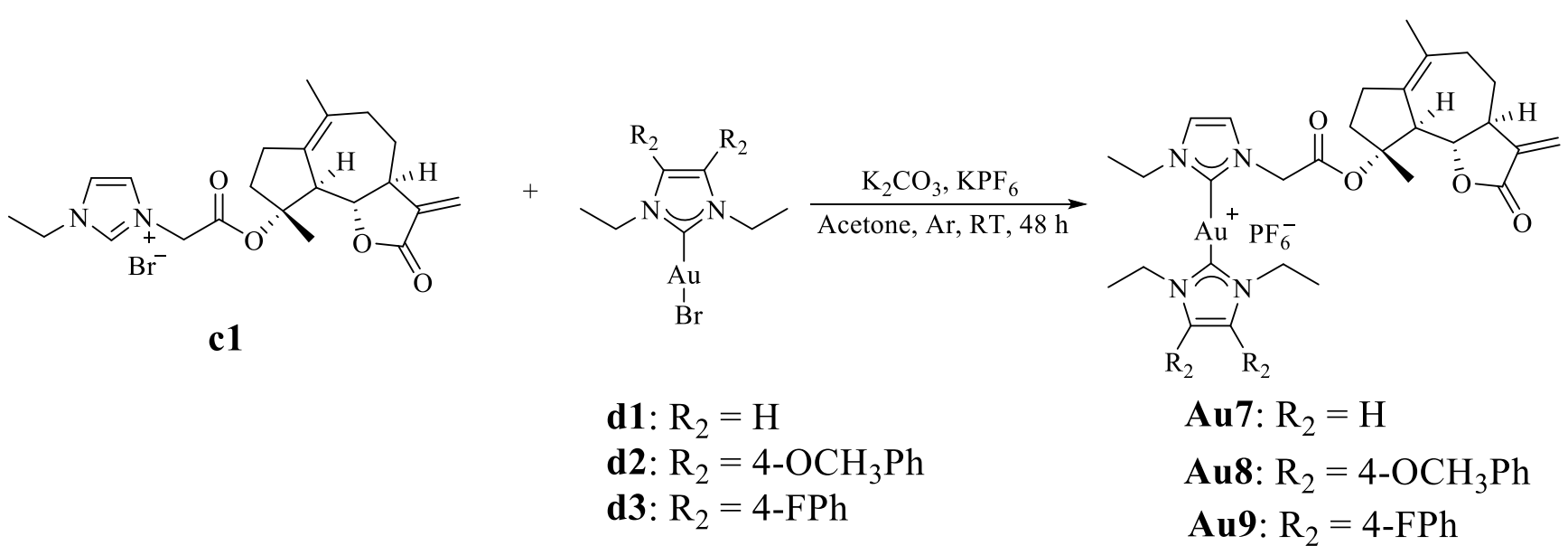

Scheme S2. Syntheses of **Au7**-**Au9**.

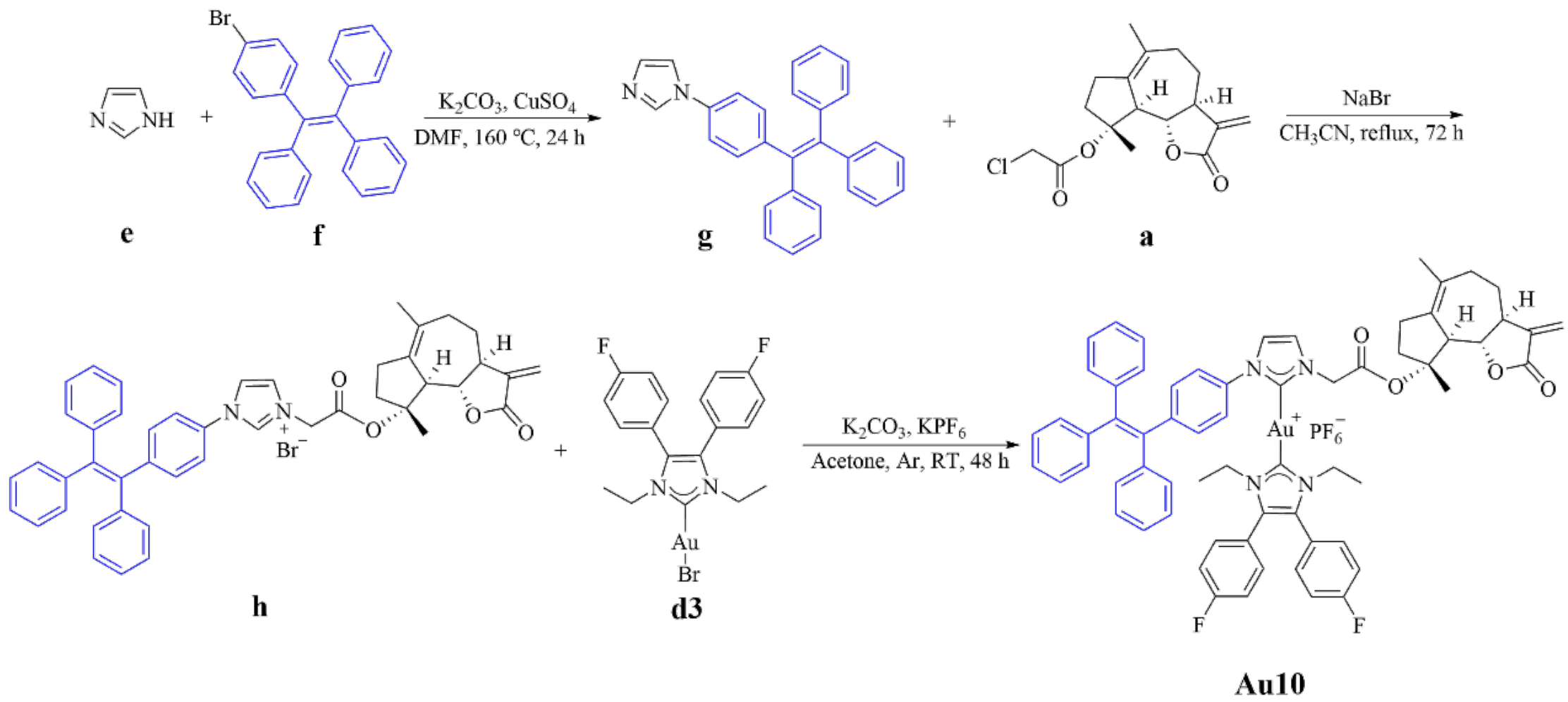

Scheme S3. Syntheses of **Au10**.

Synthesis of a

The synthesis of micheliolide (MCL) was carried out according to previously reported literature procedure [1]. MCL (1.0 equiv) was dissolved in anhydrous tetrahydrofuran (THF) under ice-bath cooling. Sodium hydride (NaH, 50.0 equiv) was added, followed by chloroacetyl chloride (30.0 equiv). The reaction mixture was then stirred at room temperature for 3 days. After completion, the mixture was extracted with ethyl acetate (EA). The organic layer was sequentially washed with saturated aqueous citric acid solution, saturated sodium bicarbonate ($NaHCO_3$) solution, and saturated sodium chloride (NaCl) solution. The crude product was purified by column chromatography on alumina (Al_2O_3) using a mixture of petroleum ether (PE) and EA as eluent to afford intermediate **a**.

a: Yellow oily substance; Yield 36.7%; ^{1}H NMR (500 MHz, Chloroform-*d*) δ 6.20 (d, J = 3.4 Hz, 1H, C=CH_2), 5.47 (d, J = 3.1 Hz, 1H, C=CH_2), 4.06 (d, J = 7.7 Hz, 2H, CH_2Cl), 3.78 (t, J = 10.1 Hz, 1H, OCH), 3.15 (d, J = 10.5 Hz, 1H), 2.72 – 2.64 (m, 1H), 2.52 – 2.41 (m, 2H), 2.27 (dd, J = 7.7, 4.1 Hz, 3H), 2.13 – 2.06 (m, 1H), 1.99 (dt, J = 20.9, 10.1 Hz, 2H), 1.72 (s, 3H, CH_3), 1.58 (s, 3H, CH_3).

Syntheses of c1-c3

Compound **b1** is commercially available and was used without further purification. The synthesis of **b2-b3** followed a previously reported procedure [2]. Intermediate **a** (1.0 equiv) and **b1**-**b3** (1.2 equiv) were dissolved in anhydrous acetonitrile (CH_3CN), followed by the addition of sodium bromide (NaBr, 5.0 equiv). The mixture was stirred under reflux for 1-4 d. After completion, the solid was removed by filtration, and the filtrate was purified by silica gel column chromatography using a mixture of dichloromethane (DCM) and methanol (MeOH) as eluent to afford ligands **c1**-**c3**.

c1: Yellow solid; Yield 97.5%; ^{1}H NMR (500 MHz, Chloroform-*d*) δ 10.51 (s, 1H, CH=N), 7.49 (s, 1H, CH=CH), 7.29 (s, 1H, CH=CH), 6.17 (d, *J* = 3.3 Hz, 1H, C=CH_2), 5.49 (d, *J* = 3.1 Hz, 1H, C=CH_2), 5.42 (d, *J* = 17.4 Hz, 1H), 5.29 (d, *J* = 17.4 Hz, 1H), 4.45 – 4.29 (m, 2H), 3.77 (t, *J* = 10.1 Hz, 1H, OCH), 3.22 (d, *J* = 10.4 Hz, 1H), 2.74 – 2.66 (m, 1H), 2.47 (dd, *J* = 16.1, 8.7 Hz, 1H), 2.41 (dd, *J* = 11.6, 7.2 Hz, 1H), 2.36 – 2.18 (m, 4H), 2.16 – 2.06 (m, 2H), 1.69 (s, 3H, CH_3), 1.60 (t, *J* = 7.3 Hz, 3H, CH_3), 1.52 (s, 3H, CH_3). ^{13}C NMR (126 MHz, Chloroform-*d*) δ 164.39, 139.10, 138.29, 132.34, 128.87, 123.65, 121.15, 119.57, 91.92, 83.18, 56.20, 51.23, 49.57, 45.63, 36.12, 35.03, 30.33, 29.82, 25.93, 24.27, 18.94, 15.45.

c2: White solid; Yield 93.4%; ^{1}H NMR (500 MHz, Chloroform-*d*) δ 10.62 (s, 1H, CH=N), 7.24 – 7.17 (m, 2H, ArH), 7.15 – 7.08 (m, 2H, ArH), 6.90 (d, *J* = 8.0 Hz, 4H, ArH), 6.14 (d, *J* = 3.3 Hz, 1H, C=CH_2), 5.44 (d, *J* = 3.1 Hz, 1H, C=CH_2), 5.27 (d, *J* = 17.1 Hz, 1H), 5.15 (d, *J* = 17.3 Hz, 1H), 4.24 – 4.15 (m, 2H), 3.79 (d, *J* = 2.9 Hz, 6H, OCH_3), 3.68 (t, *J* = 10.2 Hz, 1H, OCH), 3.17 (d, *J* = 9.9 Hz, 1H), 2.67 (m, 1H), 2.49 – 2.35 (m, 2H), 2.33 – 2.03 (m, 6H), 1.88 (s, 3H, CH_3), 1.46 (t, *J* = 7.3 Hz, 3H, CH_3), 1.43 (s, 3H, CH_3). ^{13}C NMR (126 MHz, Chloroform-*d*) δ 170.03, 165.12, 161.22, 161.06, 139.39, 137.64, 132.29, 132.11, 132.03, 131.19, 129.61, 119.02, 117.05, 116.31, 115.00, 114.82, 91.94, 82.88, 56.50, 55.48, 55.46, 49.99, 49.23, 43.51, 36.36, 34.93, 30.38, 25.99, 24.23, 18.82, 15.56.

c3: White solid; Yield 91.8%; ^{1}H NMR (500 MHz, Chloroform-*d*) δ 10.77 (s, 1H, CH=N), 7.35 (dd, *J* = 8.5, 5.1 Hz, 2H, ArH), 7.29 – 7.22 (m, 2H, ArH), 7.11 (t, *J* = 8.3 Hz, 4H, ArH), 6.15 (d, *J* = 3.3 Hz, 1H, C=CH_2), 5.46 (d, *J* = 3.0 Hz, 1H, C=CH_2), 5.23 (q, *J* = 17.2 Hz, 2H), 4.21 (q, *J* = 7.3 Hz, 2H), 3.69 (t, *J* = 10.1 Hz, 1H, OCH), 3.16 (d, *J* = 10.2 Hz, 1H), 2.67 (t, *J* = 7.3 Hz, 1H), 2.49 – 2.34 (m, 2H), 2.32 – 2.12 (m, 4H), 2.04 (q, *J* = 12.1 Hz, 2H), 1.68 (s, 3H, CH_3), 1.48 (t, *J* = 7.3 Hz, 3H, CH_3), 1.39 (s, 3H, CH_3). ^{13}C NMR (126 MHz, Chloroform-*d*) δ 170.07, 164.58, 164.02 (d, *J* = 253.3 Hz), 163.89 (d, *J* = 253.3 Hz), 139.19, 138.22, 132.93 (t, *J* = 7.6 Hz), 132.36, 131.77, 130.88, 129.16, 120.87 (d, *J* = 3.8 Hz), 120.23 (d, *J* = 2.5 Hz), 119.29, 116.90 (t, *J* = 23.9 Hz), 91.94, 82.90, 56.51, 49.77, 49.50, 43.77, 36.24, 34.95, 31.53, 30.36, 29.79, 25.96, 24.25, 18.63, 15.47.

Synthesis of h

Intermediate **g** was prepared following the previous procedure [3]. Intermediate **a** (1.0 equiv) and **g** (1.2 equiv) were dissolved in CH_3CN, then NaBr (5.0 equiv) was added. After stirring and reflux for 72 h, the solid was removed by filtration, and the filtrate was purified by silica gel column chromatography using a mixture of DCM and MeOH as eluent to afford ligand **h**.

h：Brown solid; Yield 63.5%; ^{1}H NMR (500 MHz, Chloroform-*d*) δ 10.63 (s, 1H, N-CH=N), 7.66 (s, 1H, ArH), 7.48 (s, 1H, ArH), 7.42 (d, *J* = 7.9 Hz, 2H, ArH), 7.23 (d, *J* = 7.4 Hz, 2H, ArH), 7.17 – 7.08 (m, 10H, ArH), 7.05 – 6.97 (m, 8H, ArH), 5.78 (s, 2H, $C=CH_2$), 4.28 (t, *J* = 6.1 Hz, 2H, NCH_2), 3.58 (s, 1H, OCH), 3.45 (t, *J* = 6.2 Hz, 2H), 2.28 (s, 6H), 1.98 (s, 2H), 1.88 (s, 3H).

Syntheses of Au1-Au3

Ligands **c1**-**c3** (1.0 equiv) were dissolved in dry DCM. Silver oxide (Ag_2O, 0.6 equiv) was then added under an argon (Ar) atmosphere, and the reaction mixture was stirred at room temperature overnight in the darkness. Subsequently, dimethylsulfide gold chloride ($Au(SMe_2)Cl$, 1.0 equiv) and NaBr (10.0 equiv) were added, and stirring was continued under the same conditions for an additional 12 h. The mixture was filtered through celite, and the filtrate was purified by silica gel column chromatography using a gradient of DCM and MeOH as eluent to afford complexes **Au1-Au3**.

Au1: Yellow solid; Yield 50.2%; Purity > 95%; ^{1}H NMR (500 MHz, Chloroform-*d*) δ 7.13 (d, *J* = 2.0 Hz, 1H, CH=CH), 6.98 (d, *J* = 1.7 Hz, 1H, CH=CH), 6.19 (d, *J* = 3.3 Hz, 1H, $C=CH_2$), 5.49 (d, *J* = 3.1 Hz, 1H, $C=CH_2$), 4.99 (d, *J* = 17.4 Hz, 1H), 4.89 (d, *J* = 17.4 Hz, 1H), 4.23 (q, *J* = 7.3 Hz, 2H), 3.78 (t, *J* = 10.1 Hz, 1H, OCH), 3.18 (d, *J* = 10.1 Hz, 1H), 2.71 – 2.62 (m, 1H), 2.52 – 2.39 (m, 2H), 2.34 – 2.17 (m, 4H), 2.14 – 2.01 (m, 2H), 1.71 (s, 3H, CH_3), 1.55 (s, 3H, CH_3), 1.46 (t, *J* = 7.3 Hz, 3H, CH_3). ^{13}C NMR (126 MHz, Chloroform-*d*) δ 175.41, 170.38, 165.96, 139.26, 132.24, 129.32, 122.28, 120.16, 119.35, 91.31, 83.08, 56.43, 52.82, 49.88, 46.58, 36.41, 35.07, 30.39, 25.98, 24.29, 18.97, 16.54. ESI-MS(+)[m/z] calcd for $C_{22}H_{28}AuN_2O_4$ $[M-Br]^+$ (m/z): 581.17; Found, 581.16.

Au2: White solid; Yield 88.4%; Purity > 95%; ^{1}H NMR (500 MHz, Chloroform-*d*) δ 7.15 (dd, *J* = 11.6, 8.5 Hz, 4H, ArH), 6.87 (dd, *J* = 8.7, 3.7 Hz, 4H, ArH), 6.17 (d, *J* = 3.3 Hz, 1H, $C=CH_2$), 5.45 (d, *J* = 3.1 Hz, 1H, $C=CH_2$), 4.85 (d, *J* = 17.1 Hz, 1H), 4.76 (d, *J* = 17.3 Hz, 1H), 4.27 – 4.19 (m, 2H), 3.79 (d, *J* = 6.3 Hz, 6H, OCH_3), 3.71 (t, *J* = 10.1 Hz, 1H, OCH), 3.15 (d, *J* = 10.9 Hz, 1H), 2.69 – 2.60 (m, 1H), 2.52 – 2.39 (m, 2H), 2.31 – 2.04 (m, 6H), 1.71(s, 3H, CH_3), 1.46 (s, 3H, CH_3), 1.45 – 1.41 (m, 3H, CH_3). ^{13}C NMR (126 MHz, Chloroform-*d*) δ 174.28, 170.17, 166.78, 160.43, 160.29, 139.51, 132.14, 132.07, 131.82, 130.49, 130.01, 119.82, 119.21, 118.95, 114.58, 114.42, 91.37, 82.93, 56.62, 55.40, 50.88, 50.18, 44.36, 36.59, 34.97, 30.49, 29.84, 26.03, 24.33, 18.89, 17.04. ESI-MS(+)[m/z] calcd for $C_{36}H_{40}AuN_2O_6$ $[M-Br]^+$ (m/z): 793.26; Found, 793.24.

Au3: White solid; Yield 57.4%; Purity > 95%; ^{1}H NMR (500 MHz, Chloroform-*d*) δ 7.27 – 7.18 (m, 4H, ArH), 7.07 (td, *J* = 8.6, 3.8 Hz, 4H, ArH), 6.18 (d, *J* = 3.3 Hz, 1H, $C=CH_2$), 5.47 (d,

J = 3.0 Hz, 1H, C=CH_2), 4.82 (q, *J* = 17.3 Hz, 1H), 4.27 – 4.10 (m, 2H), 3.71 (t, *J* = 10.1 Hz, 1H, OCH), 3.11 (d, *J* = 10.4 Hz,1H), 2.67 – 2.59 (m, 1H), 2.52 – 2.40 (m, 2H), 2.31 – 2.18 (m, 4H), 2.10 (t, *J* = 9.4 Hz, 2H), 1.70 (s, 3H, CH_3), 1.43 (s, 3H, CH_3), 1.37 (s, 3H, CH_3). ^{13}C NMR (126 MHz, Chloroform-*d*) δ 175.39, 170.15, 166.33, 163.50 (d, *J* = 250.7 Hz), 163.36 (d, *J* = 252.0 Hz), 139.34, 132.74 (t, *J* = 7.6 Hz), 132.27, 131.36, 130.17, 129.62, 122.91, 119.20, 116.49 (dd, *J* = 21.9, 16.6 Hz), 91.46, 82.91, 56.71, 51.05, 50.04, 44.53, 36.52, 35.01, 30.65, 30.47, 30.26, 29.85, 26.02, 24.34, 18.72, 17.00. ESI-MS(+)[m/z] calcd for $C_{34}H_{34}AuF_2N_2O_4$ [M-Br]$^+$ (m/z): 769.22; Found, 769.20.

Syntheses of Au4-Au6

Complexes **Au1-Au3** (1.0 equiv) and the corresponding ligands **c1-c3** (1.0 equiv) were dissolved in DCM. Potassium carbonate (K_2CO_3, 1.3 equiv) and potassium hexafluorophosphate (KPF_6, 10.0 equiv) were then added under Ar, and the reaction mixture was stirred at room temperature in the darkness for 24 h. The mixture was subsequently filtered, and the filtrate was purified by silica gel column chromatography using a gradient of DCM and MeOH as eluent to afford complexes **Au4-Au6**.

Au4: White solid; Yield 56.5%; Purity > 95%; ^{1}H NMR (500 MHz, Chloroform-*d*) δ 7.30 – 7.26 (m, 2H, CH=CH), 7.13 – 7.09 (m, 2H, CH=CH), 6.15 (d, *J* = 3.4 Hz, 2H, C=CH_2), 5.48 (d, *J* = 3.1 Hz, 2H, C=CH_2), 4.96 (s, 4H), 4.33 – 4.20 (m, 4H), 3.75 (t, *J* = 10.1 Hz, 2H, OCH), 3.13 – 3.06 (m, 2H), 2.71 – 2.62 (m, 2H), 2.50 – 2.40 (m, 4H), 2.30 – 2.15 (m, 7H), 2.09 (d, *J* = 9.9 Hz, 2H), 2.05 – 1.92 (m, 3H), 1.62 (s, 6H, CH_3), 1.55 – 1.48 (m, 12H, CH_3). ^{13}C NMR (126 MHz, Chloroform-*d*) δ 184.40, 170.37, 166.18, 139.27, 132.45, 129.13, 123.42, 121.18, 119.33, 91.26, 83.08, 56.56, 52.96, 49.78, 46.66, 36.39, 34.96, 30.42, 29.84, 25.96, 24.32, 18.83, 16.90. ESI-MS(+)[m/z] calcd for $C_{44}H_{56}AuN_4O_8$ [M-PF_6]$^+$ (m/z): 965.38; Found, 965.49.

Au5: White solid; Yield 65.9%; Purity > 95%; ^{1}H NMR (500 MHz, Chloroform-*d*) δ 7.15 (dd, *J* = 15.1, 8.2 Hz, 8H, ArH), 6.89 (t, *J* = 5.9 Hz, 8H, ArH), 6.16 (d, *J* = 3.1 Hz, 2H, C=CH_2), 5.48 (s, 2H, C=CH_2), 4.95 (d, *J* = 17.5 Hz, 2H), 4.83 (d, *J* = 17.0 Hz, 2H), 4.22 (q, *J* = 7.7, 7.1 Hz, 4H), 3.80 (d, *J* = 5.7 Hz, 12H, OCH_3), 3.70 (t, *J* = 10.1 Hz, 2H, OCH), 3.01 (d, *J* = 10.2 Hz, 2H), 2.62 (t, *J* = 10.6 Hz, 2H), 2.46 – 2.31 (m, 6H), 2.29 – 2.13 (m, 6H), 2.09 (d, *J* = 13.3 Hz, 2H), 1.82 – 1.74 (m, 2H), 1.72 (s, 6H, CH_3), 1.53 (s, 6H, CH_3), 1.46 – 1.38 (m, 6H, CH_3). ^{13}C NMR (126 MHz, Chloroform-*d*) δ 183.94, 170.05, 166.96, 160.55, 139.29, 132.14, 132.06, 129.55, 119.36, 118.96, 114.63, 114.59, 91.39, 82.77, 56.66, 55.50, 55.47, 51.04, 50.29, 44.43, 36.59, 34.93, 29.84, 25.95, 24.33, 18.93, 17.59. ESI-MS(+)[m/z] calcd for $C_{72}H_{80}AuN_4O_{12}$ [M-PF_6]$^+$ (m/z): 1389.54; Found, 1389.68.

Au6: White solid; Yield 86.6%; Purity > 95%; ^{1}H NMR (500 MHz, Chloroform-*d*) δ 7.54 (d, *J* = 8.6 Hz, 1H, ArH), 7.35 (s, 3H, ArH), 7.11 – 7.01 (m, 12H, ArH), 6.15 (d, *J* = 3.2 Hz, 2H, C=CH_2), 5.46 (d, *J* = 3.0 Hz, 2H, C=CH_2), 5.02 – 4.86 (m, 4H), 4.26 (s, 4H), 3.69 (t, *J* = 10.1 Hz, 2H, OCH), 3.04 (d, *J* = 10.3 Hz, 2H), 2.63 (t, *J* = 10.3 Hz, 2H), 2.41 (dd, *J* = 16.3, 8.7 Hz, 2H), 2.37 – 2.28 (m, 2H), 2.27 – 2.18 (m, 8H), 2.01 (q, *J* = 6.7 Hz, 4H), 1.69 (s, 6H, CH_3), 1.48 (s, 6H, CH_3), 1.44 – 1.41 (m, 6H, CH_3). ^{13}C NMR (126 MHz, Chloroform-*d*) δ 184.61, 170.21, 164.35, 139.23, 136.44, 133.17 (dd, *J* = 1.3, 7.6 Hz), 132.37, 131.37, 129.00, 119.34, 116.79 (t, *J* = 22.7 Hz), 92.09, 83.03, 56.12, 49.61, 49.25, 43.83, 35.98, 34.95, 30.31, 29.83, 25.99, 24.25, 18.76, 14.79. ESI-MS(+)[m/z] calcd for $C_{68}H_{68}AuF_4N_4O_8$ [M-PF_6]$^+$ (m/z): 1341.46; Found, 1341.61.

Syntheses of Au7-Au10

Complexes **d1-d3** (1.0 equiv) and ligand **c1** (1.0 equiv) were dissolved in acetone. K_2CO_3 (1.3 equiv) and KPF_6 (10.0 equiv) were then added. The reaction mixture was stirred at room temperature under Ar in the darkness for 48 h. After completion, the mixture was subsequently filtered, and the filtrate was purified by silica gel column chromatography using a gradient of DCM and MeOH as eluent to afford complexes **Au7-Au9**. Complex **Au10** was synthesized by a similar method.

Au7: White solid; Yield 47.0%; Purity > 95%; ^{1}H NMR (500 MHz, Chloroform-*d*) δ 7.28 (s, 1H, CH=CH), 7.10 (d, *J* = 7.8 Hz, 3H, CH=CH), 6.16 (t, *J* = 3.6 Hz, 1H, C=CH_2), 5.49 (t, *J* = 3.2 Hz, 1H, C=CH_2), 4.95 (d, *J* = 3.9 Hz, 2H), 4.31 – 4.20 (m, 6H), 3.75 (t, *J* = 10.1 Hz, 1H, OCH), 3.10 (d, *J* = 10.4 Hz, 1H), 2.67 (t, *J* = 10.1 Hz, 1H), 2.51 – 2.40 (m, 2H), 2.32 – 2.18 (m, 4H), 2.10 (d, *J* = 13.3 Hz, 1H), 2.03 – 1.92 (m, 1H), 1.69 (s, 3H, CH_3), 1.57 – 1.49 (m, 12H, CH_3). ^{13}C NMR (126 MHz, Chloroform-*d*) δ 184.72, 182.56, 170.40, 166.19, 139.21, 132.54, 129.03, 123.44, 121.22, 121.18, 119.40, 91.31, 83.07, 58.63, 56.63, 52.92, 49.80, 46.65, 36.43, 34.96, 30.42, 29.83, 25.94, 24.33, 18.83, 18.78, 18.57, 17.06, 16.90. ESI-MS(+)[m/z] calcd for $C_{29}H_{40}AuN_4O_4$ [M-PF_6]$^+$ (m/z): 705.27; Found, 705.38.

Au8: White solid; Yield 36.9%; Purity > 95%; ^{1}H NMR (500 MHz, Chloroform-*d*) δ 7.31 (d, *J* = 1.9 Hz, 1H, CH=CH), 7.14 (d, *J* = 8.5 Hz, 5H, CH=CH, ArH), 6.94 – 6.85 (m, 4H, ArH), 6.16 (d, *J* = 3.3 Hz, 1H, C=CH_2), 5.48 (d, *J* = 3.0 Hz, 1H, C=CH_2), 5.07 – 4.93 (m, 2H), 4.31 (q, *J* = 7.3 Hz, 2H), 4.24 – 4.13 (m, 4H), 3.80 (s, 6H, OCH_3), 3.74 (t, *J* = 10.1 Hz, 1H, OCH), 3.11 (d, *J* = 10.4 Hz, 1H), 2.70 (t, *J* = 11.2 Hz, 1H), 2.52 – 2.42 (m, 2H), 2.36 – 2.15 (m, 4H), 2.09 (d, *J* = 13.7 Hz, 1H), 2.04 – 1.94 (m, 1H), 1.69 (s, 3H, CH_3), 1.57 – 1.52 (m, 6H, CH_3), 1.36 (t, *J* = 7.2 Hz, 6H, CH_3). ^{13}C NMR (126 MHz, Chloroform-*d*) δ 184.55, 182.05, 170.41, 166.26, 160.34,

139.37, 132.00, 131.62, 129.24, 123.55, 121.31, 119.64, 119.23, 114.52, 91.27, 83.14, 56.69, 55.44, 52.97, 49.77, 46.67, 44.28, 36.43, 34.92, 30.47, 26.01, 24.33, 18.75, 17.59, 16.96. ESI-MS(+)[m/z] calcd for $C_{43}H_{52}AuN_4O_6$ [M-PF_6]$^+$ (m/z): 917.36; Found, 917.46.

Au9: White solid; Yield 56.2%; Purity > 95%; ^{1}H NMR (500 MHz, Chloroform-*d*) δ 7.30 (d, *J* = 1.9 Hz, 1H, CH=CH), 7.23 (dd, *J* = 8.4, 5.4 Hz, 4H, ArH), 7.11 (d, *J* = 1.5 Hz, 1H, CH=CH), 7.10 – 7.05 (m, 4H, ArH), 6.16 (d, *J* = 3.2 Hz, 1H, C=CH_2), 5.49 (d, *J* = 3.0 Hz, 1H, C=CH_2), 5.07 – 4.94 (m, 2H), 4.35 – 4.14 (m, 6H), 3.75 (t, *J* = 10.1 Hz, 1H, OCH), 3.12 (d, *J* = 10.9 Hz, 1H), 2.70 (t, *J* = 10.7 Hz, 1H), 2.52 – 2.42 (m, 2H), 2.33 – 2.16 (m, 4H), 2.09 (d, *J* = 13.6 Hz, 1H), 2.01 (dd, *J* = 12.5, 9.1 Hz, 1H), 1.69 (s, 3H, CH_3), 1.57 – 1.52 (m, 6H, CH_3), 1.42 – 1.32 (m, 6H, CH_3). ^{13}C NMR (126 MHz, Chloroform-*d*) δ 184.44, 182.92, 170.43, 166.27, 163.36 (d, *J* = 250.7 Hz), 139.37, 132.72 (dd, *J* = 8.5, 2.6 Hz), 132.41, 131.17, 129.23, 123.47, 123.45, 123.42, 121.24, 119.25, 116.44 (d, *J* = 21.8 Hz), 91.30, 83.16, 56.64, 52.96, 49.78, 46.71, 44.46, 36.40, 34.93, 30.46, 26.00, 24.33, 18.79, 17.47, 17.43, 16.94. ESI-MS(+)[m/z] calcd for $C_{41}H_{46}AuF_2N_4O_4$ [M-PF_6]$^+$ (m/z): 893.32; Found, 893.44.

Au10: Brown solid; Yield 59.8%; Purity > 95%; ^{1}H NMR (500 MHz, Chloroform-*d*) δ 7.50 – 7.40 (m, 3H, ArH), 7.28 (d, *J* = 1.9 Hz, 1H, ArH), 7.26 – 7.22 (m, 2H, ArH), 7.21 – 7.16 (m, 6H, ArH), 7.14 – 7.05 (m, 14H, ArH), 7.05 – 6.95 (m, 6H, ArH), 5.23 (s, 2H, C=CH_2), 4.24 (q, *J* = 6.3 Hz, 4H), 3.98 (q, *J* = 7.2 Hz, 4H, N=CH_2), 3.57 (t, *J* = 5.9 Hz, 1H, OCH), 3.44 (q, *J* = 6.7 Hz, 2H), 2.00 – 1.91 (m, 2H), 1.89 – 1.82 (m, 3H), 1.39 (t, *J* = 7.2 Hz, 3H), 1.33 (s, 1H), 1.18 (t, *J* = 7.2 Hz, 6H). ^{13}C NMR (126 MHz, Chloroform-*d*) δ 184.62, 181.93, 167.72, 163.44 (d, *J* = 252 Hz), 145.25, 143.14, 143.07, 137.10, 132.79, 132.68, 132.61, 132.53, 131.40, 131.26, 131.16, 128.07, 127.91, 127.02, 126.95, 124.20, 124.06, 123.28, 122.23, 116.60, 116.54, 116.43, 116.36, 65.52, 52.47, 44.48, 44.29, 33.71, 32.07, 29.84, 29.80, 29.50, 29.18, 27.19, 25.98, 17.42, 17.37. ESI-MS(+)[m/z] calcd for $C_{65}H_{60}AuF_2N_4O_4$ [M-PF_6]$^+$ (m/z): 1196.18; Found, 1196.11.

Stability Analysis

Au9 was dissolved in a mixture of (Dimethyl sulfoxide)-d_6 (DMSO-d_6, 450 μL) and deuterium oxide (D_2O, 50 μL) ($v_1 : v_2$ = 9 : 1); **Au9** was incubated with either glutathione (GSH) or L-ascorbic acid (VC) in a mixture of DMSO-d_6 (450 μL) and D_2O (50 μL) ($v_1 : v_2$ = 9 : 1); **Au9** was dissolved in DMSO-d_6 (250 μL) and *N*-acetylcysteine (NAC) was dissolved in acetonitrile-d_3 (250 μL), which were mixed; **Au9** dissolved in a mixture of DMSO-d_6 (475 μL) and D_2O (25 μL) containing mouse plasma (100 μL). Sample solutions were prepared such that **Au9**, GSH,

and VC each reached a final concentration of 5.0 mM. Then the ^{1}H NMR spectra of them were recorded at different times at 298 K (30 min, 6 h, 12 h, 24 h, 48 h and 72 h).

Biological Methods

Cell Culture and Conditions

The human-derived HepG2 and Huh7 cell lines, as well as the murine Hepa 1-6 cell line, were cultured in DMEM (Gibco, Thermo Fisher Scientific Inc., the United States). The THP-1 cell line was cultured in RPMI1640 medium (Gibco, Thermo Fisher Scientific Inc., the United States). All cell lines were subcultured in medium supplemented with 10% fetal bovine serum (Genial, the United States).

Cell viability (CCK-8 assay)

Cell viability was assessed using the CCK-8 assay. Cells were seeded at the desired density into a 96-well plate, with 100 μL of DMEM containing 10% fetal bovine serum added to each well. Overnight culture was performed until cells adhered. Subsequently, specific complexes at varying concentrations were added, and cells were cultured for designated periods (24 h, 48 h, or 72 h). Each well received 10 μL of CCK-8 solution (C6005, NCM) and was incubated for an additional 1-2 h. Absorbance was measured at 450 nm using a microplate reader. Cell viability (%) = (Experimental group A_{450} – Blank group A_{450}) / (Control group A_{450} – Blank group A_{450}) × 100%.

Quantification of Gold in Subcellular Fractions

HepG2 cells were seeded in 10 cm culture dishes at a density of 1 × 10^6 cells per dish and incubated overnight to allow cell attachment. The cells were then treated with the complexes for an additional 24 h. Subsequently, the cells were harvested by trypsinization and centrifuged to obtain cell pellets. Mitochondrial and nuclear fractions were isolated using a Mitochondria/Nucleus Extraction Kit (KGA3107-50, Keygen Biotech, China), while the endoplasmic reticulum fraction was isolated using an endoplasmic reticulum separation kit. The obtained subcellular fractions were sequentially digested by adding 100 μL of concentrated nitric acid and heating at 95 °C for 1 h, followed by the addition of 50 μL of 30% hydrogen peroxide and further heating at 95 °C for 1 h. Finally, 75 μL of concentrated hydrochloric acid was added, and the samples were incubated at 37 °C for 0.5 h. After digestion, the remaining solutions were diluted to a final volume of 1 mL with double-distilled water. The Au content in

each sample was determined using inductively coupled plasma mass spectrometry (ICP-MS; Thermo X Series 2, the United States).

Fluorescent Au10 and Organelle Colocalization Analysis

HepG2 cells were seeded in confocal dishes and cultured overnight. Complexes were added, and cells were incubated for an additional 24 h. After incubating with ER-Tracker Red (C1041S, Beyotime, China), Mito-Tracker Red (Beyotime, China), and Lyso-Tracker Red (C1046, Beyotime, China) for 30 min, the staining solution was discarded and washed with DMEM. Capture images using a laser confocal microscope (Leica, TCS SP8, Germany). The ImageJ was used to investigate the Pearson correlation analysis.

Western Blotting

HepG2 cells were treated with the indicated complexes for 24 h. After treatment, the cells were washed with pre-chilled PBS and lysed in RIPA buffer supplemented with phosphatase and protease inhibitors at a ratio of 100 : 1 : 1 (RIPA buffer: phosphatase inhibitor: protease inhibitor). The cell lysates were subsequently sonicated to facilitate protein extraction. Protein concentrations were determined using a BCA Protein Assay Kit (WB6501, NCM, China), and equal amounts of protein were loaded for subsequent analysis.

The following antibodies were used in this study:

TXNRD1 (11117-1-AP, Proteintech, China), GAPDH (10494-1-AP, Proteintech, China), GPX4 (30388-1-AP, Proteintech, China), SLC7A11 (YM8243, Immunoway, China), β-actin (GB15003-100, Servicbio, China), STAT1 (10144-2-AP, Proteintech, China), Vinculin (ET1705-94, Huabio, China), p-HMGCR (PC40635, ABmart, China), HMGCR (13533-1-AP, Proteintech, China), α-Tubulin (66031-1-Ig, Proteintech, China), SREBF2 (28212-1-AP, Proteintech, China), p-AMPK (WL05103, Wanleibio, China), AMPK (WL02254, Wanleibio, China), Secondary antibodies (SA00001-2, Proteintech, China).

Fluorescent Probe Staining

HepG2 cells were treated with the indicated complexes for 24 h and subsequently stained with the corresponding fluorescent probes. After staining, the cells were washed three times with DMEM, and fluorescence images were acquired using a laser scanning confocal microscope (Leica TCS SP8, Germany).

This assay employed the following probes: TrxR Probe (Created Independently-DA (S0034, Beyotime, China), DHE (S0063, Beyotime, China), Ferfluor-1 (Kindly provided by the laboratories of Prof. Yong Wang and Prof. Xue Qiu), JC-1 (C2005, Beyotime, China), CD11b (C67F154, Thermo Fisher Scientific Inc., the United States), CD86 (MA1-10299, Thermo Fisher Scientific Inc., the United States), DiI (C1036, Beyotime, China).

Immunofluorescence Staining

Sterilized coverslips were placed in 24-well plates, and HepG2 cells were uniformly seeded at a density of 5 × 10^4 cells per well and cultured overnight to allow cell attachment. After treatment with the indicated compounds for 24 h, the culture medium was removed, and the cells were fixed with 4% paraformaldehyde at room temperature for 15 min. The cells were subsequently permeabilized and blocked with PBS containing 1% BSA and 0.1% Triton X-100. The coverslips were incubated with the appropriate primary antibodies at 4 °C overnight, followed by incubation with the corresponding secondary antibodies. The nuclei were then counterstained with DAPI in the dark for 10 min. Finally, the coverslips were mounted using an antifade mounting medium. Between each step, the cells were washed three times with PBS or TBS for 10 min each.
The following antibodies were used in this assay: CHOP (15204-1-AP, Proteintech, China), p-eif2s1 (YM8728, ImmunoWay, China), HMGB1 (82973-1-RR, Proteintech, China), CRT (27298-1-AP, Proteintech, China), Secondary antibodies (SA00013-4; SA00013-2, Proteintech, China).

TrxR Enzymatic Activity Assay

Different concentrations of the indicated complexes were incubated with 1.8 U of purified TrxR (25 μL per well) in a 96-well plate. Subsequently, 225 μL of the reaction mixture containing BSA (20 mg/mL), EDTA-2Na (500 mM), and NADPH (40 mg/mL), diluted 1:1 with PBS and water, was added to each well. Finally, 25 μL of DTNB solution (25 mg/mL) was added, and the absorbance at 405 nm was immediately monitored. Purified TrxR activity was determined from the linear increase in absorbance at 405 nm over time.

Cellular Thermal Shift Assay (CETSA)

HepG2 cells in the logarithmic growth phase were harvested, subjected to repeated freeze-thaw cycles using liquid nitrogen and 37 °C for protein extraction, and centrifuged at high speed to obtain the supernatant. Complexes were added and incubated for 2 h. Samples were then heated for 3 min using a PCR instrument at a preset temperature gradient. Finally, protease

inhibitors and loading buffer were added, followed by heating at 100 °C for 10 min to obtain samples ready for subsequent Western blot analysis.

RNA Sequencing and Analysis

Huh7 cells were uniformly seeded at a density of 3 × 10^6 cells per dish in 100 mm dishes and co-cultured with specific complexes for 24 h. The medium was removed, and PBS was added for gentle washing for 1 min, repeated twice. One milliliter of Trizol lysis buffer was added, and the mixture was vortexed until homogeneous. Samples were stored at -80 °C. Samples were subsequently sent to LC-Bio Technologies Co., Ltd. (China) for transcriptomic sequencing. All transcriptomic analyses were performed using the OmicsStudio cloud platform.

Validation of selected genes by qPCR

RNA extraction and cDNA reverse transcription were performed using the MolPure® TRIeasy Plus Total RNA Kit and Hifair® III 1st Strand cDNA Synthesis SuperMix for qPCR (gDNA digester plus) (Yeasen Biotechnology Shanghai Co., Ltd., China). One microgram of extracted RNA was reverse transcribed into cDNA. Quantitative PCR analysis was performed on a QuantStudio 3 Real-Time Fluorescent Quantitative PCR System (Thermo Fisher Scientific Inc., the United States) using Hieff UNICON® Advanced qPCR SYBR Master Mix (Yeasen Biotechnology Shanghai Co., Ltd., China).

GAPDH (Forward: 5’-GTCAAGCCTGAGAACGGGAA-3’; Reverse: 5’-AAATGAGCCCCA GCCTTCTC-3’)

SLC7A11 (Forward: 5’-CTTTGTTGCCCTCTCCTGCTTTG-3’; Reverse: 5’-GAGTGTGCTTGC GGACATGAATC-3’)

CP (Forward: 5’-CCAGGTCCAGGAGTGTAACAAGTC-3’; Reverse: 5’-TTCCTCAGCGGCAAT GTAGTAGTG-3’)

ACSL1 (Forward: 5’-ACGAAGATCCGCACTACTTGACAG-3’; Reverse: 5’-GGGTTGGTCTGG TTTCCGAGAG-3’)

SAT2 (Forward: 5’-AGGGGTCTAGTTGCCGTTTGC-3’; Reverse: 5’-CTTCACCCGAATCA GCCTCAGG-3’)

ACSL4 (Forward: 5’-TCCAAGTAGACCAACGCCTTCAG-3’; Reverse: 5’-CGGTCCCAGTCCA GGTATTCTTTC-3’)

SLC3A2 (Forward: 5’-TGCAAGCTGGCGTGGATGG-3’; Reverse: 5’-GCTGAAGGTCGGAGG AGTTAGTC-3’)

HMGCS2 (Forward: 5’-AGCGTCTGTTGACTCCAGTGAAG-3’; Reverse: 5’-GCAGGCGAGCA

GGTGTGAG-3')

HMGCS1 (Forward: 5'-GCTCTTGGGATGGACGGTATGC-3'; Reverse: 5'-GCTACTGCTCCAA CTCCACCTG-3')

APOA4 (Forward: 5'-TGGAGAAGGACCTGAGGGACAAG-3'; Reverse: 5'-TGCTGCTCCTGC TGCTGTTC-3')

STAR (Forward: 5'-TTCACCCGTTGGCTGGAAGTC-3'; Reverse: 5'-AGGACCTGGTTGATGA TGCTCTTG-3')

CD36 (Forward: 5'-CGGAACTGTGGGCTCATCGC-3'; Reverse: 5'-AGCAGGTCTCCAACT GGCATTAG-3')

APOC2 (Forward: 5'-AAAGACAGCCGCCCAGAACC-3'; Reverse: 5'-CCCTTCAGCACAGAAA GAACTTGG-3')

TSPO (Forward: 5'-CTTGGTGGATCTCCTGCTGGTC-3'; Reverse: 5'-AGTAGTTGAGTGTGGTCGTGAAGG-3')

LRP2 (Forward: 5'-GCAGCAGCAGTGAGTTCCAATG-3'; Reverse: 5'-ACAAGAGGCAGGTTC ATCAGAGG-3')

ABCB11 (Forward: 5'-GAGGCGGTTCATTGAAGCACTTG-3'; Reverse: 5'-GCACTGGGCAAA GGCAAAGC-3')

ABCG8 (Forward: 5'-GCTGGTGTGGCTGGTGGTC-3'; Reverse: 5'-CGGCGAGGTAGAAGG AGTTGTAG-3')

APOA1 (Forward: 5'-GACAGCGTGACCTCCACCTTC-3'; Reverse: 5'-CTTCACCTCCTCCAG ATCCTTGC-3')

The construction of siRNA in HepG2 cells

Cells were seeded in 6-well plates at a density of 2 × 10^5 cells per well and cultured overnight to allow cell attachment. For siRNA transfection, 42.5 μL of Buffer (GenePharma, China) was mixed with 4 μL of siRNA (GenePharma, China) to prepare the siRNA premix. Subsequently, 8 μL of Plus transfection reagent (GenePharma, China) was added to each tube of the siRNA premix and thoroughly mixed to form the siRNA/Plus complexes. The resulting siRNA/Plus complexes were added to the cells, followed by incubation for 24 h. The transfection efficiency was subsequently evaluated by Western blot analysis.

STAT1 (Sense: 5'-GCGUAAUCUUCAGGAUAAUTT-3'; Antisense: 5'-AUUAUCCUGAAGAUU ACGCTT-3')

AMPK (Sense: 5'-CCUCAAGCUUUUCAGGCAUTT-3'; Antisense: 5'-AUGCCUGAAAAGCUU GAGGTT-3')

(Sense: 5’-UUAAACUGUACCAGGUCAUTT-3’; Antisense: 5’-AUGACCUGGUACAGUUUAAT T-3’)

HepG2-GFP Construction Assay

Cells were seeded in 6-well plates at a density of 2 × 10^5 cells per well and cultured overnight to allow cell attachment, reaching approximately 40–60% confluence at the time of transduction. Lentiviral transduction was performed at a multiplicity of infection (MOI) of 9, and the required viral volume was calculated according to the following formula: viral volume (μL) = MOI × number of cells / viral titer (TU/mL) × 1000. The lentivirus was thawed on ice before use. Prior to transduction, the culture medium was replaced with fresh medium, followed by the addition of the calculated volume of lentivirus and Polybrene (8 μg/mL). Cell morphology and viability were examined after 12 h. In the absence of obvious cytotoxicity, transduction was continued for 24 h. The medium was then replaced with fresh culture medium, and the cells were further cultured for up to 72 h. Fluorescence expression was examined using a fluorescence microscope (Leica, Germany). Subsequently, puromycin (1 μg/mL; ST551, Beyotime, China) was added, and the cells were cultured for at least 4 days to select stable transductants.

HepG2 and Macrophage Co-culture Assay

THP-1 cells were seeded in confocal dishes at a density of 1 × 10^5 cells per dish and cultured in RPMI 1640 medium supplemented with 10% FBS and 100 ng/mL PMA for 24 h to induce their differentiation from suspension cells into adherent macrophage-like cells. The medium was then replaced with fresh medium, and the cells were further cultured for 24 h to obtain M0 macrophages. Subsequently, M0 macrophages were cultured for 24 h in medium containing the culture supernatants collected from drug-treated HepG2-GFP cells and then stained with 10 μM DiI (a red fluorescent cell membrane probe; C1036, Beyotime, China) for 20 min. HepG2-GFP cells were harvested by trypsinization and added to the macrophages at a tumor cell-to-macrophage ratio of 2 : 1, followed by co-culture for 12 h. Tumor phagocytosis was visualized using laser confocal microscopy (Leica TCS SP8, Germany), flow cytometry (Beckman Coulter MoFlo XDP, the United States), or long-term dynamic live-cell imaging and data analysis systems (Essen Bioscience IncuCyte Zoom). After 12 h of co-culture, the supernatants were discarded, and the macrophages were washed twice with PBS to remove non-engulfed cell debris. Total RNA was then extracted, and the mRNA expression levels of TNF, IL-1β, and IL-6 were determined by RT–qPCR. the United States), or a long-term live-cell imaging and analysis system (IncuCyte ZOOM, Essen BioScience, the United States). After 12 h of co-

culture, the supernatants were removed, and the macrophages were washed twice with PBS to remove non-engulfed cells and cellular debris. Total RNA was subsequently extracted, and the mRNA expression levels of TNF, IL-1β, and IL-6 were determined by RT–qPCR.

TNF-α (Forward: 5'- CCCTCCTTCAGACACCCT-3'; Reverse: 5'- GGTTGCCAGCACTTCACT-3')

IL-1β (Forward: 5'- TGTGCTGAATGTGGACTCA-3'; Reverse: 5'- ACAAAAGGGCTGGGGAT-3')

IL-6 (Forward: 5'- TGTGTGAAAGCAGCAAAGA-3'; Reverse: 5'- ACCAGGCAAGTCTCCTCA-3')

Intracellular ATP Content Detection

Cells were seeded in 6-well plates at a density of 1 × 10^6 cells per well and cultured overnight to allow cell attachment. The cells were then treated with the indicated complexes for 24 h, after which both the cells and culture supernatants were collected. Intracellular and extracellular ATP levels were determined using an ATP Assay Kit (S0027, Beyotime, China) according to the manufacturer's instructions. ATP levels were quantitatively measured using a multimode microplate reader (Infinite M1000 Pro, Tecan, Switzerland).

Intracellular Cholesterol Level Detection

Cells were seeded in 6-well plates at a density of 1 × 10^6 cells per well and cultured overnight to allow cell attachment. The cells were then treated with the indicated complexes for 24 h. Intracellular cholesterol levels were determined using an Amplex Red Cholesterol and Cholesteryl Ester Assay Kit (S0211S, Beyotime, China) according to the manufacturer's instructions. The absorbance was measured using a microplate reader (AMR-100, Allsheng, China), and intracellular cholesterol levels were subsequently quantified.

LDH Release Detection

THP-1 cells were seeded in 12-well plates at a density of 2 × 10^5 cells per well and cultured in RPMI 1640 medium supplemented with 10% FBS and 100 ng/mL PMA for 24 h to induce cell adherence. The medium was then replaced with fresh medium, and the cells were further cultured for 24 h to obtain M0 macrophages. For M1 polarization, M0 macrophages were stimulated with 100 ng/mL LPS and 20 ng/mL IFN-γ for 24 h. For M2 polarization, M0 macrophages were stimulated with 20 ng/mL IL-4 and 20 ng/mL IL-13 for 24 h. The resulting M1 or M2 macrophages were subsequently treated with different concentrations of the indicated

complexes for 24 h. LDH release was determined using a Lactate Dehydrogenase Release Assay Kit (WST-8 method; C0019S, Beyotime, China) according to the manufacturer's instructions.

In Vivo Evaluation of Anti-tumor and Anti-tumor Immunity

C57BL/6 mice (Jiangsu Qinglongshan Biotechnology Co., Ltd., China) received subcutaneous inoculation of Hepa 1-6 cells (2×10^6 cells/mouse) in the left axillary region. When tumor volume reached 50 mm^3, mice were randomly assigned to four groups: Control, **Au9**, **Au9** + anti-PD-L1, and OXA. For bilateral tumor models, secondary tumors were implanted in the right axillary region. Two days later, treatments were administered according to group assignments: **Au9** (5 mg/kg) and OXA (5 mg/kg) via intraperitoneal injection every other day (every 4 days for bilateral tumor models, administered intratumorally); anti-PD-L1 (2.5 mg/kg) via intraperitoneal injection every 3 days (every 3 days for bilateral tumor models, administered intratumorally); and the control group received an equal volume of saline.

The unilateral subcutaneous tumor-bearing model was used to analyze systemic pharmacodynamic efficacy (anti-tumor and TIME-remodeling) of **Au9** treatment. The bilateral subcutaneous tumor-bearing model was designed to investigate the abscopal effect (anti-tumor immune response) and in vivo "sensor" of the contralateral untreated tumor for systemic immune activation.

Throughout the experiment, the mouse body weight and tumor volume were continuously monitored. Tumor volume (V) was calculated as $1/2 \times$ major axis (mm) $\times$ minor axis^2. The experiment was terminated when the mouse tumor volume approached 2000 mm^3, and all mice were humanely euthanized. Tumor tissue, blood samples, and major organs were collected. Portions of tumor tissue from each group were digested with 1 mg/mL collagenase IV (Shanghai Yuanye Bio-Technology Co., Ltd.) and 0.2 mg/mL DNase (Shanghai Yuanye Bio-Technology Co., Ltd.) to prepare a single-cell suspension. Red blood cells were subsequently lysed using red blood cell lysis buffer (C3702, Beyotime, China). The single-cell suspensions were stained with corresponding flow cytometry antibodies and analyzed by flow cytometry (Beckman Coulter, MoFlo XDP). Separate tumor tissue and major organ samples were fixed in 4% paraformaldehyde and sent to Wuhan Servicebio Technology Co., Ltd. for paraffin embedding, H&E staining, and immunohistochemical and fluorescence analysis. Mass cytometry samples were processed into single-cell suspensions and sent to the Chinese Academy of Sciences Shanghai Institute of Materia Medica.

This experiment utilized the following antibodies: CD4, CD8, CD279, CD45, CD11c, CD80,

CD86, CD11b and Ly6G/6C.

All animal experiments were approved by the Institutional Animal Care and Use Committee (IACUC) of Nanjing University of Chinese Medicine (approval no. 202503A091) and performed in accordance with the ARRIVE guidelines.

Statistical Analysis

All the results of the experiment were expressed as mean ± SD, and statistical analysis and drawing were performed using GraphPad Prism. The two-tailed Student's t-test was used for two-group comparisons, and one-way ANOVA with Tukey test was applied for multiple group comparisons. $P < 0.05$, $P < 0.01$, and $P < 0.001$ were considered statistically significant; ns was presented as not significant.

^{1}H NMR and ^{13}C NMR Spectra

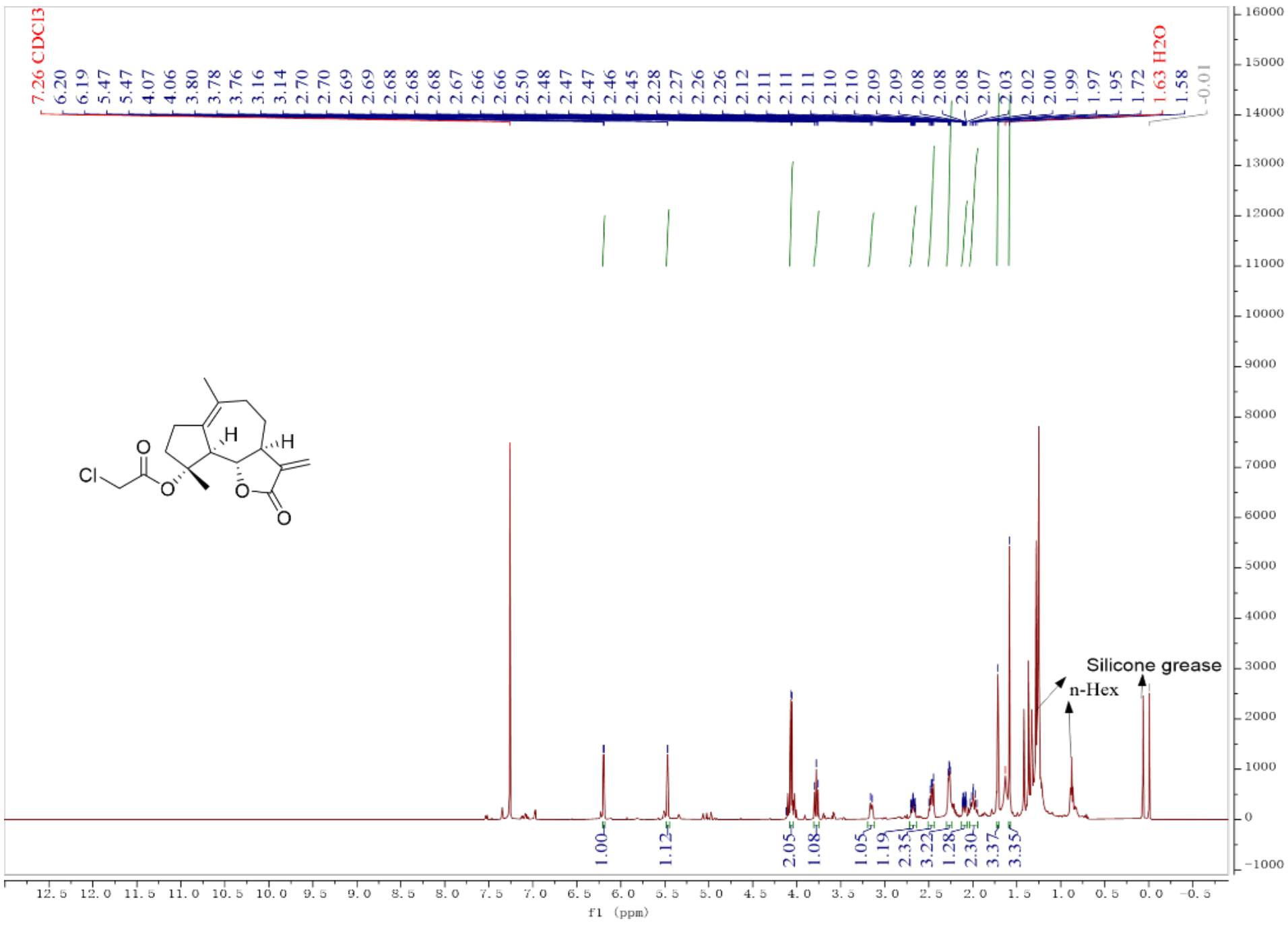

Figure S1. ^{1}H NMR spectrum of **a** (Chloroform-*d*).

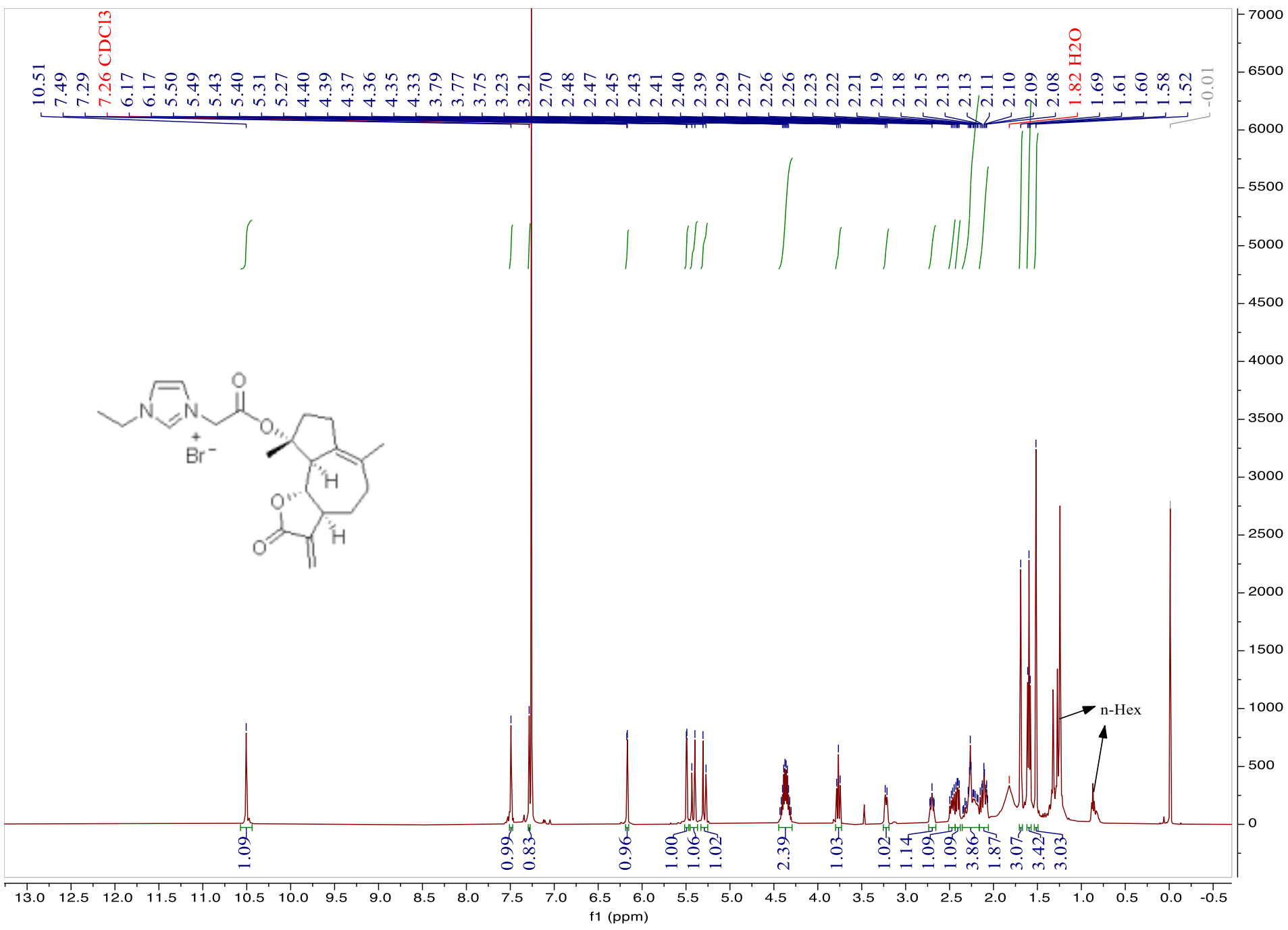

Figure S2. ^{1}H NMR spectrum of **c1** (Chloroform-*d*).

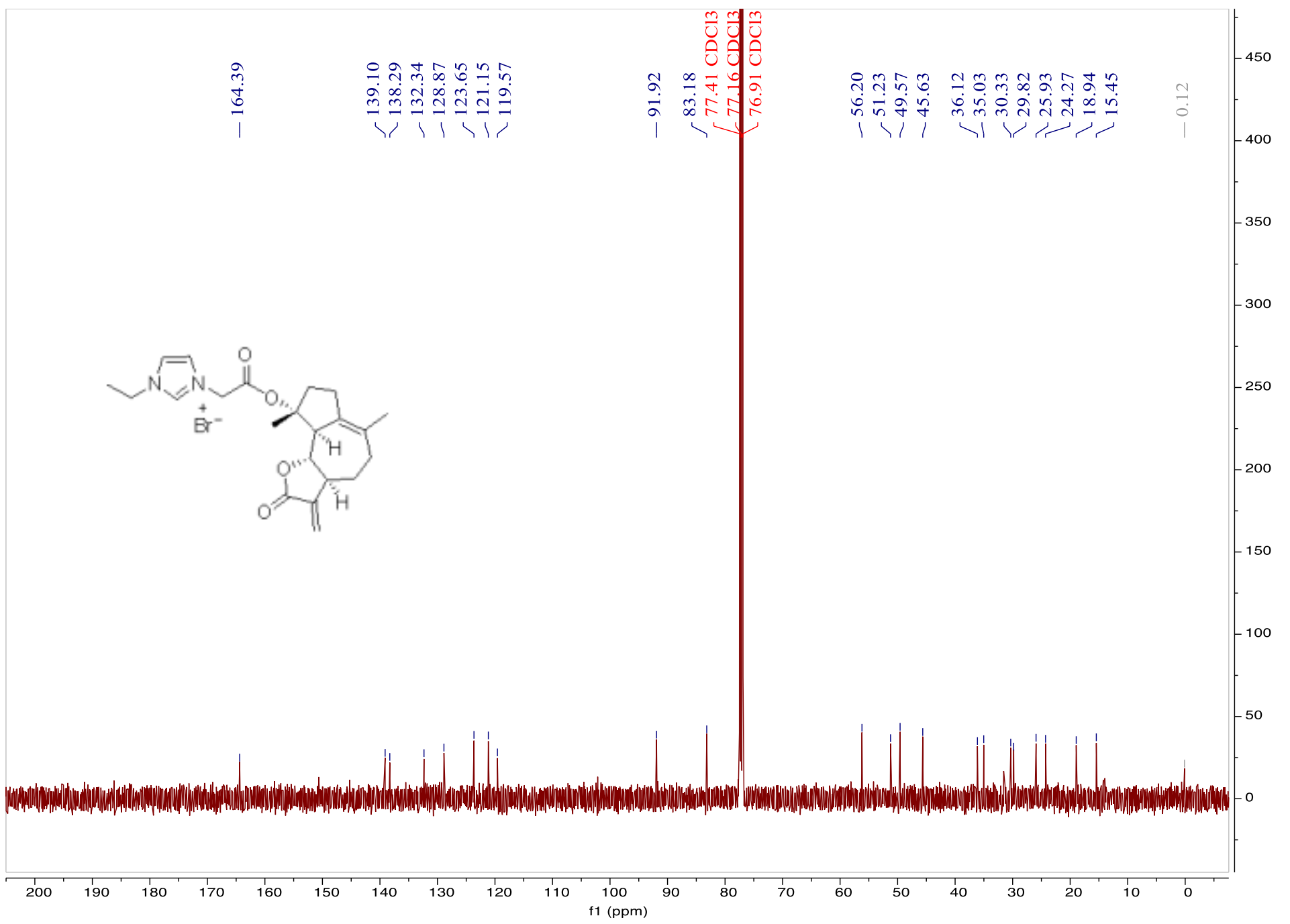

Figure S3. ^{13}C NMR spectrum of **c1** (Chloroform-*d*).

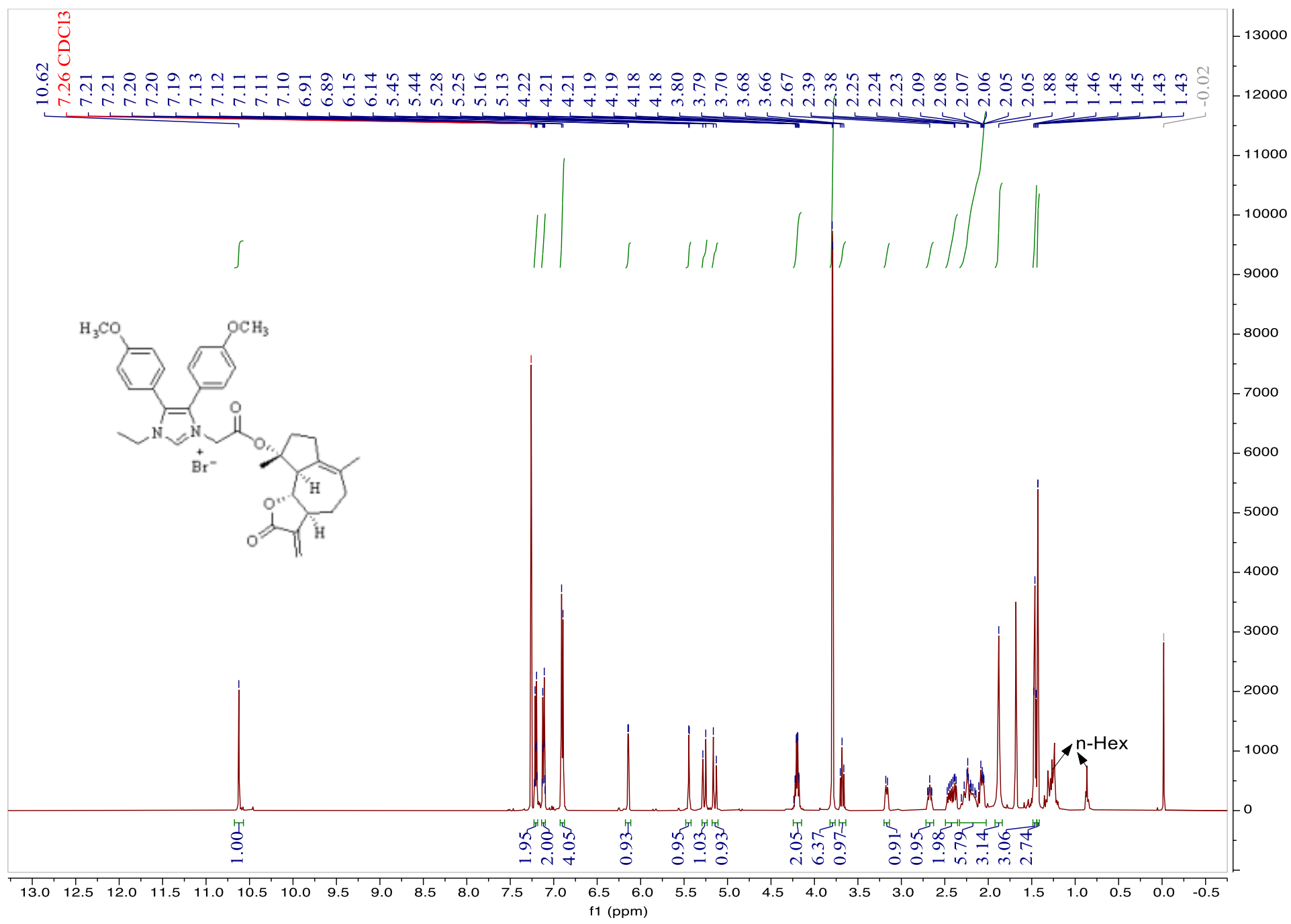

Figure S4. ^{1}H NMR spectrum of **c2** (Chloroform-*d*).

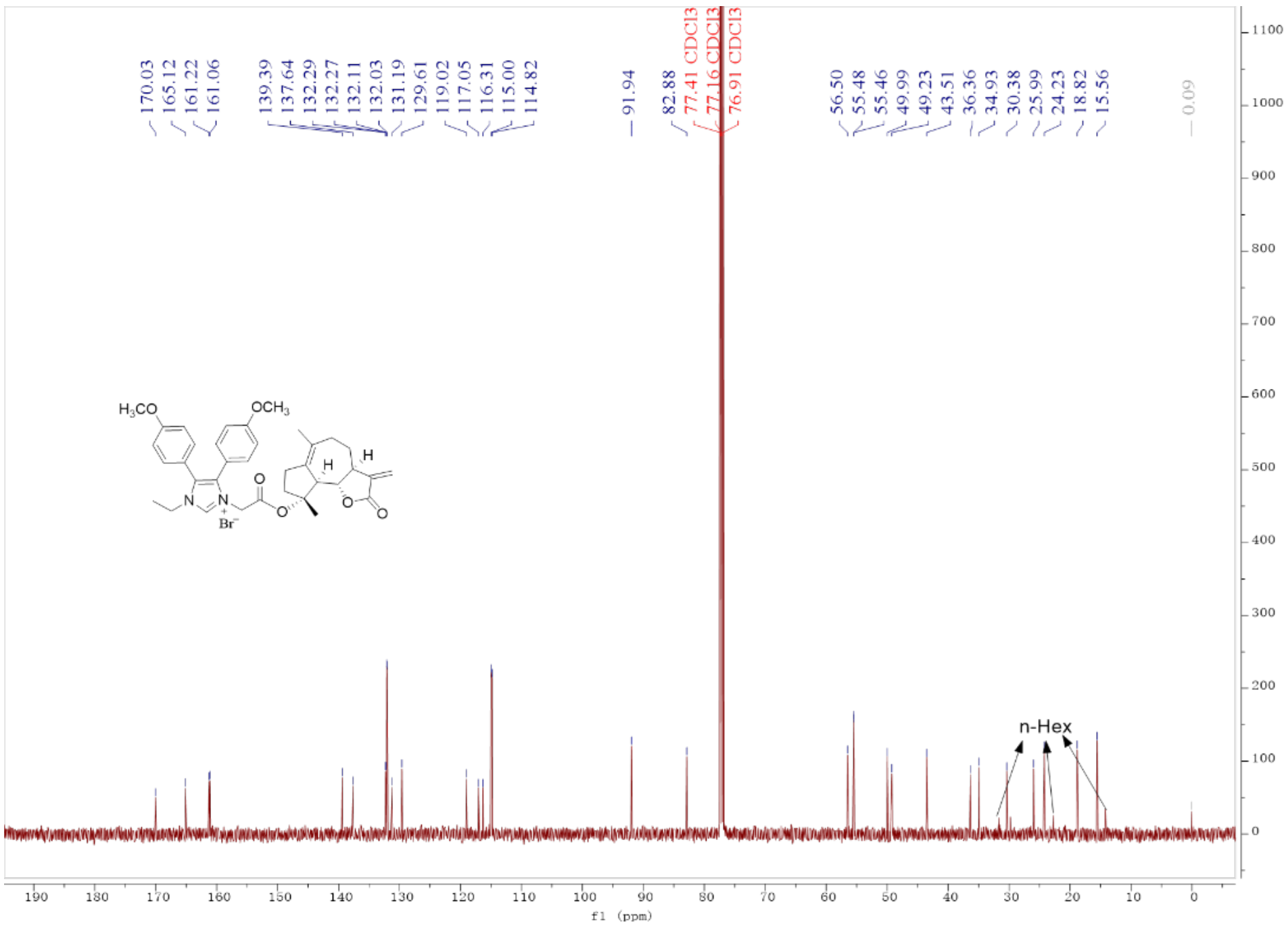

Figure S5. ^{13}C NMR spectrum of **c2** (Chloroform-*d*).

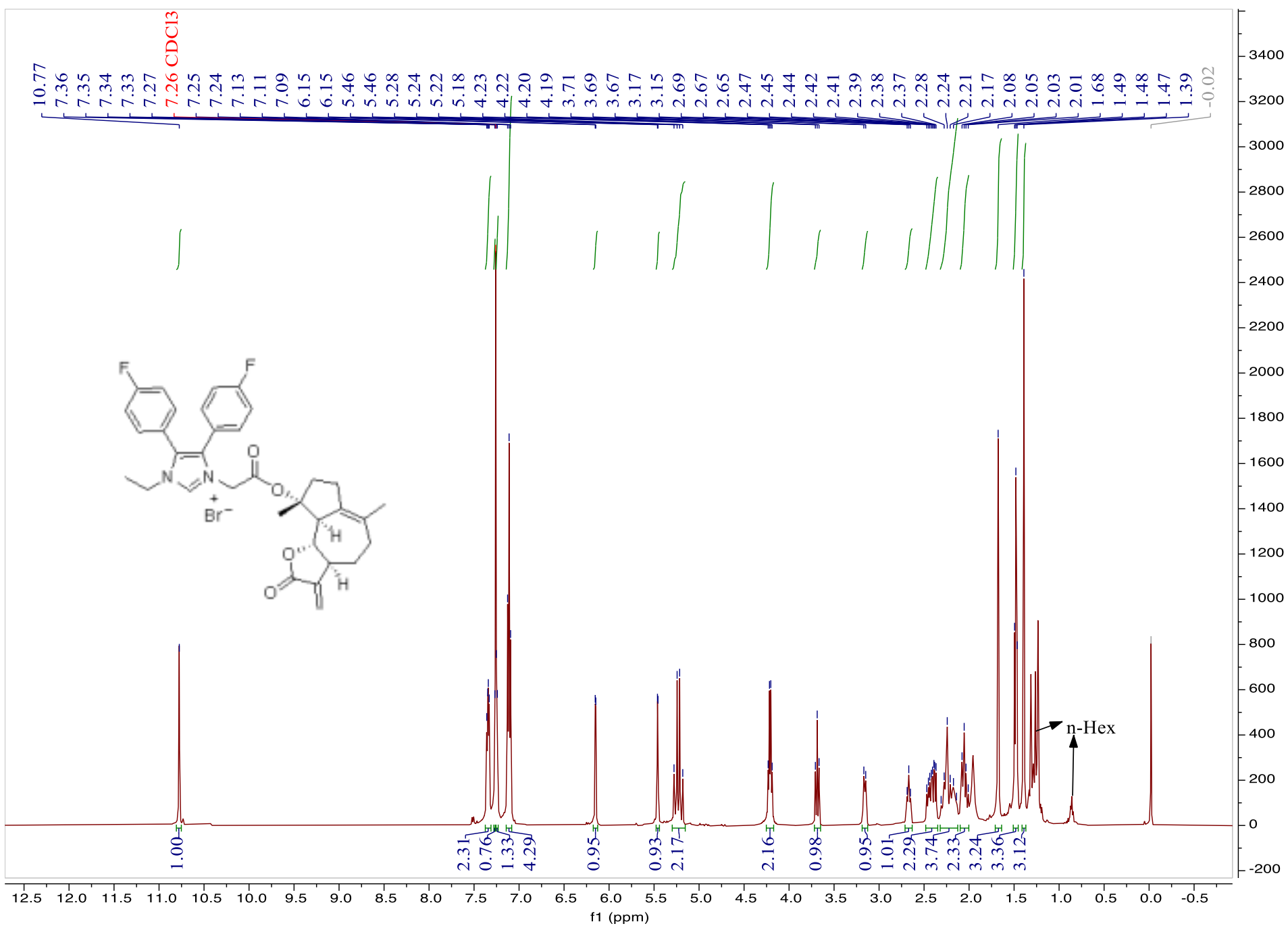

Figure S6. ^{1}H NMR spectrum of **c3** (Chloroform-*d*).

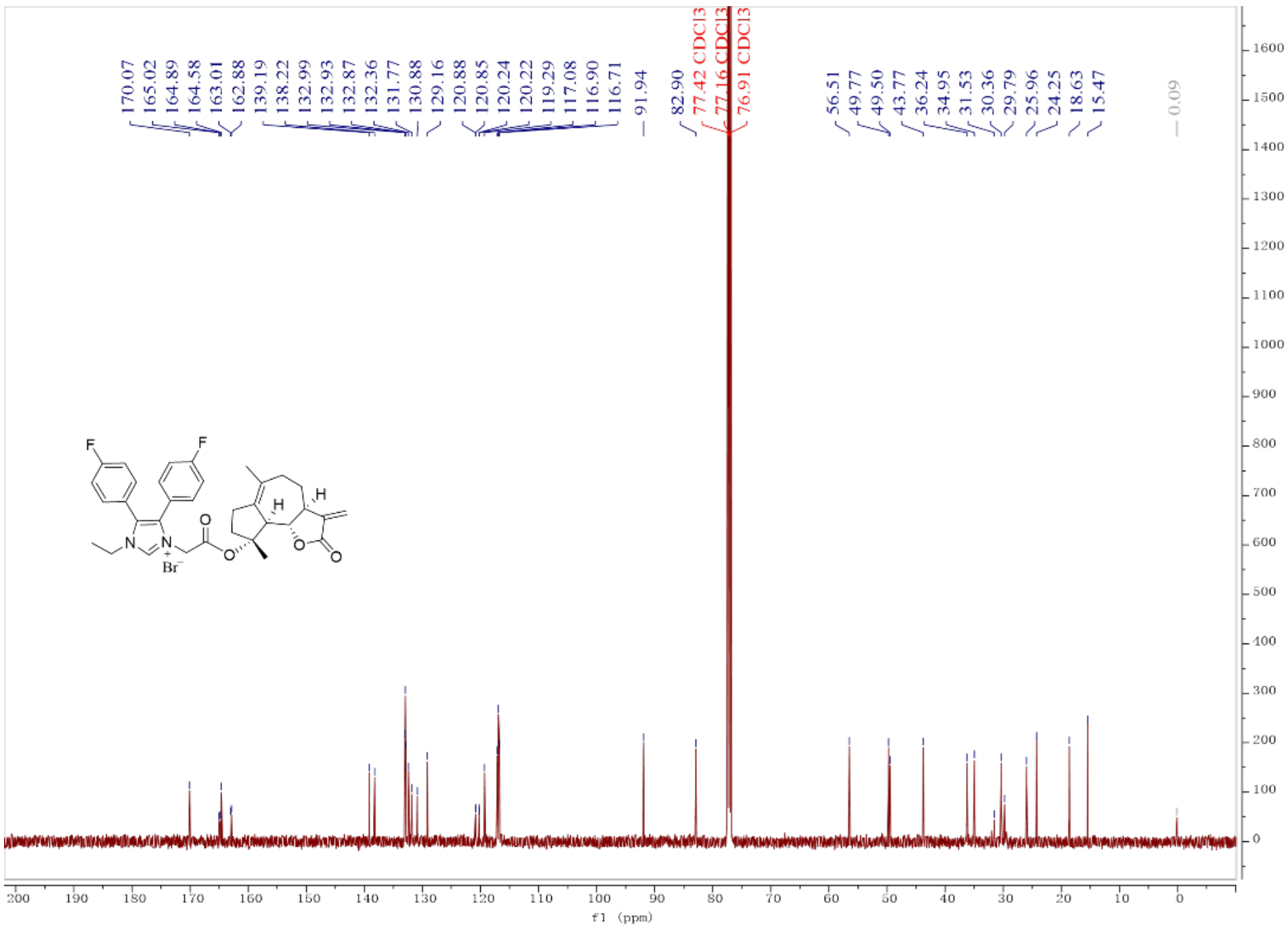

Figure S7. ^{13}C NMR spectrum of **c3** (Chloroform-*d*).

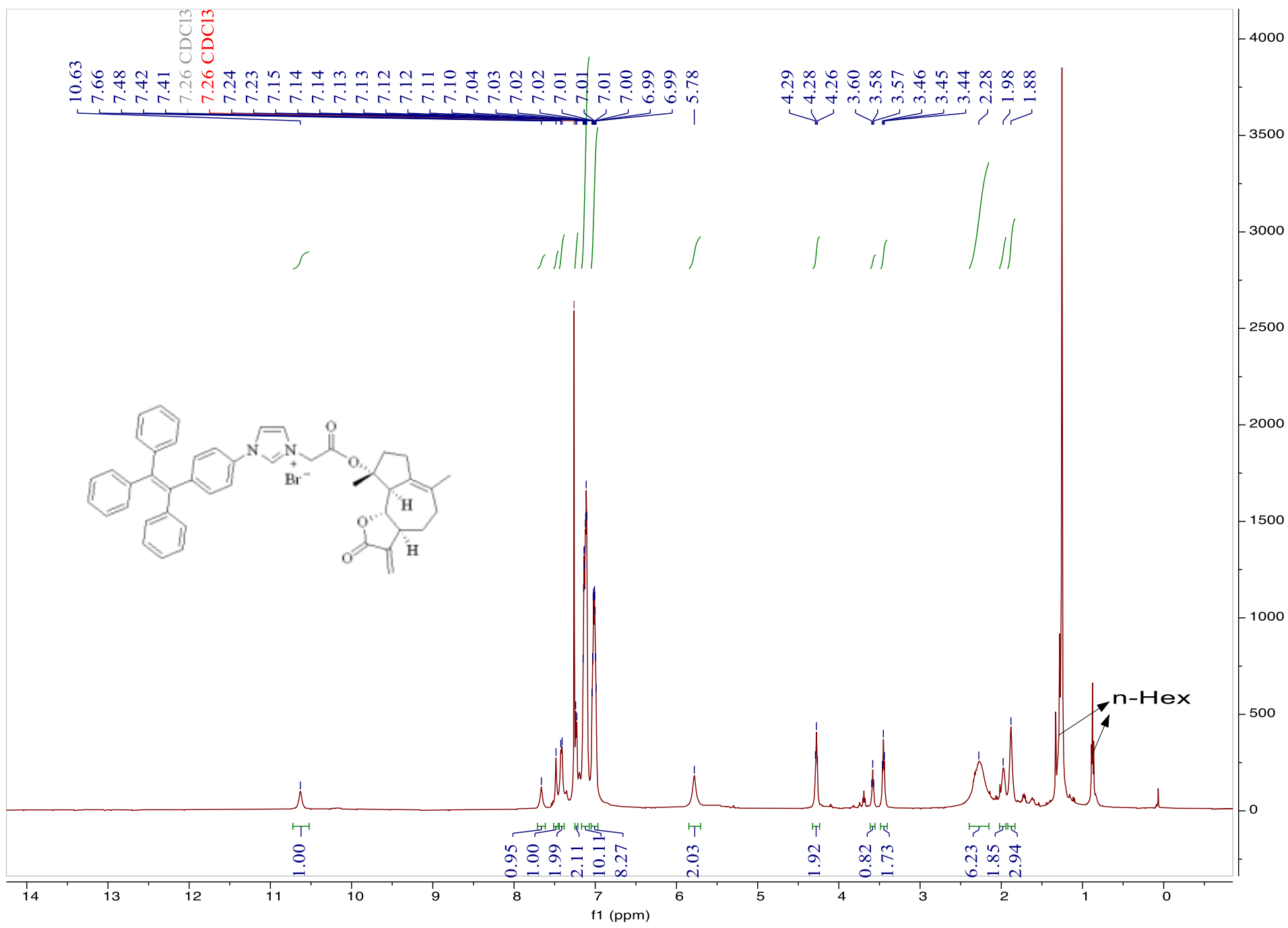

Figure S8. ^{1}H NMR spectrum of **h** (Chloroform-*d*).

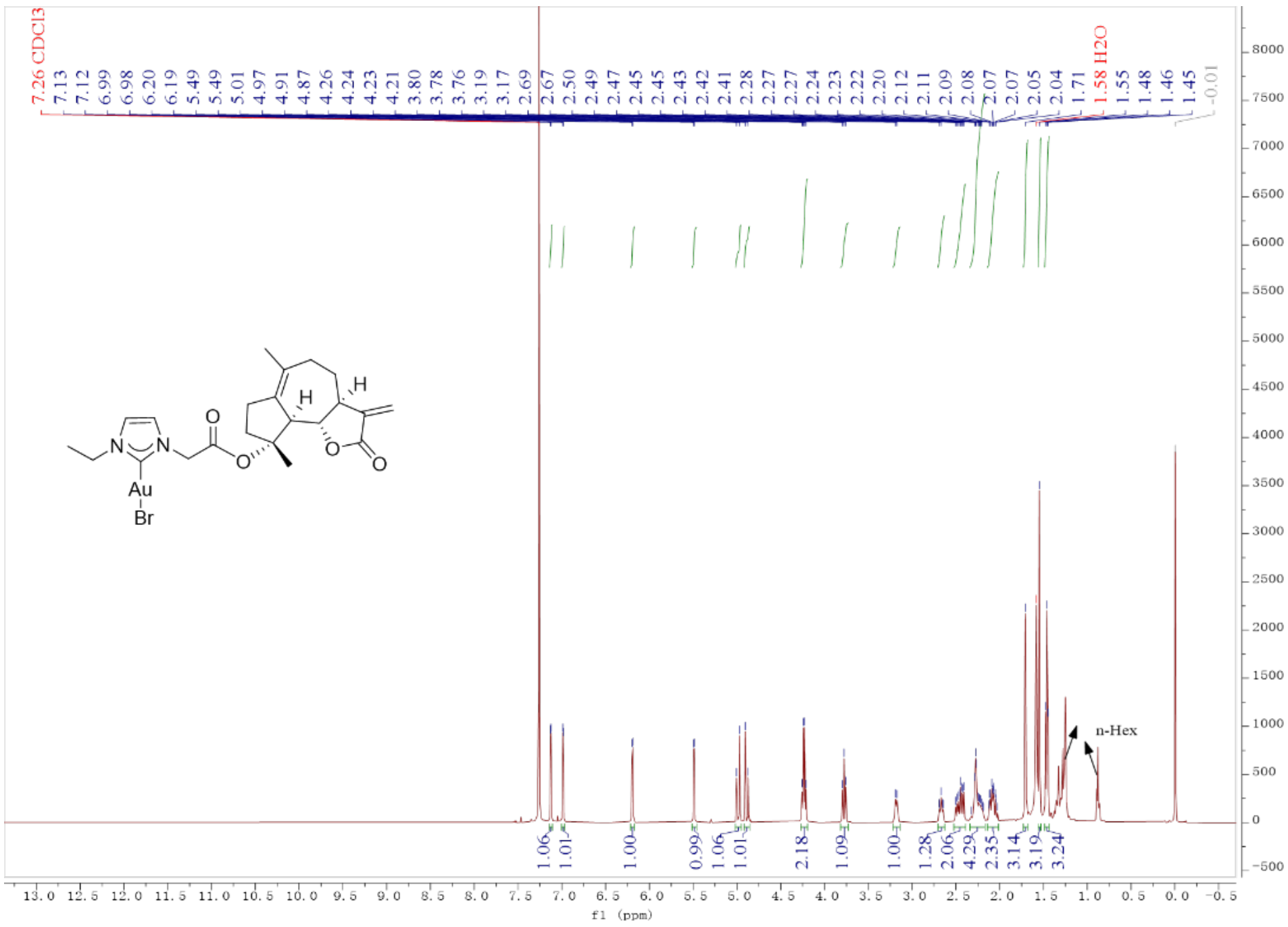

Figure S9. ^{1}H NMR spectrum of **Au1** (Chloroform-*d*).

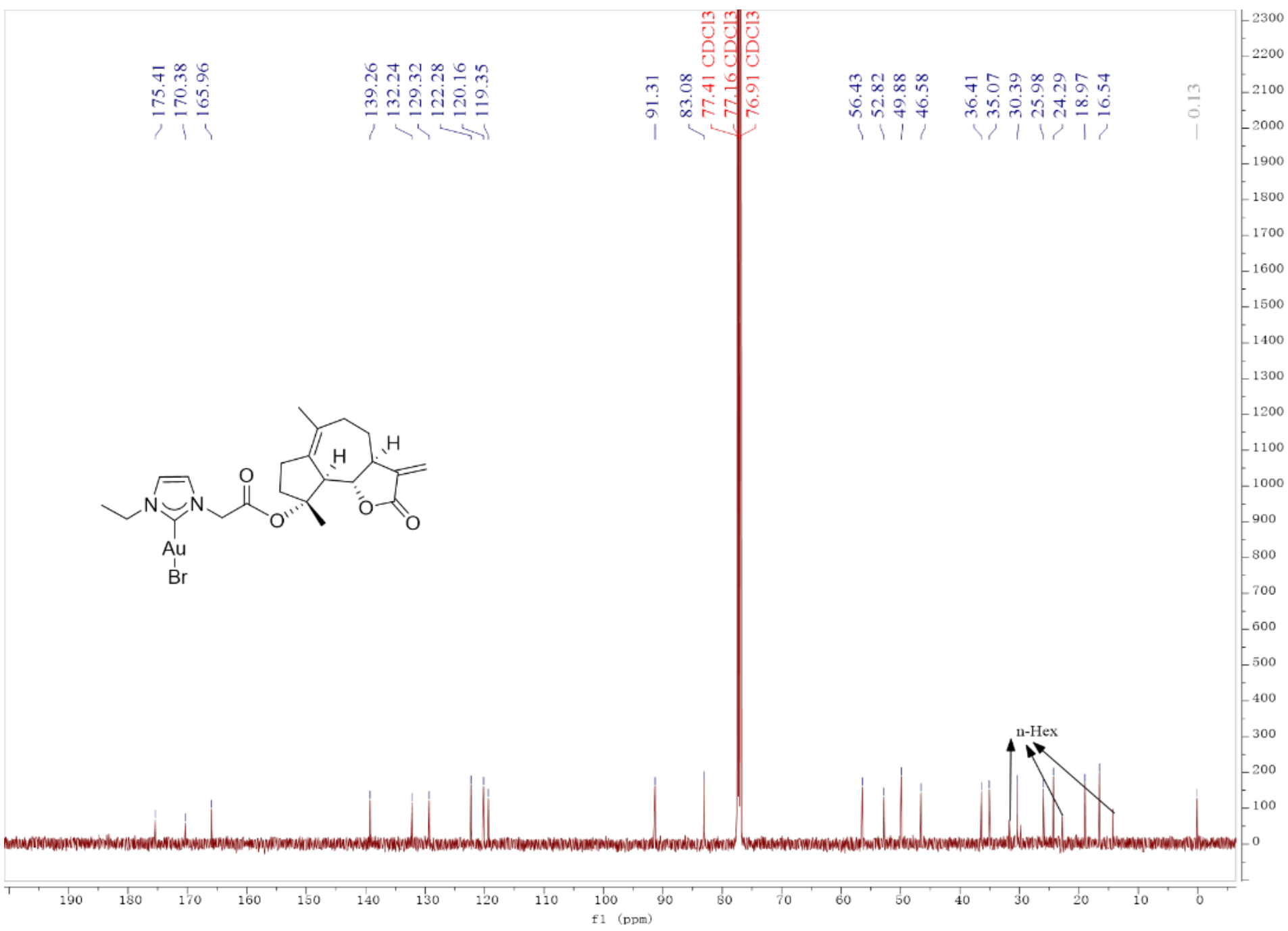

Figure S10. ^{13}C NMR spectrum of **Au1** (Chloroform-*d*).

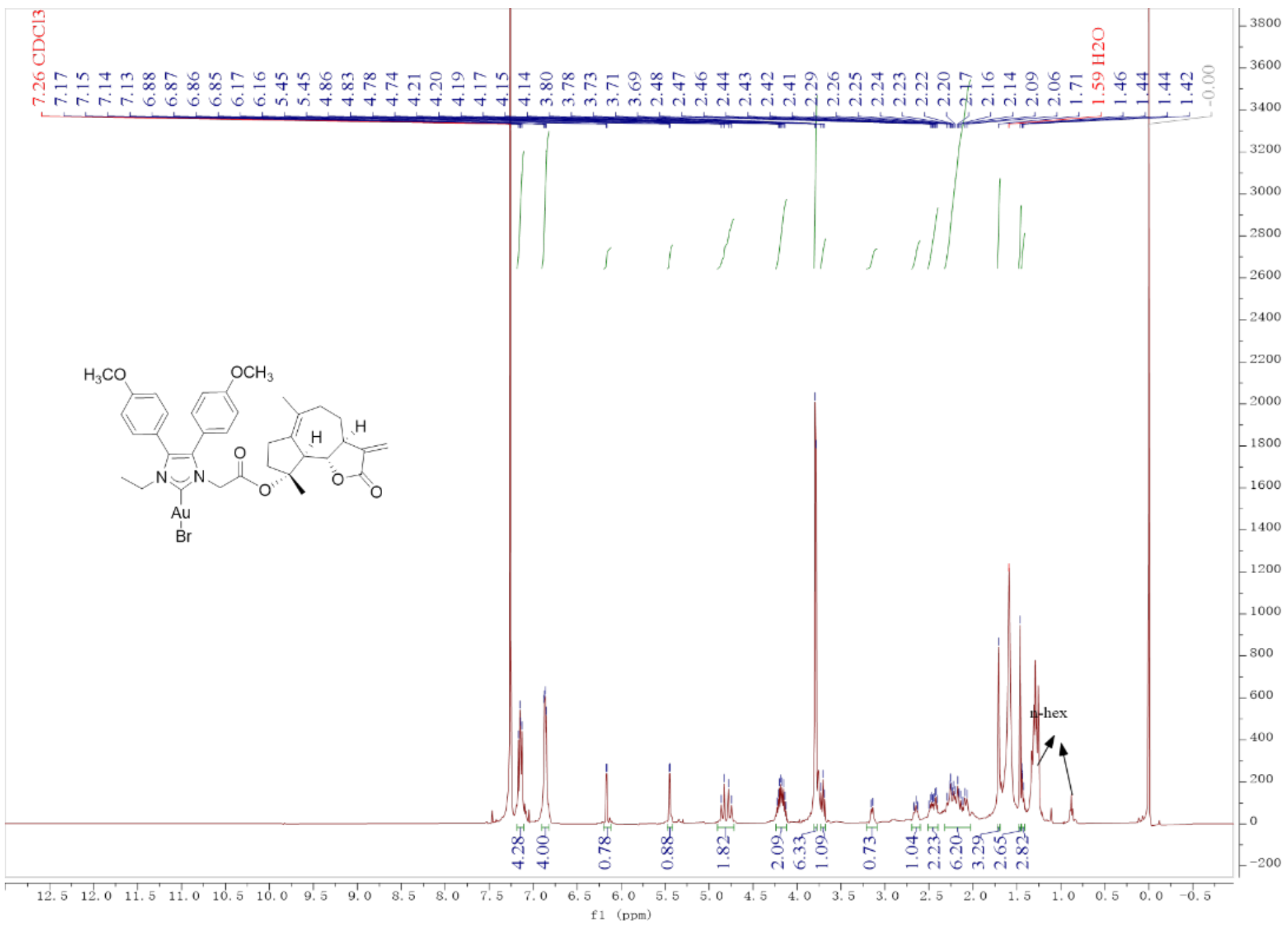

Figure S11. ^{1}H NMR spectrum of **Au2** (Chloroform-*d*).

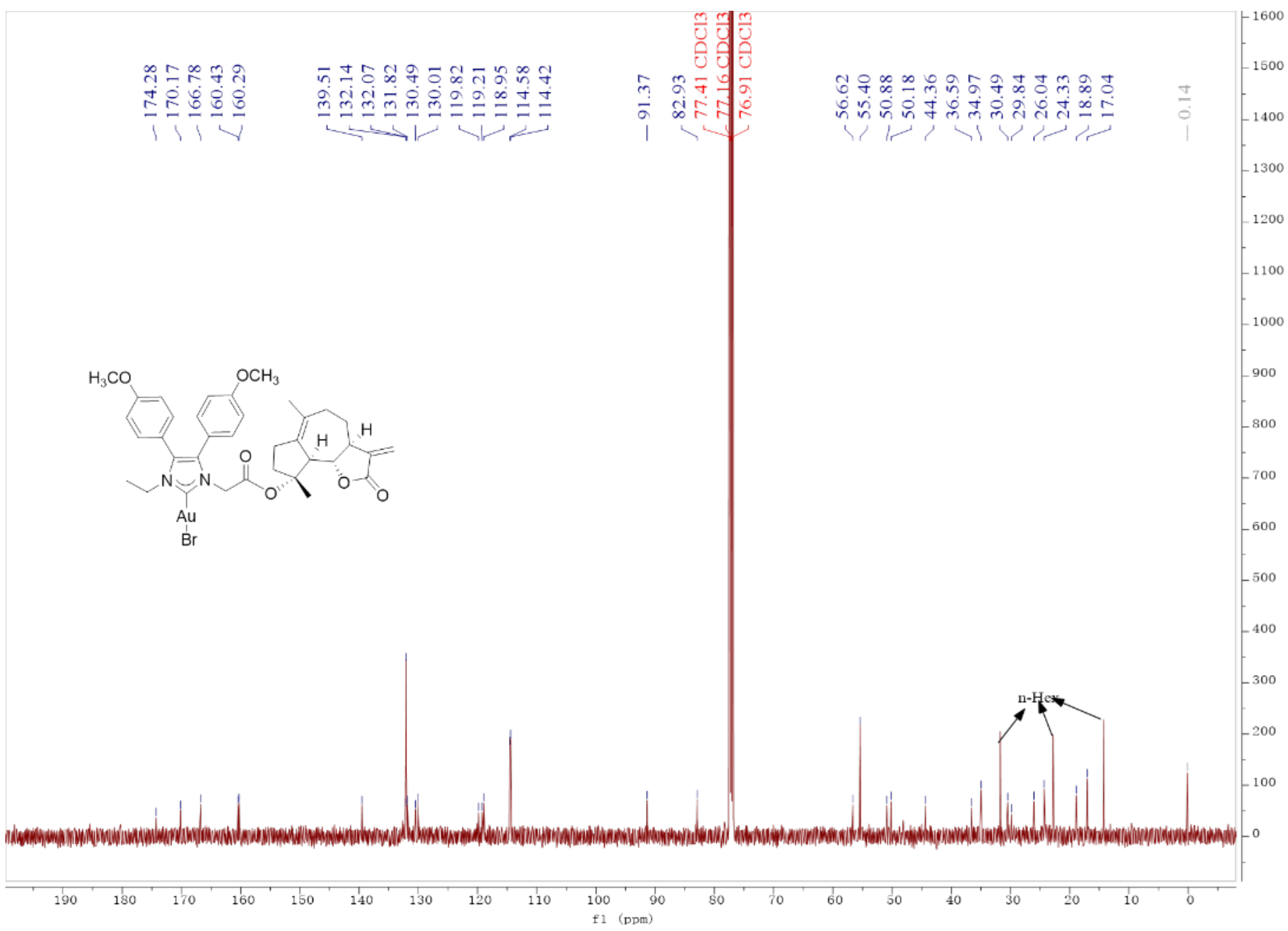

Figure S12. ^{13}C NMR spectrum of **Au2** (Chloroform-*d*).

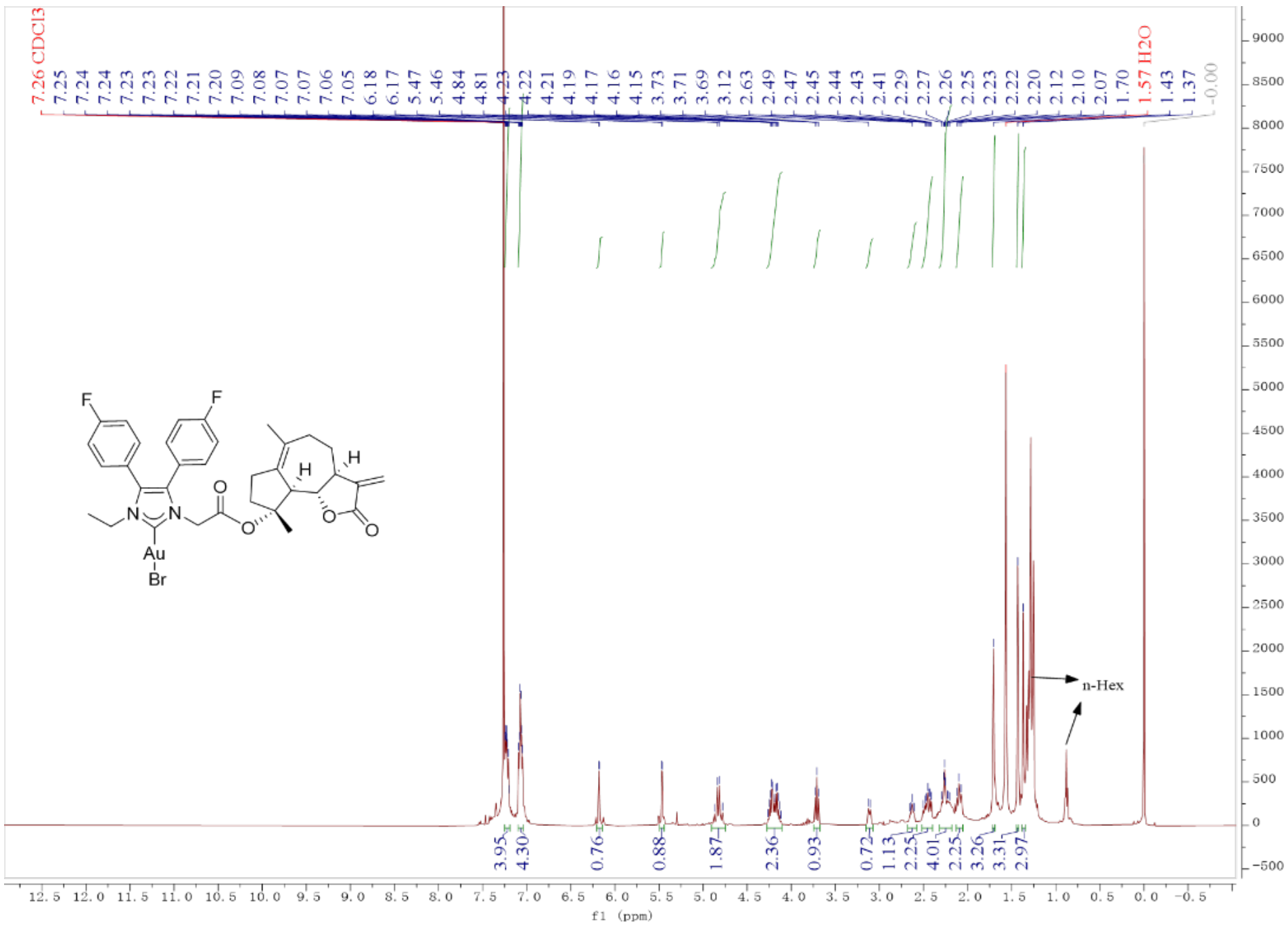

Figure S13. ^{1}H NMR spectrum of **Au3** (Chloroform-*d*).

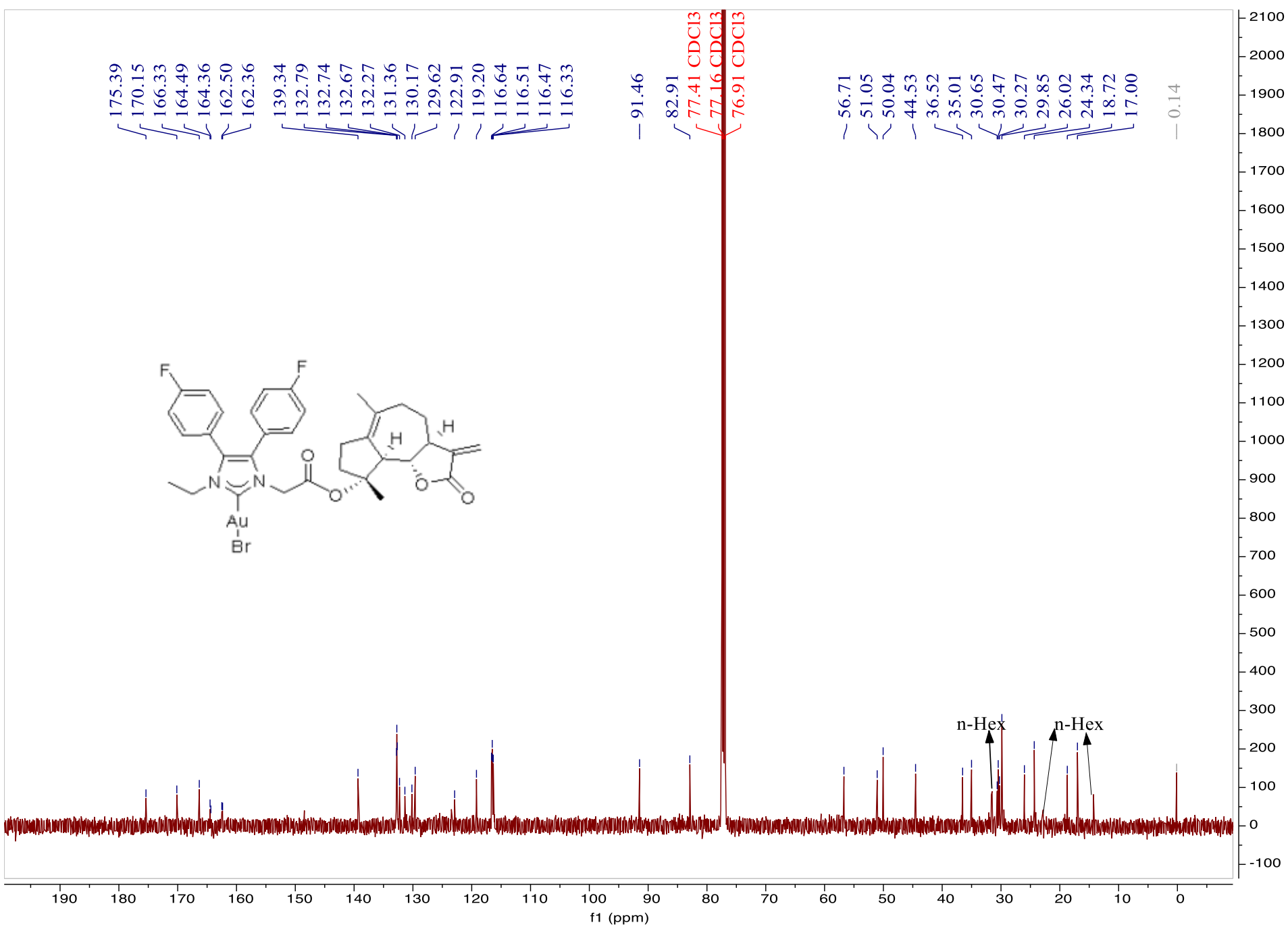

Figure S14. ^{13}C NMR spectrum of **Au3** (Chloroform-*d*).

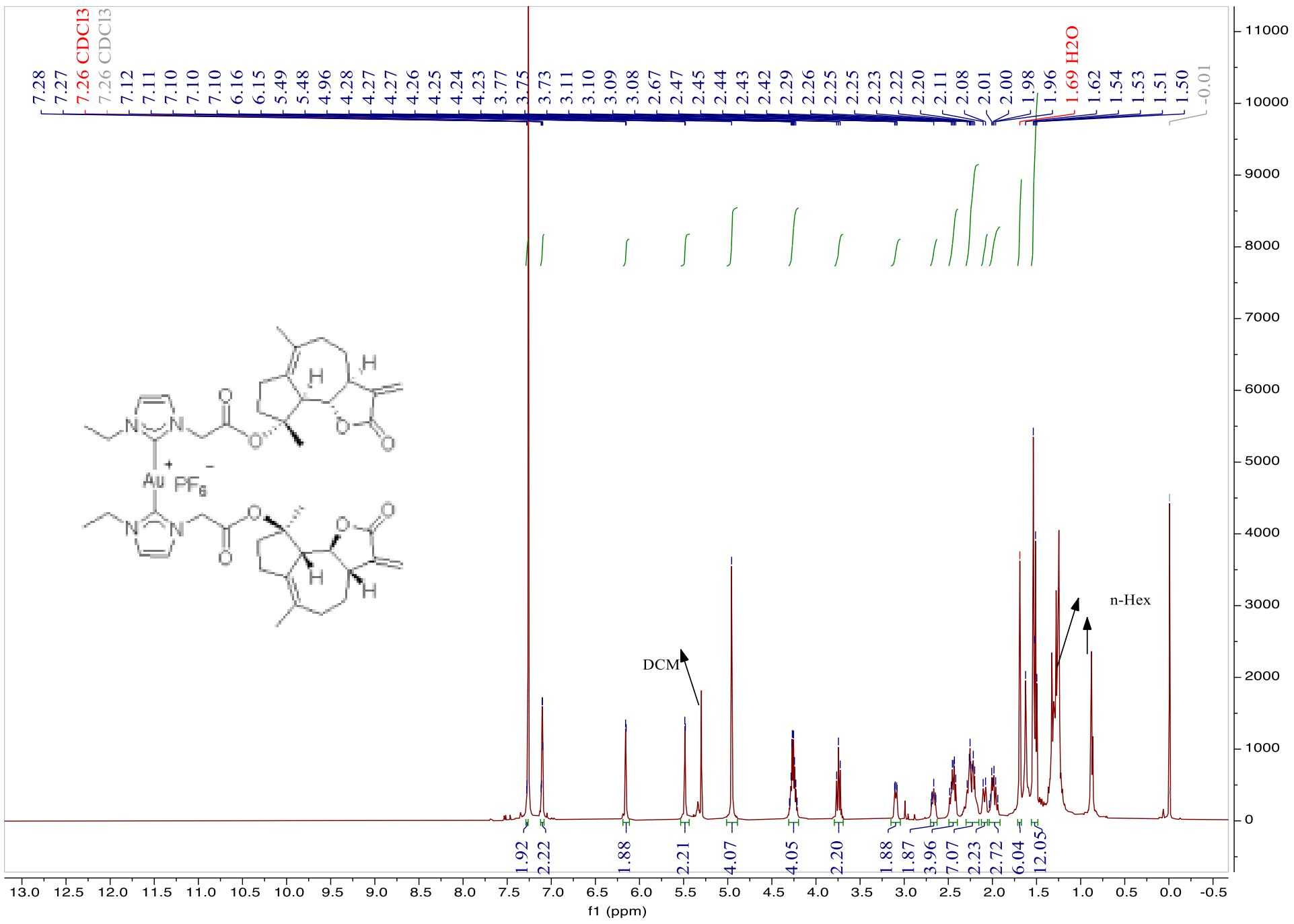

Figure S15. ^{1}H NMR spectrum of **Au4** (Chloroform-*d*).

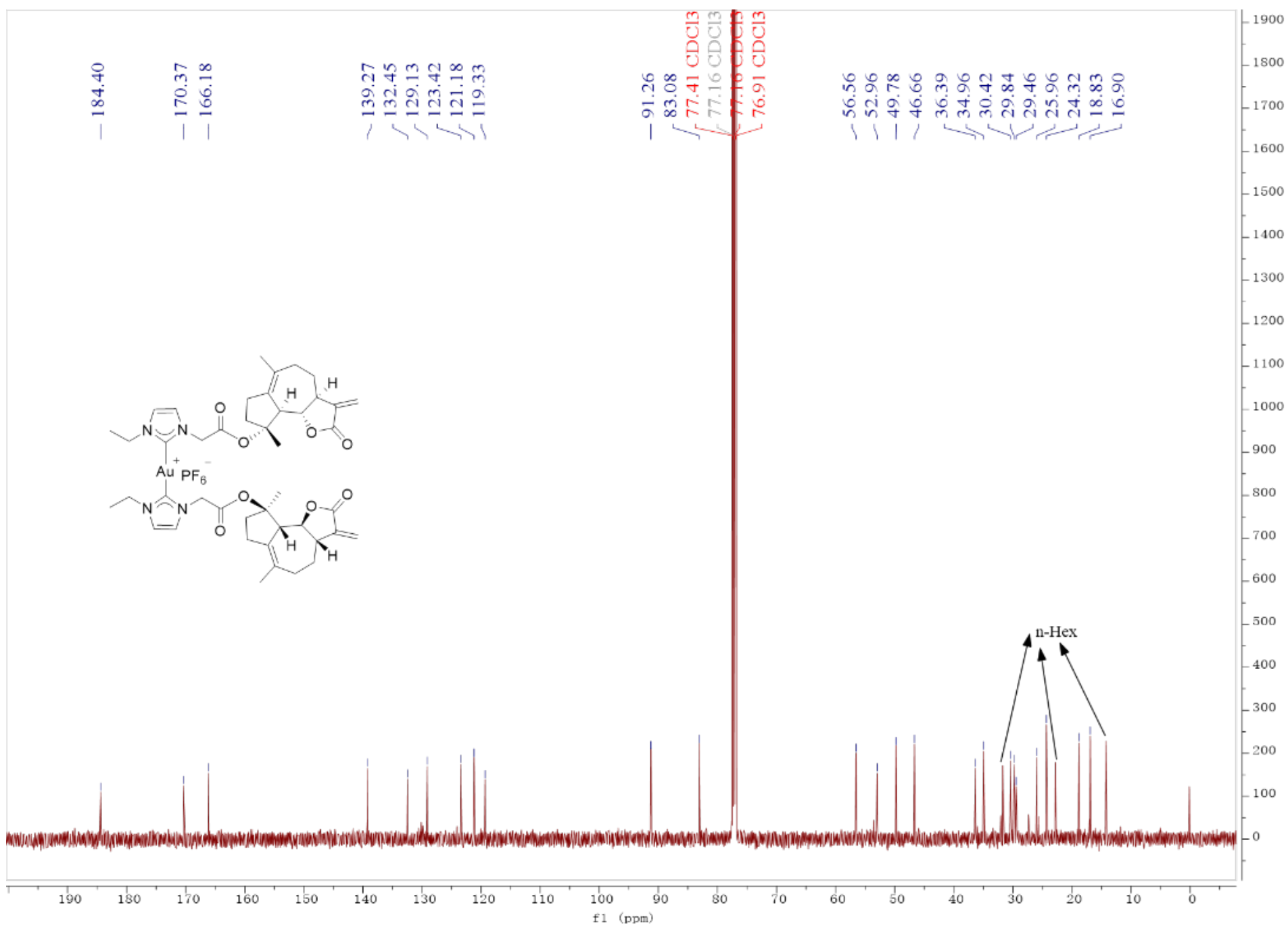

Figure S16. ^{13}C NMR spectrum of **Au4** (Chloroform-*d*).

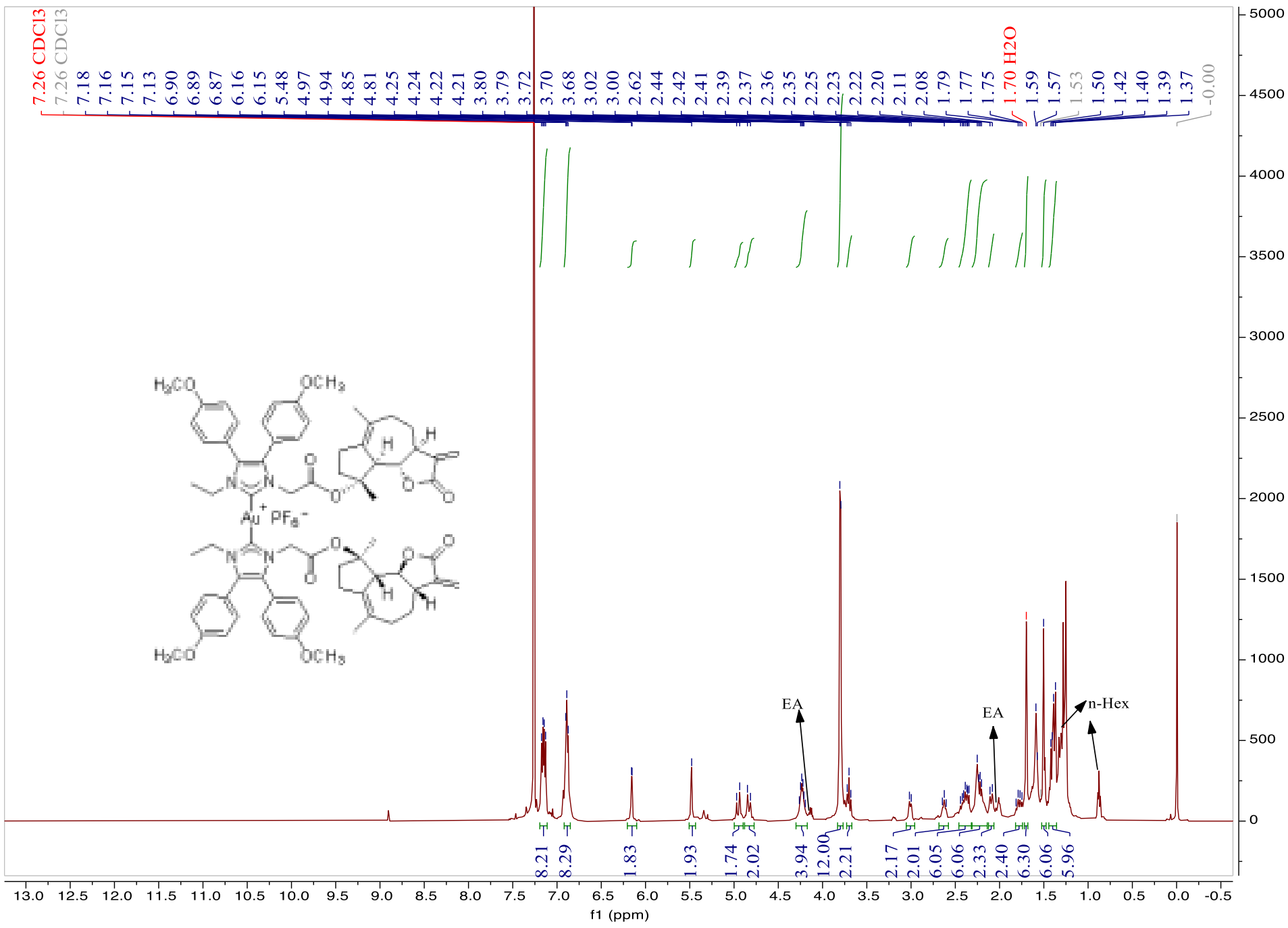

Figure S17. ^{1}H NMR spectrum of **Au5** (Chloroform-*d*).

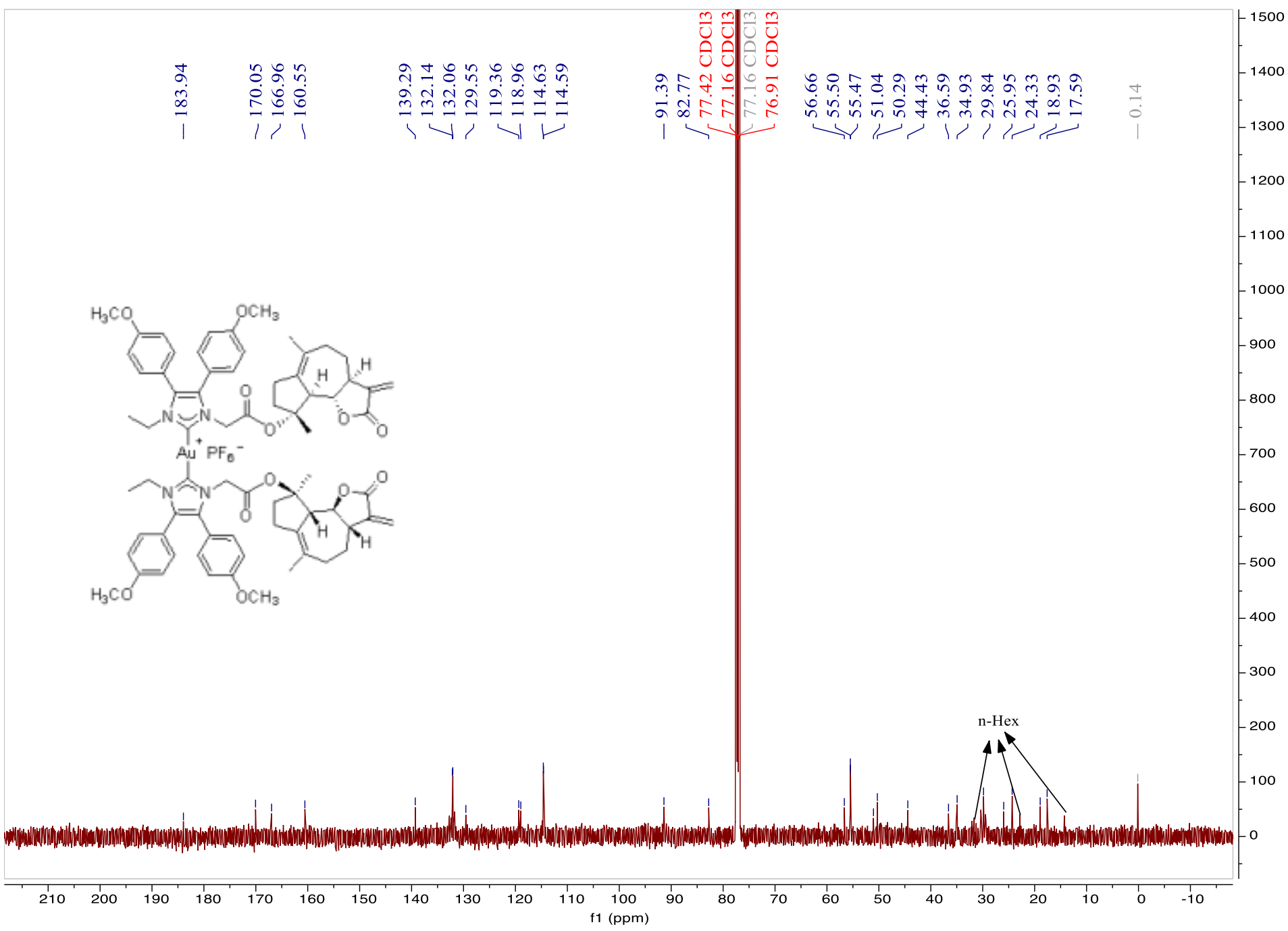

Figure S18. ^{13}C NMR spectrum of **Au5** (Chloroform-*d*).

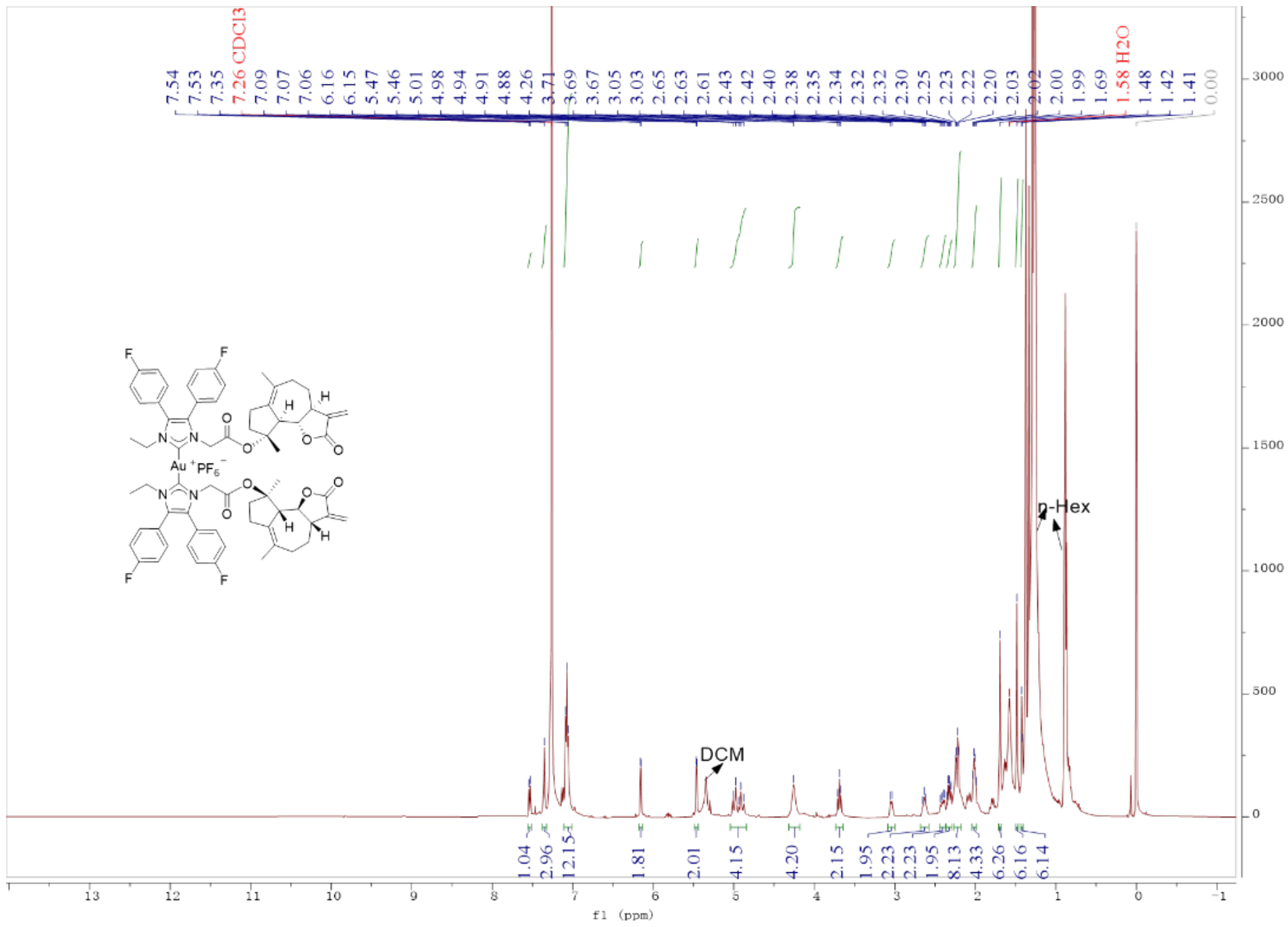

Figure S19. ^{1}H NMR spectrum of **Au6** (Chloroform-*d*).

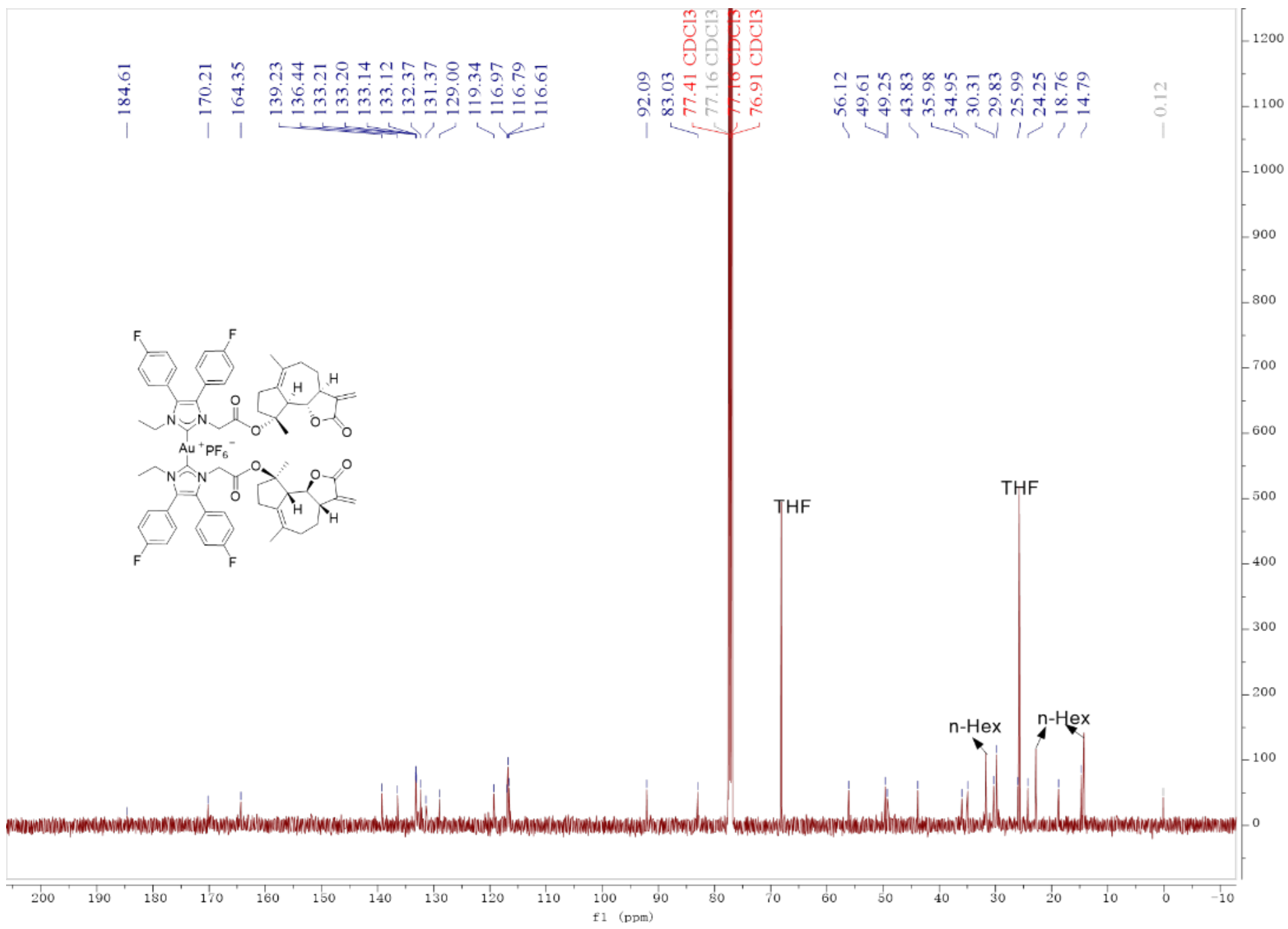

Figure S20. ^{13}C NMR spectrum of **Au6** (Chloroform-*d*).

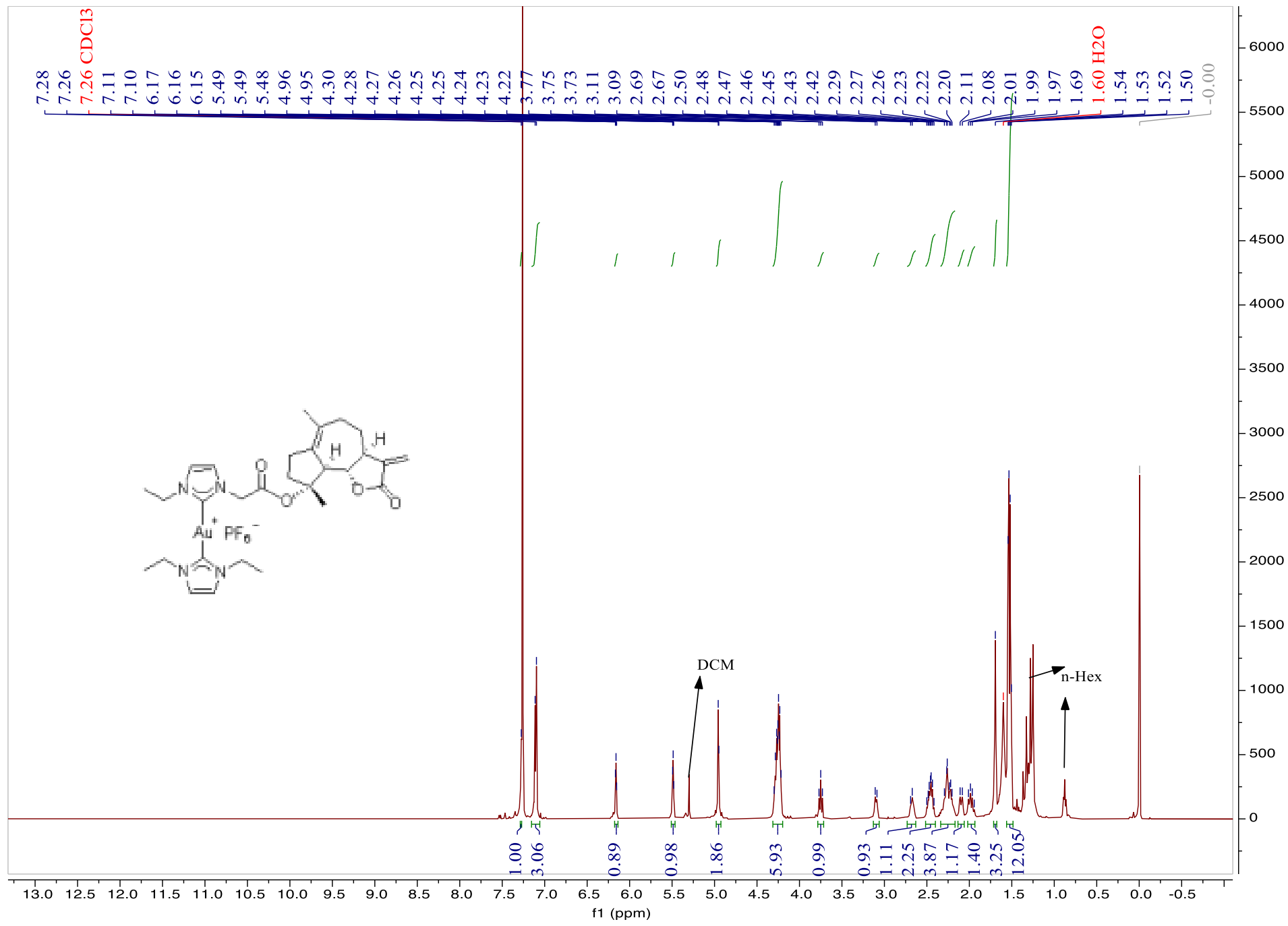

Figure S21. ^{1}H NMR spectrum of **Au7** (Chloroform-*d*).

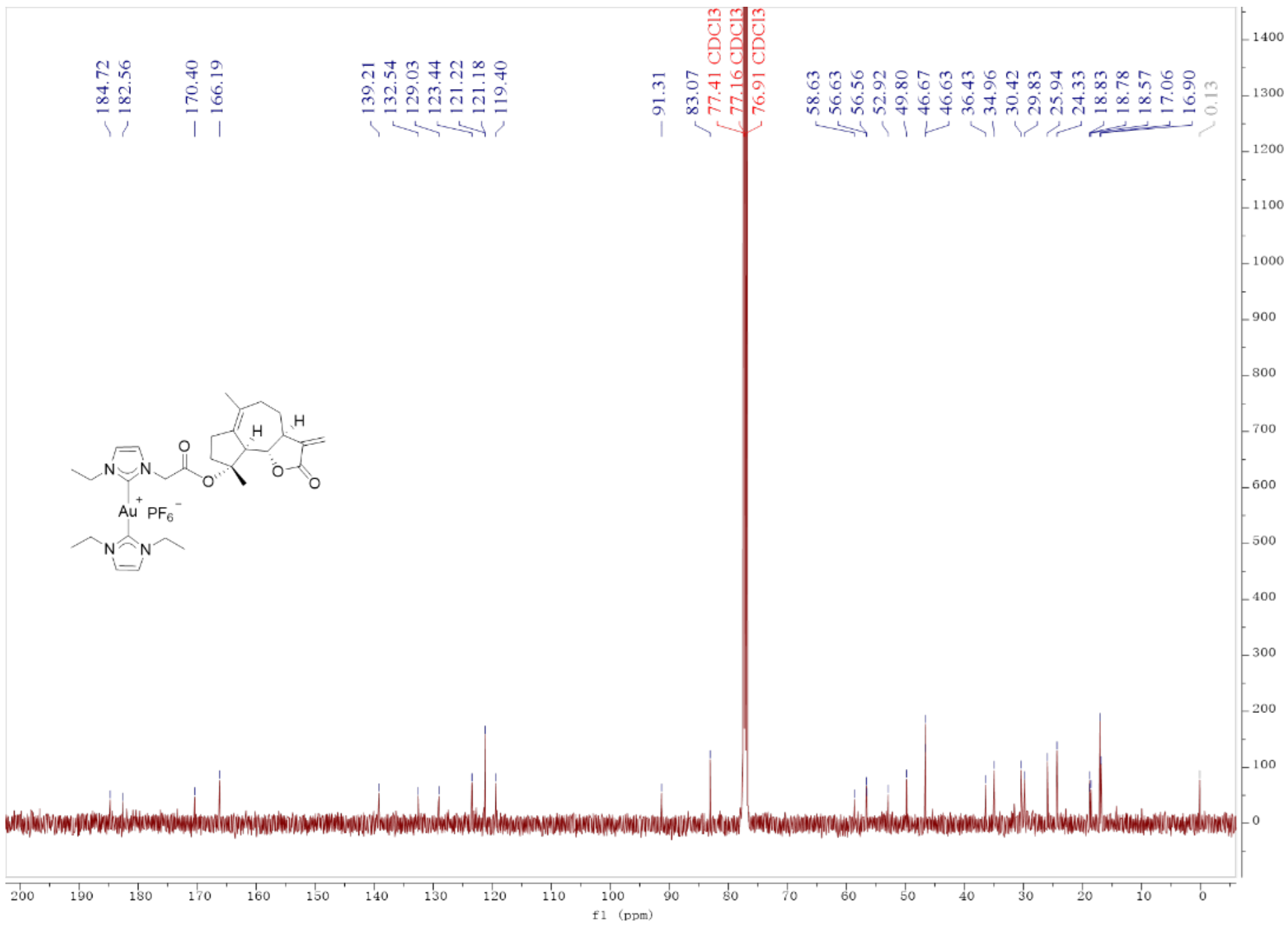

Figure S22. ^{13}C NMR spectrum of **Au7** (Chloroform-*d*).

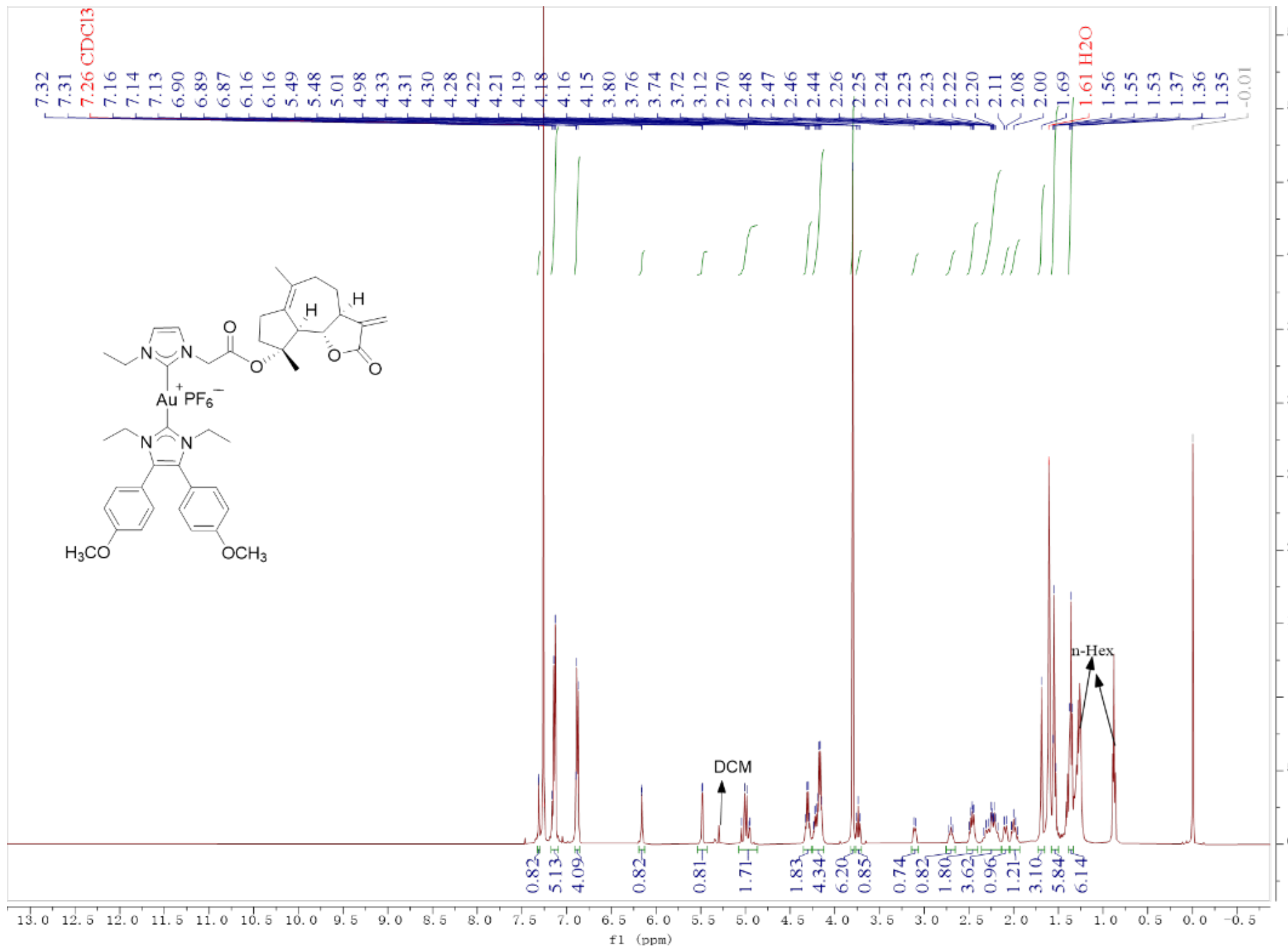

Figure S23. ^{1}H NMR spectrum of **Au8** (Chloroform-*d*).

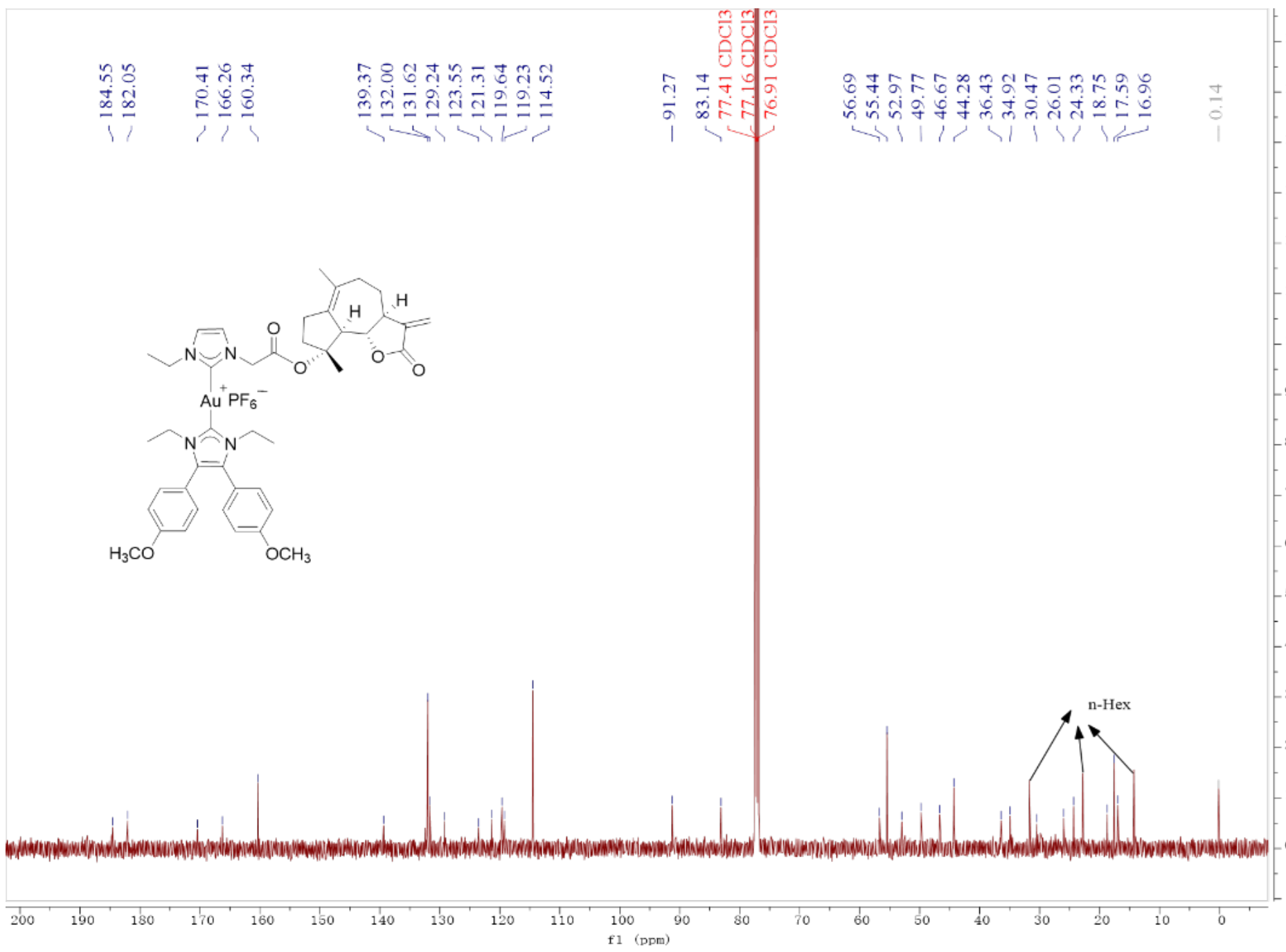

Figure S24. ^{13}C NMR spectrum of **Au8** (Chloroform-*d*).

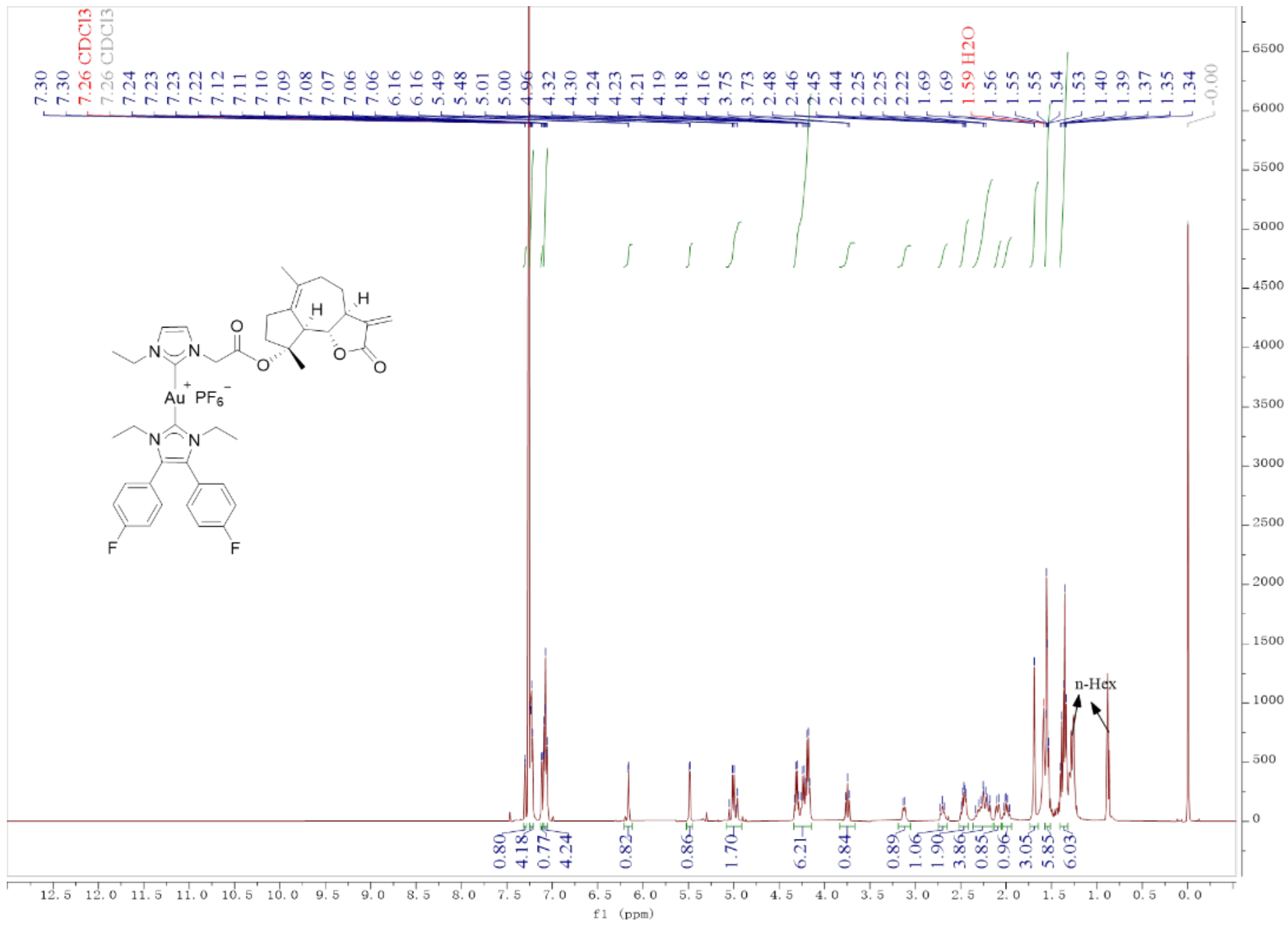

Figure S25. ^{1}H NMR spectrum of **Au9** (Chloroform-*d*).

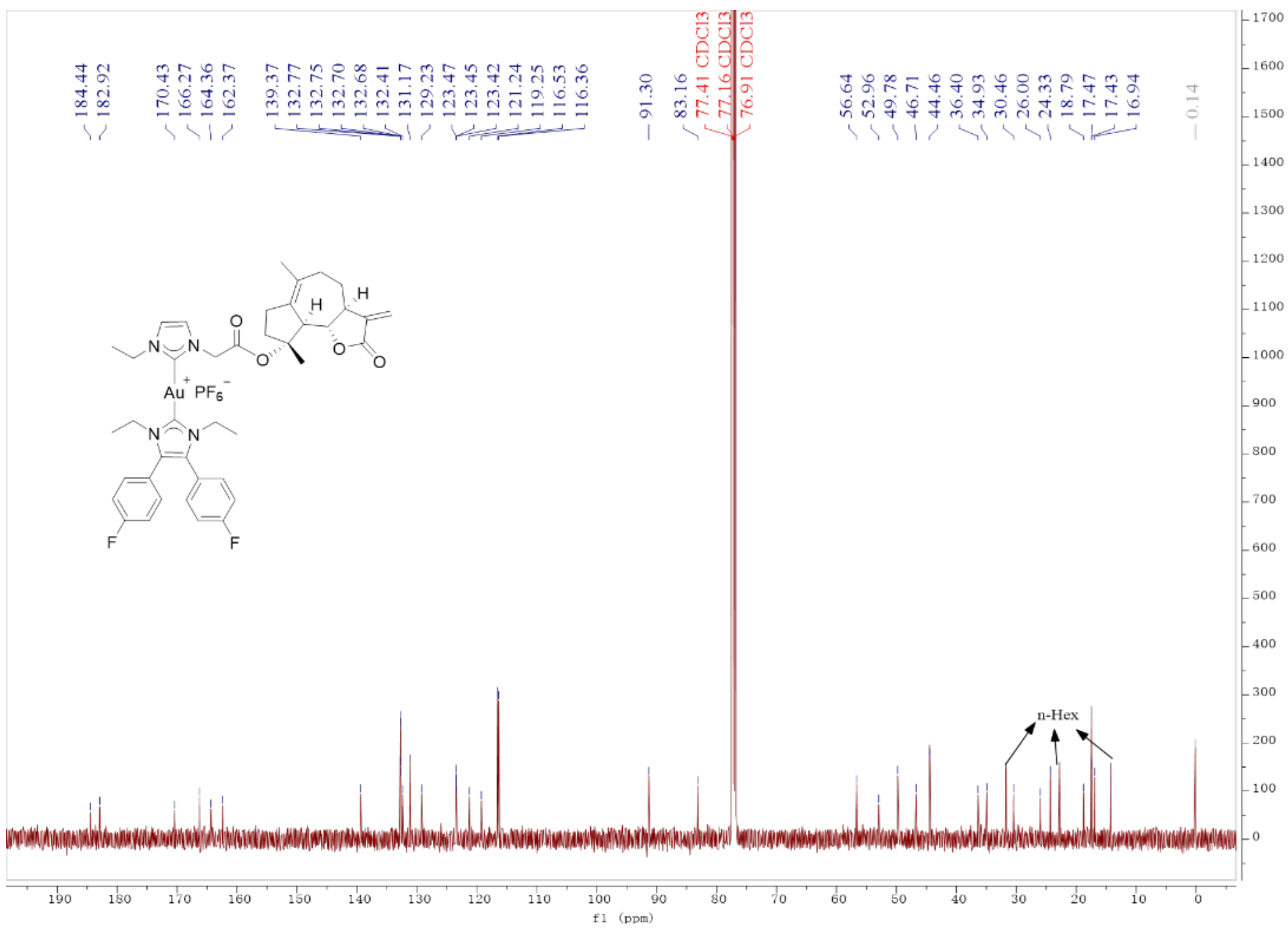

Figure S26. ^{13}C NMR spectrum of **Au9** (Chloroform-*d*).

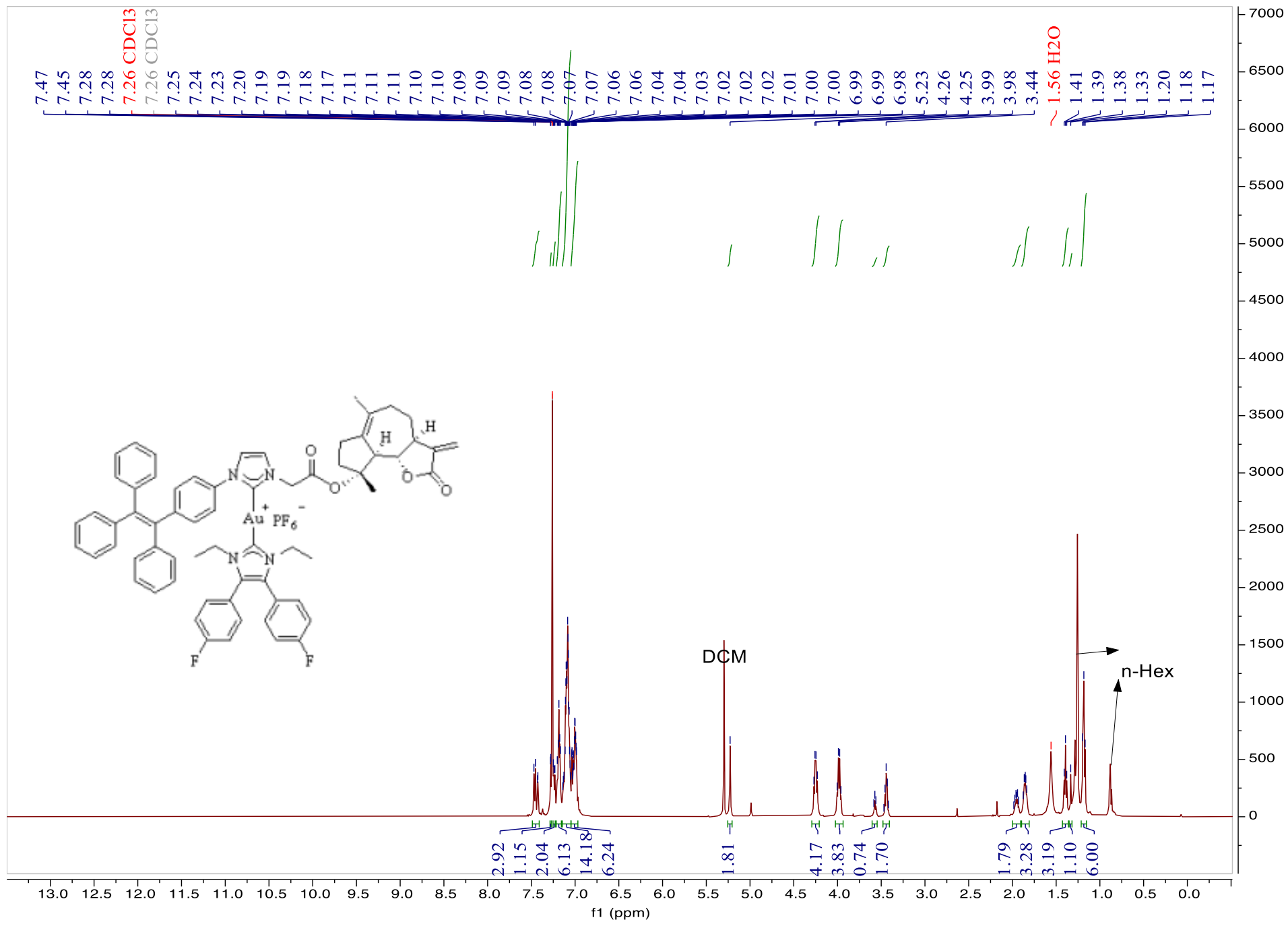

Figure S27. ^{1}H NMR spectrum of **Au10** (Chloroform-*d*).

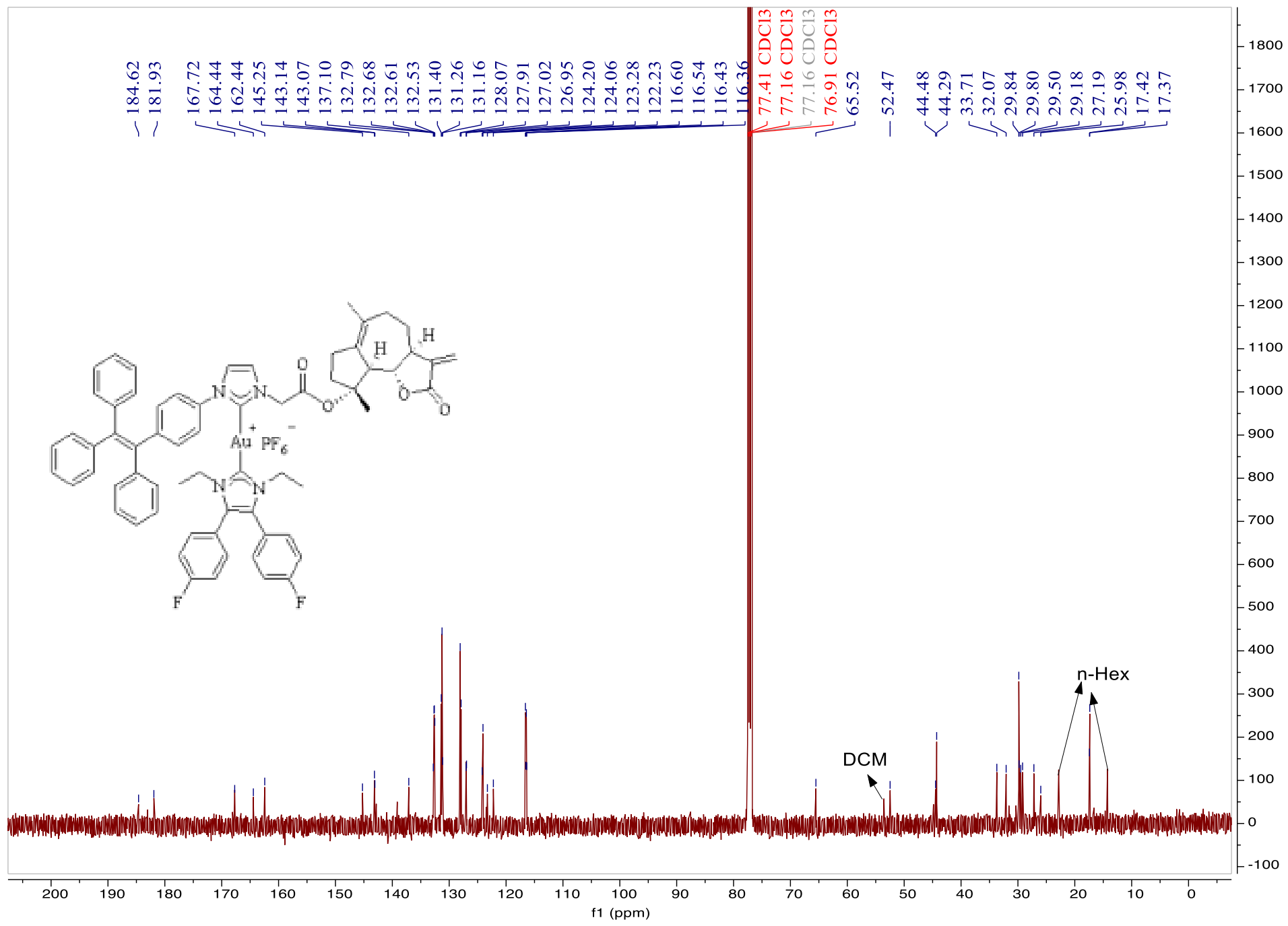

Figure S28. ^{13}C NMR spectrum of **Au10** (Chloroform-*d*).

HPLC Chromatograms

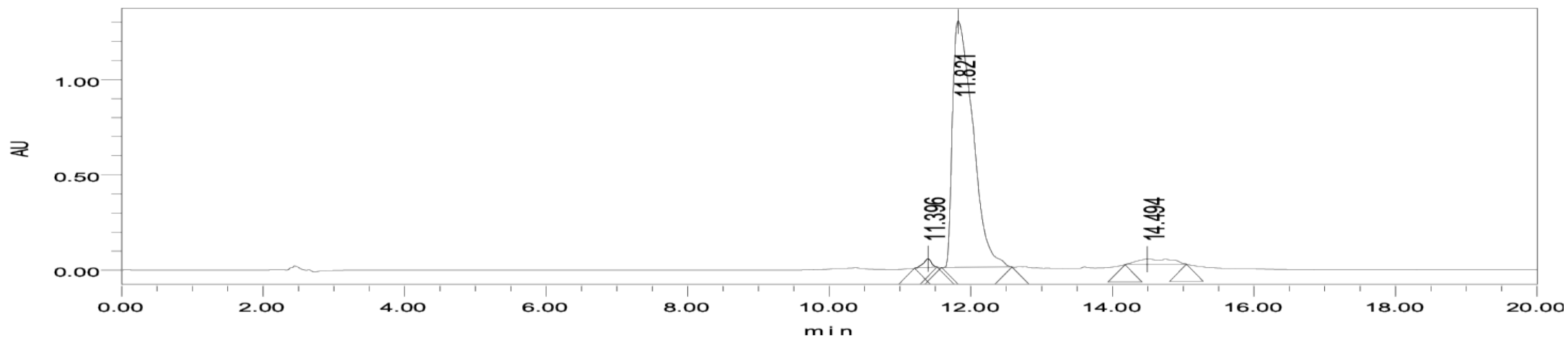

NO.	Rt (min)	Area (%)
1	11.396	1.30
2	11.821	95.14
3	14.494	3.55

Figure S29. HPLC chromatogram of **Au1**.

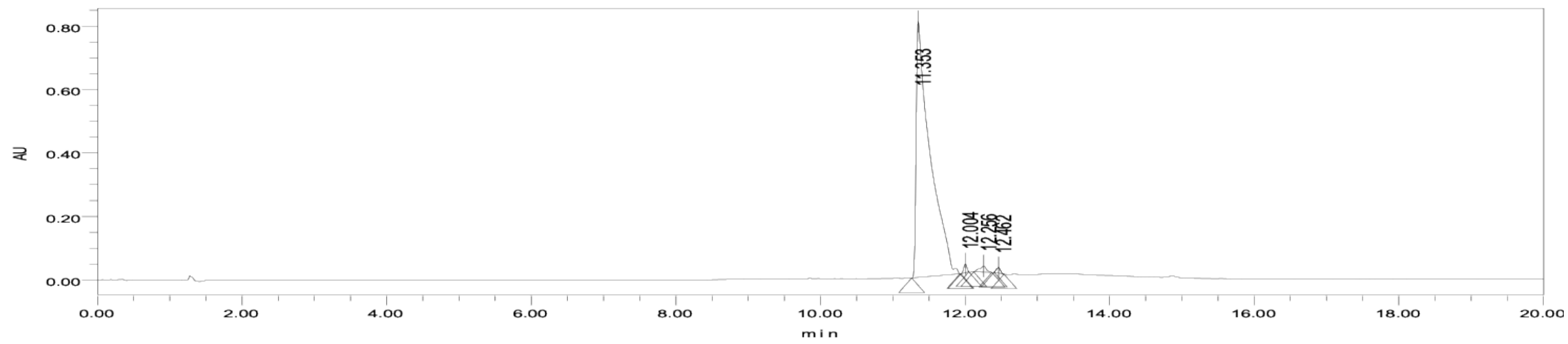

NO.	Rt (min)	Area (%)
1	11.353	97.48
2	12.004	0.76
3	12.256	1.16
4	12.462	0.61

Figure S30. HPLC chromatogram of **Au2**.

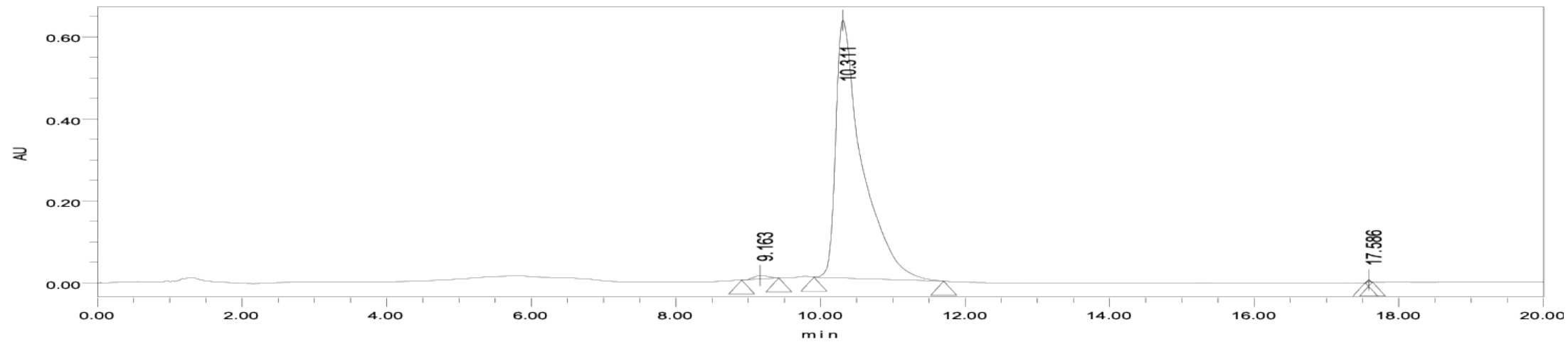

NO.	Rt (min)	Area (%)
1	9.163	0.73
2	10.311	99.17
3	17.586	0.10

Figure S31. HPLC chromatogram of **Au3**.

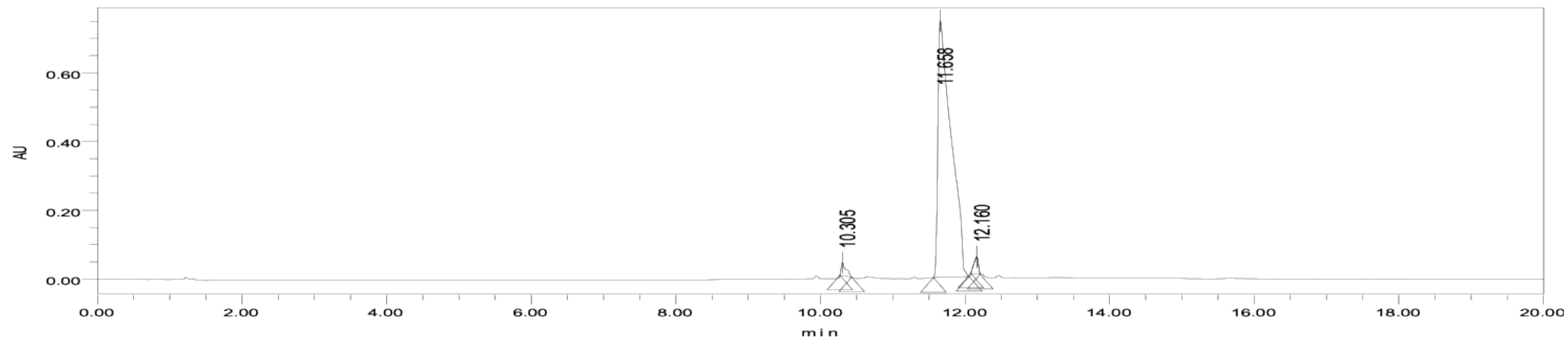

NO.	Rt (min)	Area (%)
1	10.305	1.87
2	11.658	96.00
3	12.160	2.13

Figure S32. HPLC chromatogram of **Au4**.

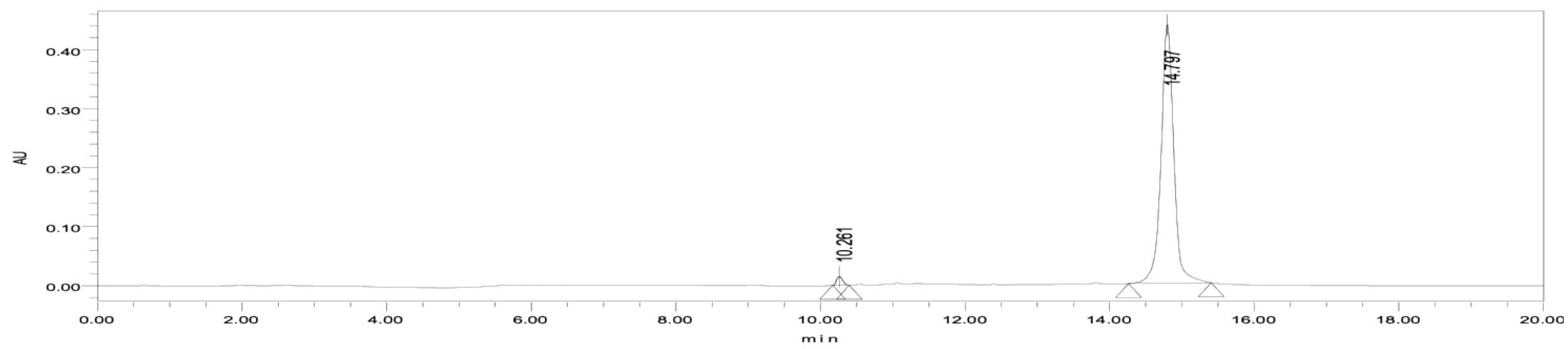

NO.	Rt (min)	Area (%)
1	10.261	1.59
2	14.797	98.41

Figure S33. HPLC chromatogram of **Au5**.

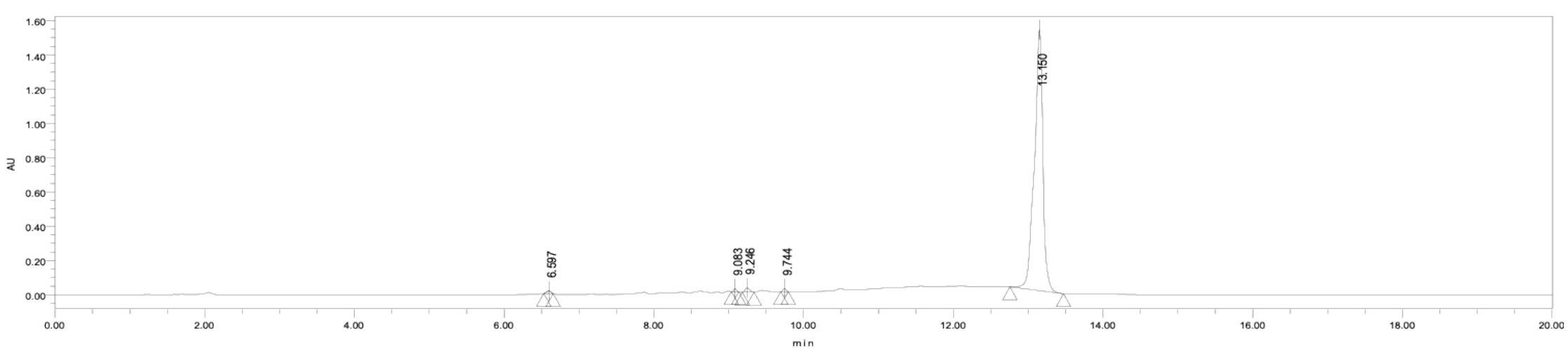

NO.	Rt (min)	Area (%)
1	6.597	0.49
2	9.083	0.36
3	9.246	0.83
4	9.744	0.44
5	13.150	97.88

Figure S34. HPLC chromatogram of **Au6**.

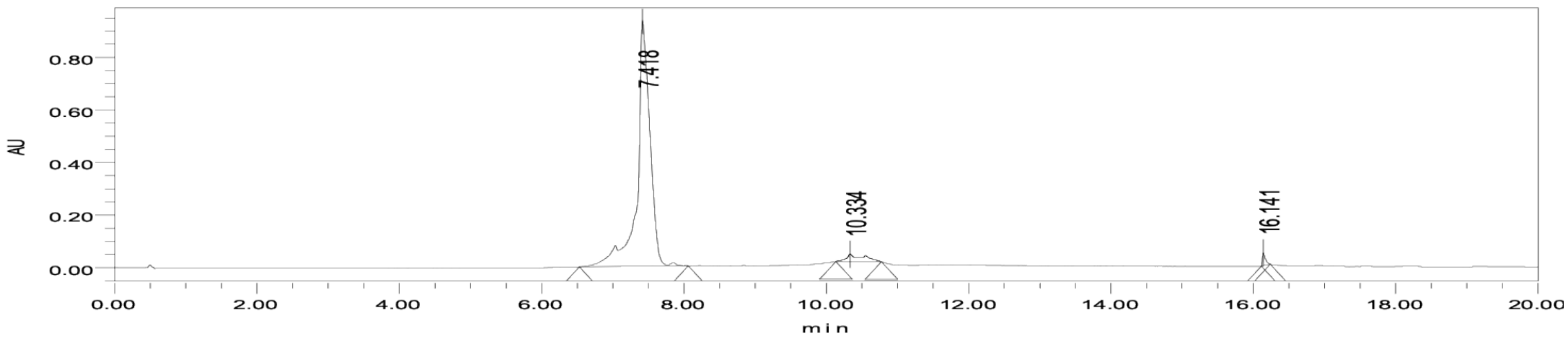

NO.	Rt (min)	Area (%)
1	7.418	95.10
2	10.334	3.72
3	16.141	1.18

Figure S35. HPLC chromatogram of **Au7**.

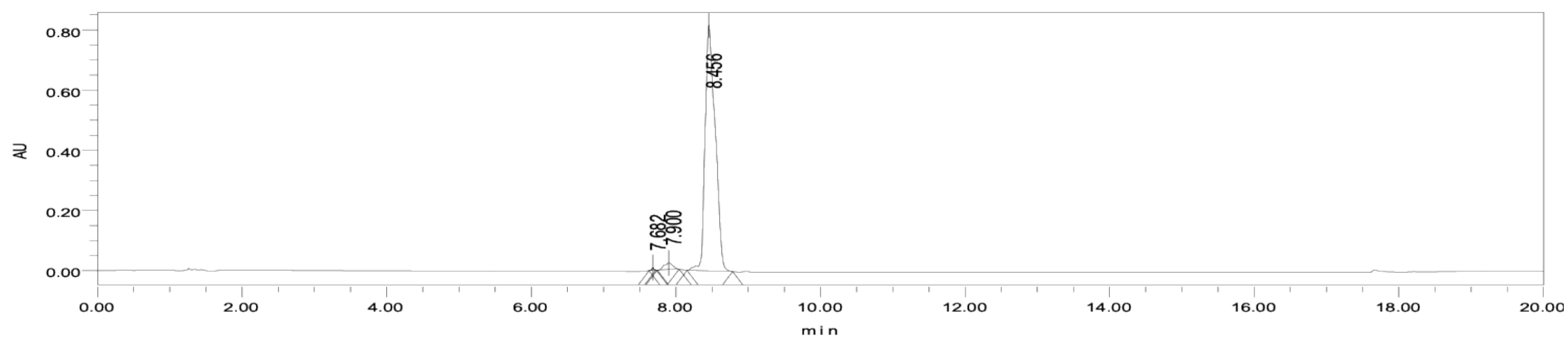

NO.	Rt (min)	Area (%)
1	7.682	0.19
2	7.900	2.26
3	8.456	97.55

Figure S36. HPLC chromatogram of **Au8**.

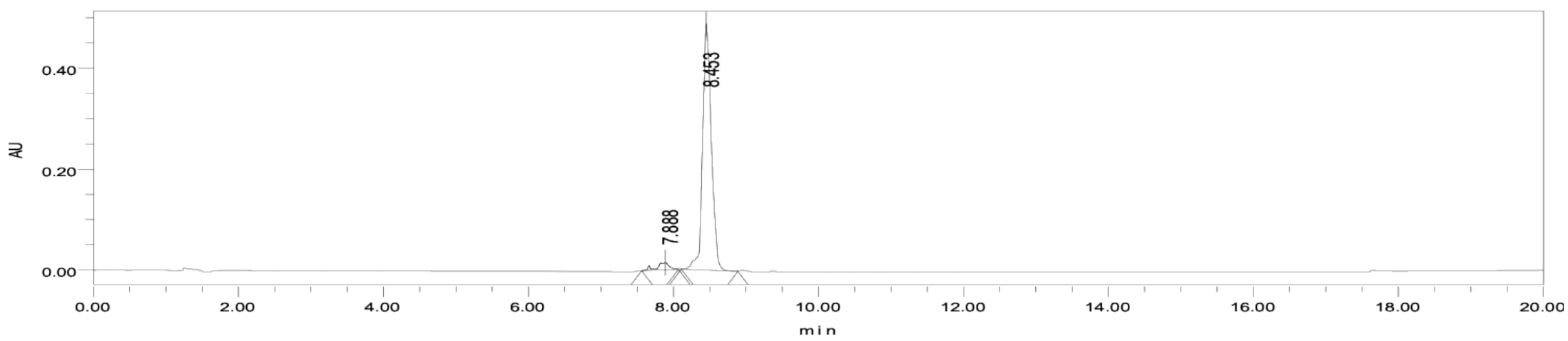

NO.	Rt (min)	Area (%)
1	7.888	3.77
2	8.453	96.23

Figure S37. HPLC chromatogram of **Au9**.

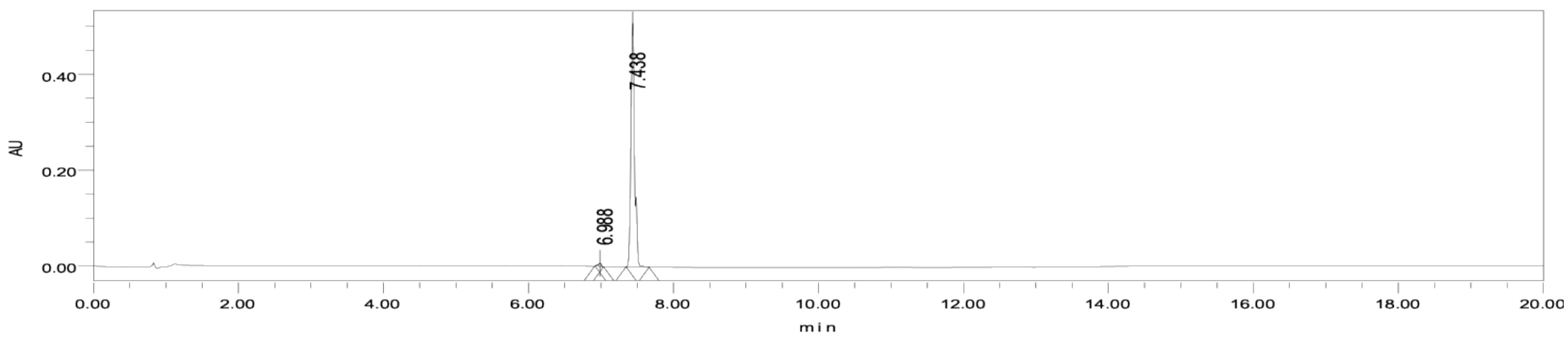

NO.	Rt (min)	Area (%)
1	6.988	1.17
2	7.438	98.83

Figure S38. HPLC chromatogram of **Au10**.

Stability Analysis

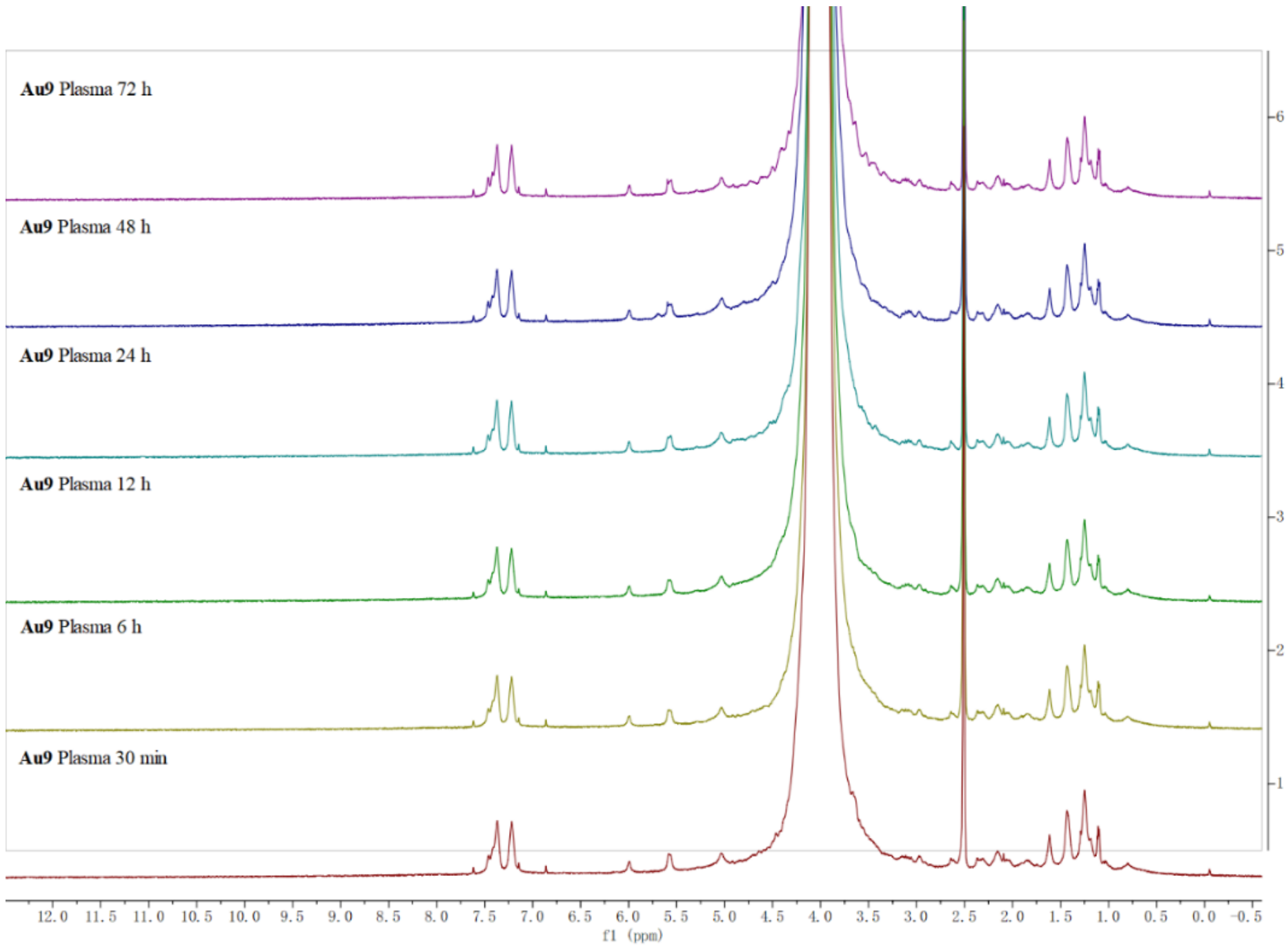

Figure S39. The ^{1}H NMR spectra of **Au9** (5.0 mM) in DMSO-d_6 / plasma **/** D_2O = 19 : 4 : 1 over 72 h at 298 K.

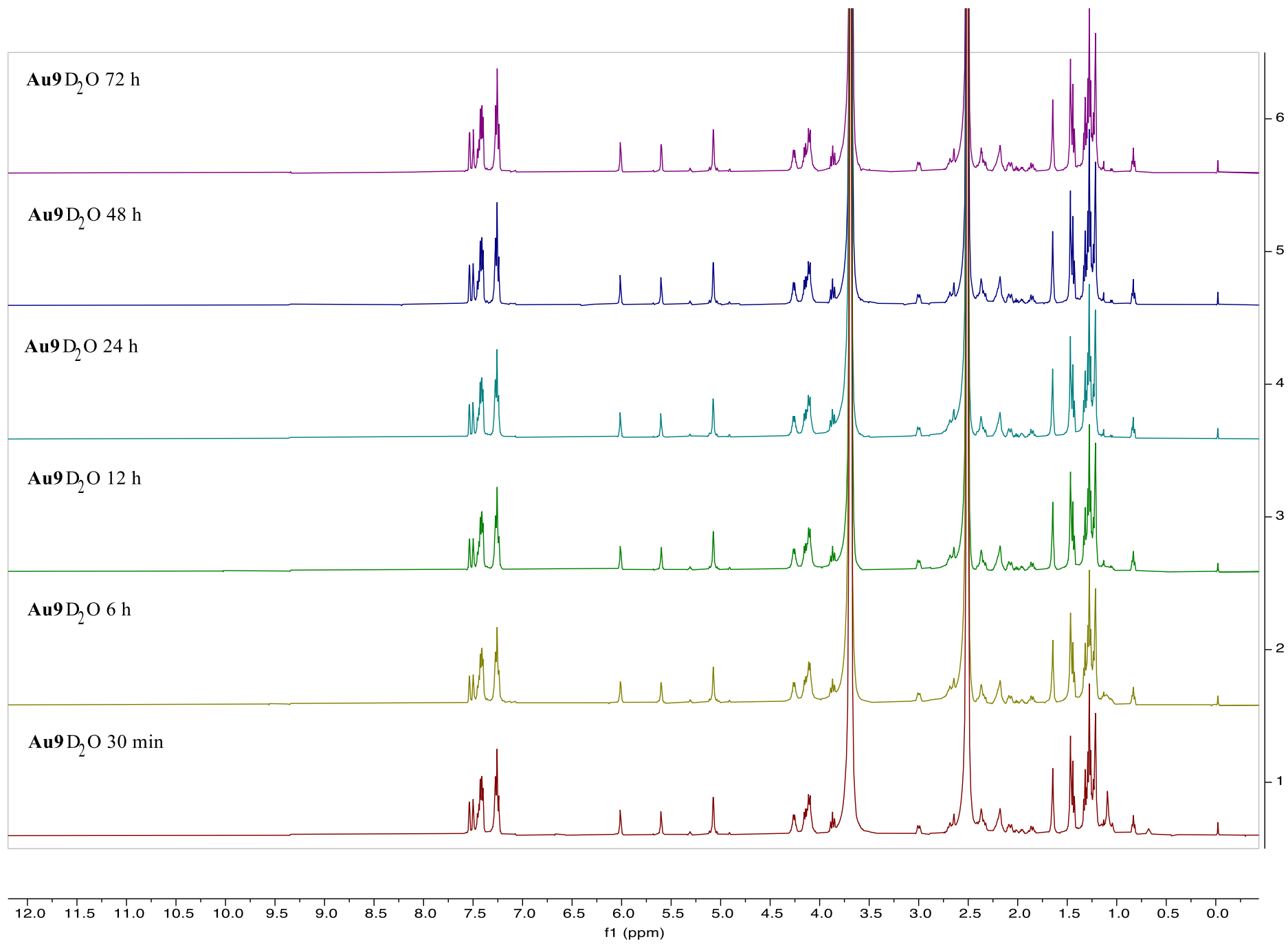

Figure S40. The ^{1}H NMR spectra of **Au9** (5.0 mM) in DMSO-d_6 / D_2O = 9 : 1 over 72 h at 298 K.

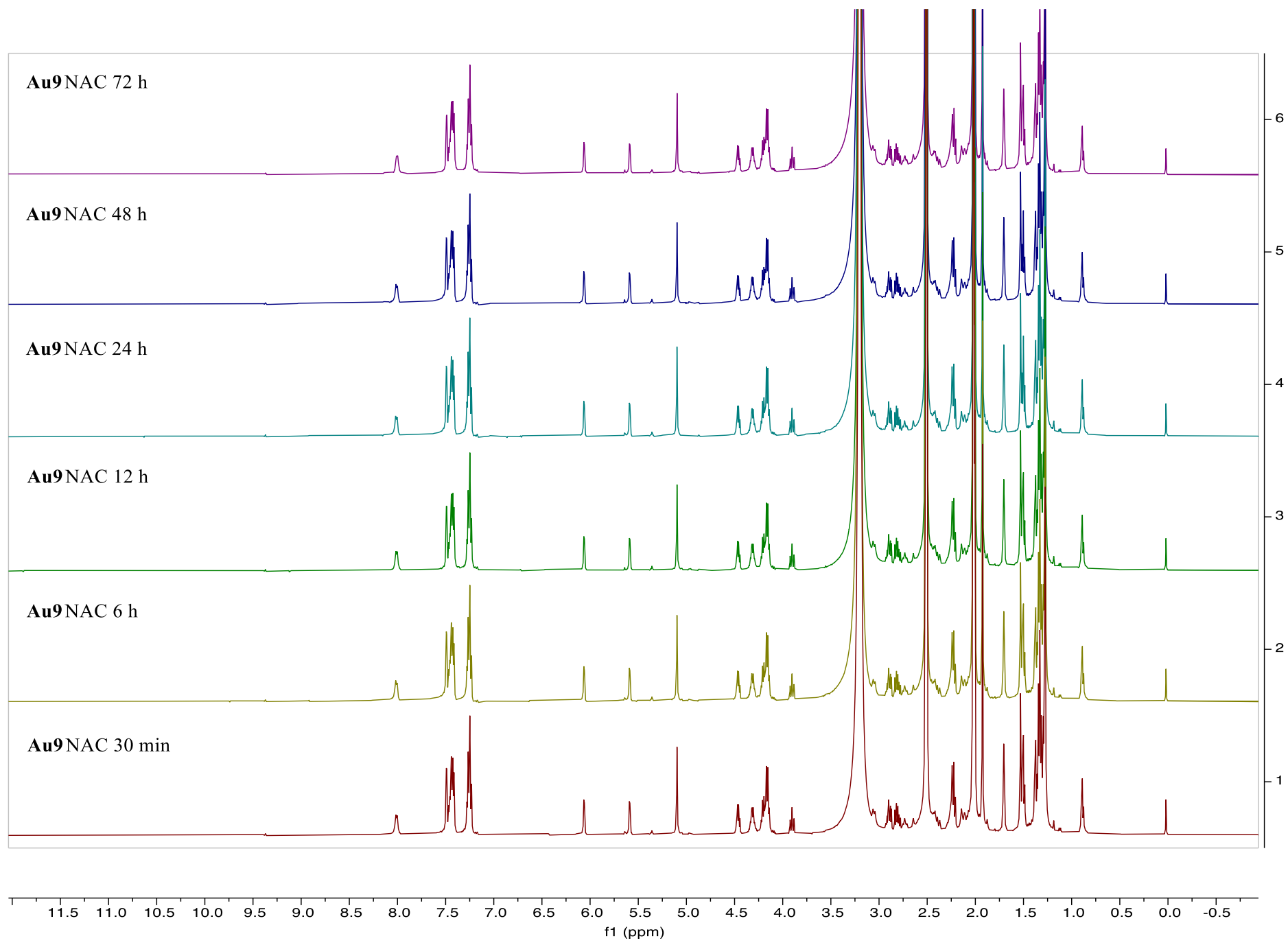

Figure S41. The ^{1}H NMR spectra of **Au9** (5.0 mM) and NAC (5.0 mM) in DMSO-d_6 / Acetonitrile-d_3 = 1 : 1 over 72 h at 298 K.

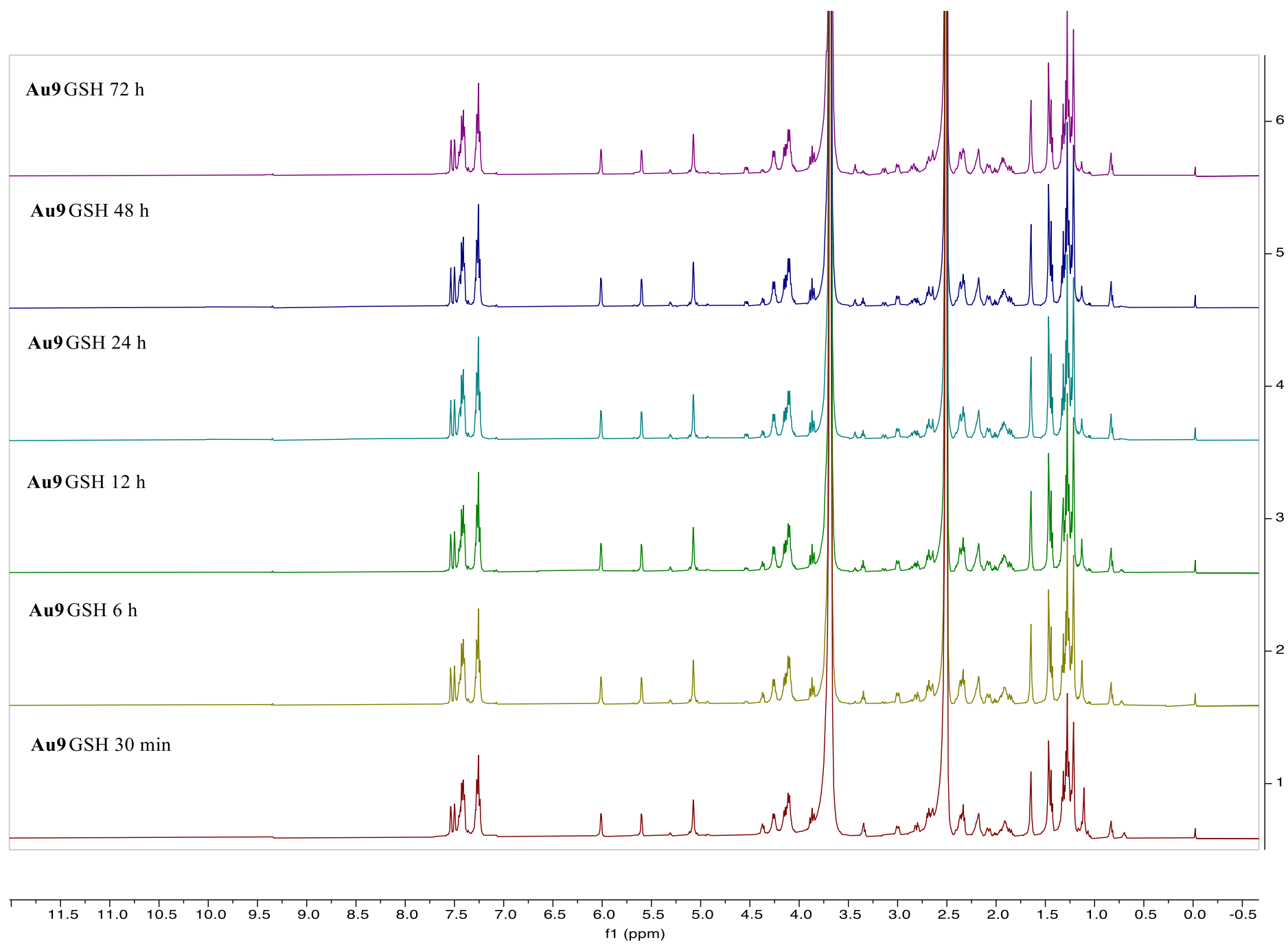

Figure S42. The ^{1}H NMR spectra of **Au9** (5.0 mM) and GSH (5.0 mM) in DMSO-d_6 / D_2O = 9 : 1 over 72 h at 298 K.

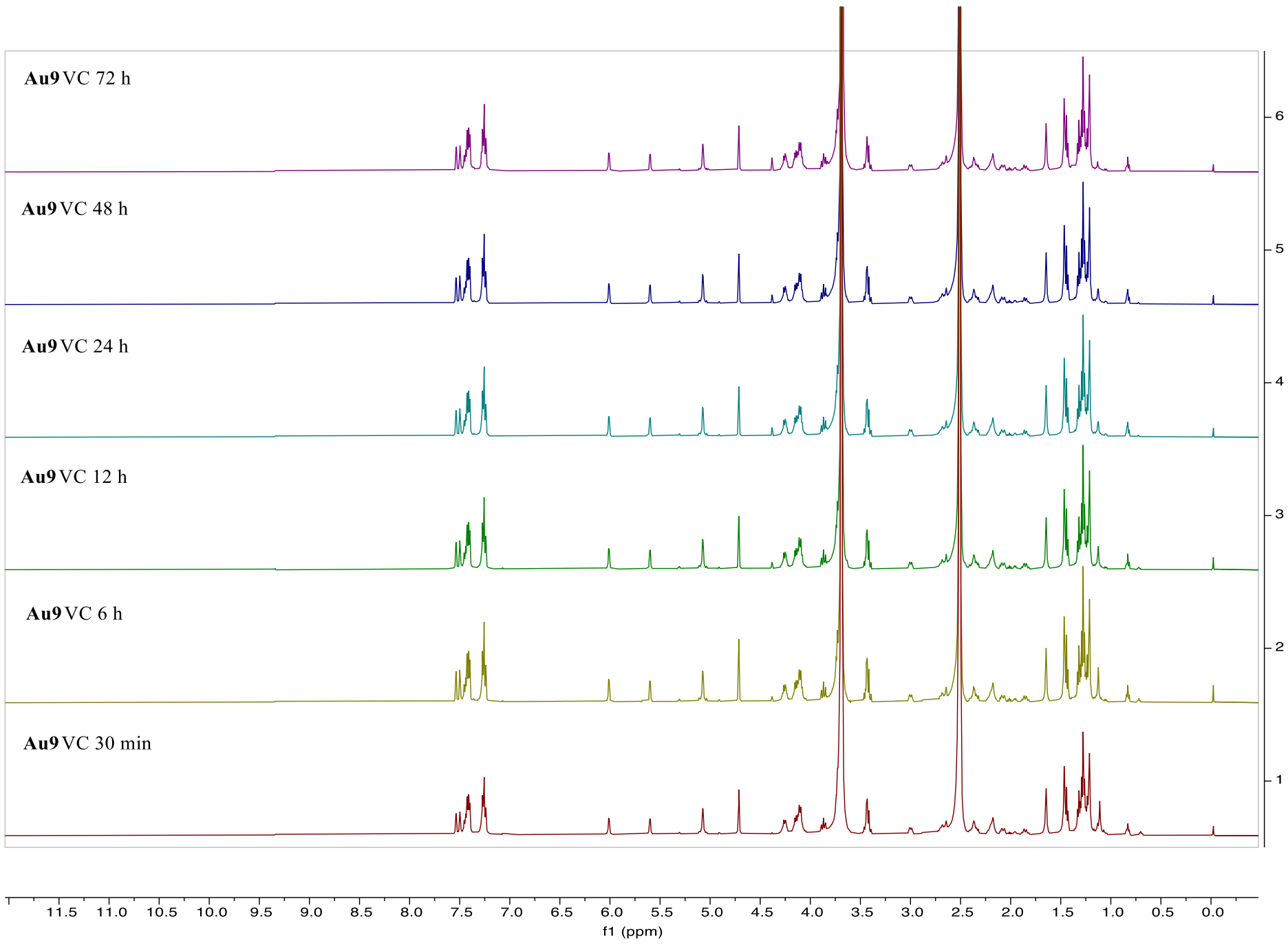

Figure S43. The ^{1}H NMR spectra of **Au9** (5.0 mM) and VC (5.0 mM) in DMSO-*d*$_6$ / D_2O = 9 : 1 over 72 h at 298 K.

Table S1. Antiproliferative activity of the complexes against three tumor cell lines after 72 h of treatment.

Complex	HepG2	Huh7	Hepa 1-6
**Au1**	4.14 ± 0.55	> 10	4.69 ± 0.87
**Au2**	> 10	> 10	> 10
**Au3**	7.87 ± 1.23	> 10	6.96 ± 0.90
**Au4**	0.51 ± 0.08	2.51 ± 2.41	0.32 ± 0.06
**Au5**	0.90 ± 0.07	1.75 ± 0.22	0.86 ± 0.12
**Au6**	1.89 ± 0.23	4.85 ± 0.89	1.28 ± 0.23
**Au7**	0.53 ± 0.06	0.43 ± 0.03	0.68 ± 0.23
**Au8**	0.48 ± 0.06	0.19 ± 0.01	0.56 ± 0.08
**Au9**	0.20 ± 0.02	0.23 ± 0.02	0.41 ± 0.05
**Micheliolide**	8.21 ± 1.12	9.29 ± 1.33	8.67 ± 1.22
**Auranofin**	2.66 ± 0.44	1.27 ± 0.19	0.75 ± 0.04
**Cisplatin**	1.02 ± 0.15	0.91 ± 0.13	1.39 ± 0.18
**Oxaliplatin**	1.18 ± 0.14	1.60 ± 0.25	3.75 ± 0.68

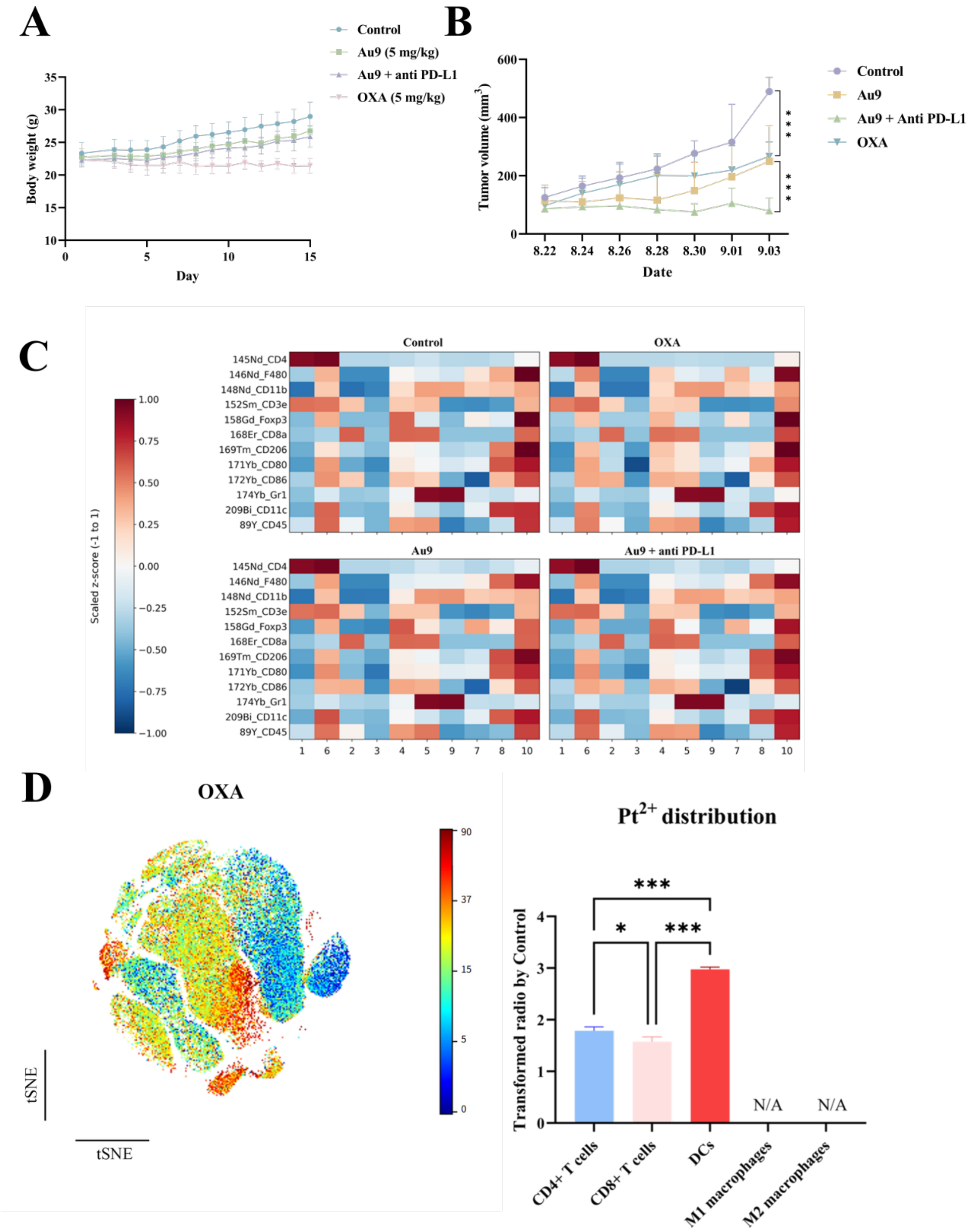

Figure S44. (A) Weight changes of mice after four groups of treatments. (B) The tumor volume was recorded. (C) Mass cytometry heatmap of CyTOF analysis. (D) Mass cytometry analysis of platinum ion distribution in the OXA administration group. Data for M1 and M2 macrophages were denoted as "N/A" because the Pt signals in these subsets were consistently below the lower limit of quantification (LLOQ). n = 3; *, $P < 0.05$; ***, $P < 0.001$ when compared with control group.

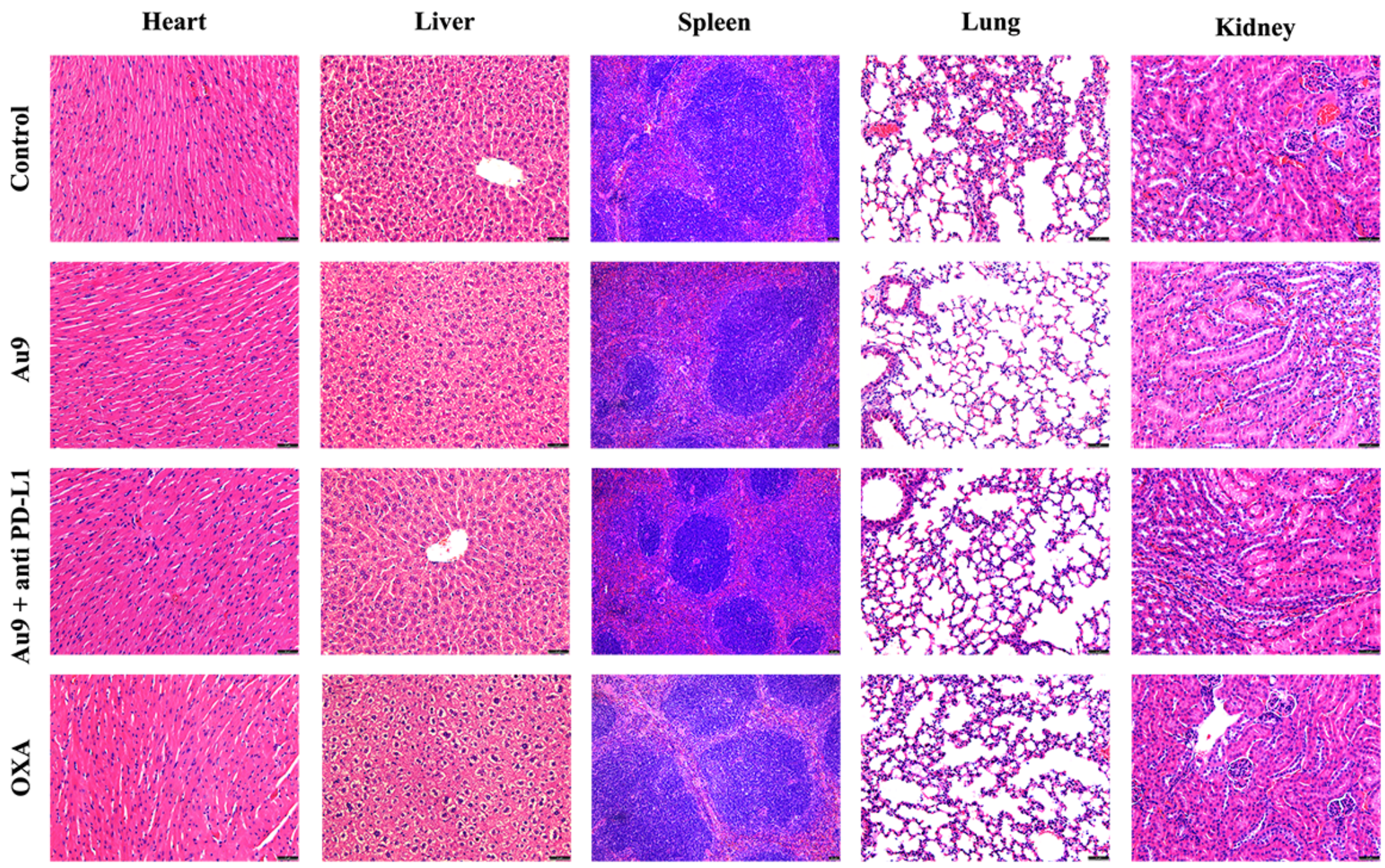

Figure S45. The five organs were analyzed after four treatment groups by H&E staining. Scale bar: 200 μm.

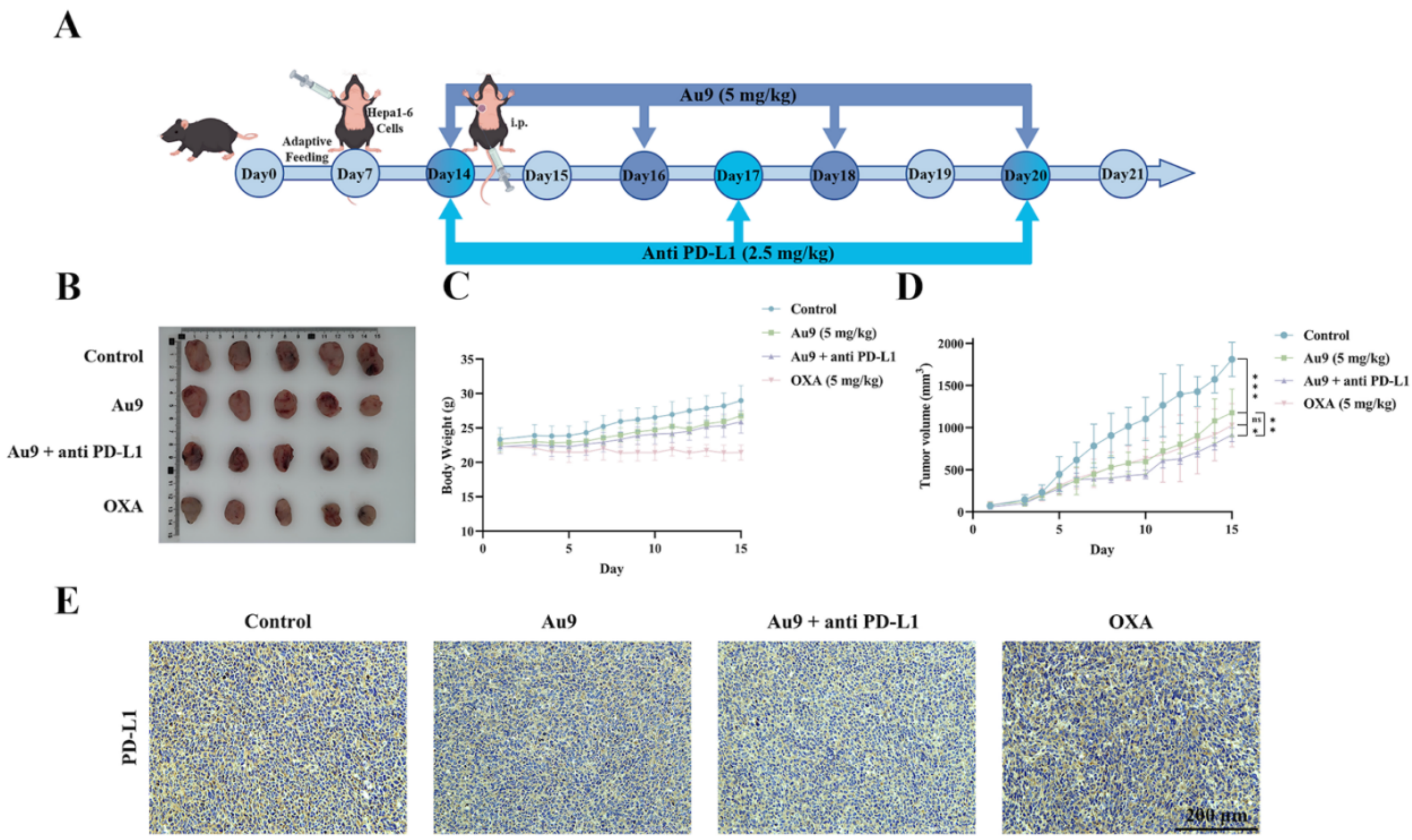

Figure S46. (A) Schematic illustrating the construction of a tumor-bearing model utilizing C57BL/6 mice. (B, D) Differences in tumor volume among mice in different treatment groups. n = 5. (C) Changes in body weight of mice during treatment across different treatment groups. n = 5. (E) The PD-L1 protein expression in tumor tissues was analyzed by Immunohistochemistry. Scale bar: 200 μm. n = 3; ns, p > 0.05; *, P < 0.05; **, P < 0.01; ***, P < 0.001. Compared with the control group.

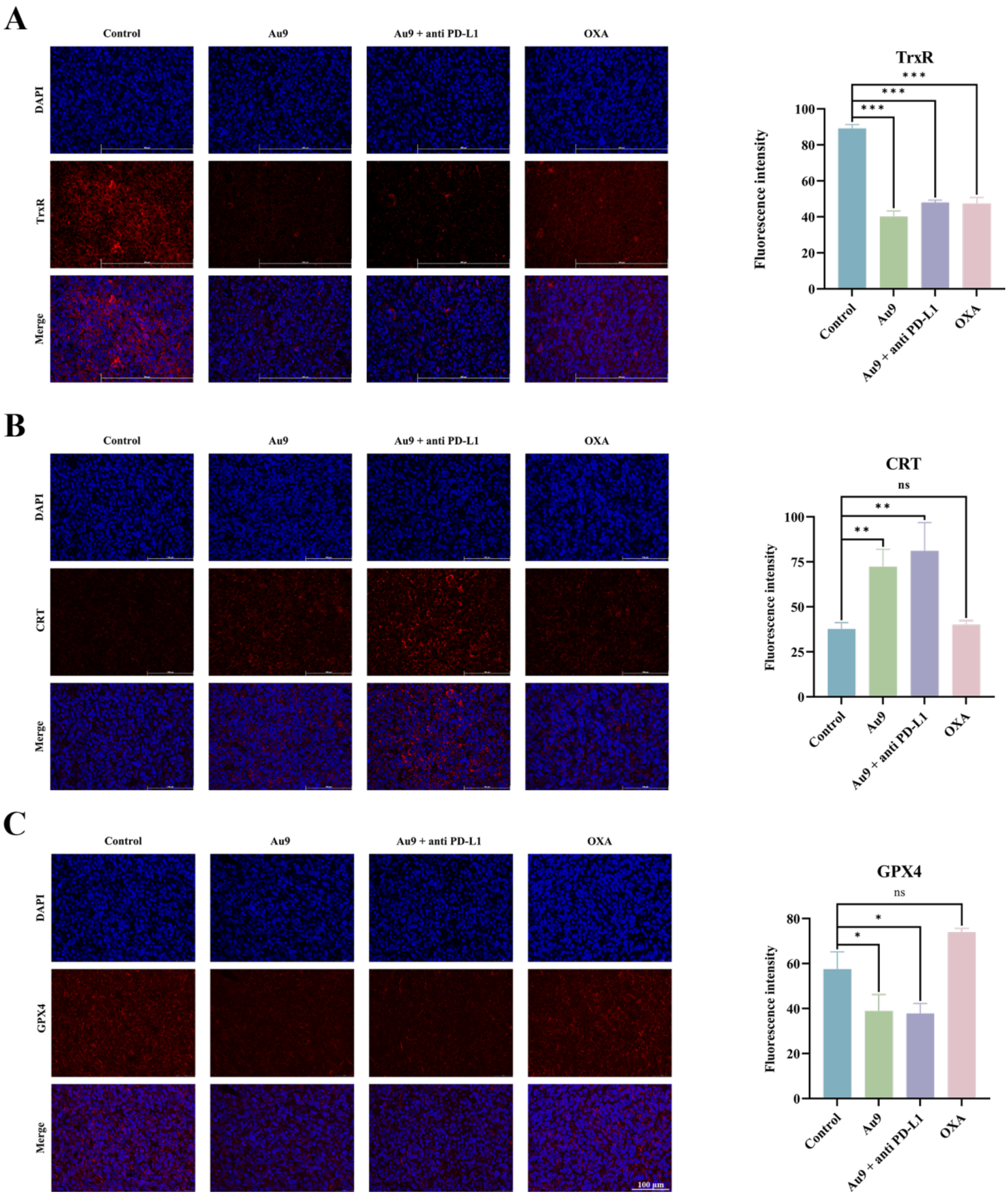

Figure S47. (A-C) Immunofluorescence staining analysis of TrxR/CRT/GPX4 protein expression within tumors across different treatment groups. n = 3; ns, P > 0.05; *, P < 0.05; **, P < 0.01; ***, P < 0.001 when compared with control group. Scale bar: 100 μm.

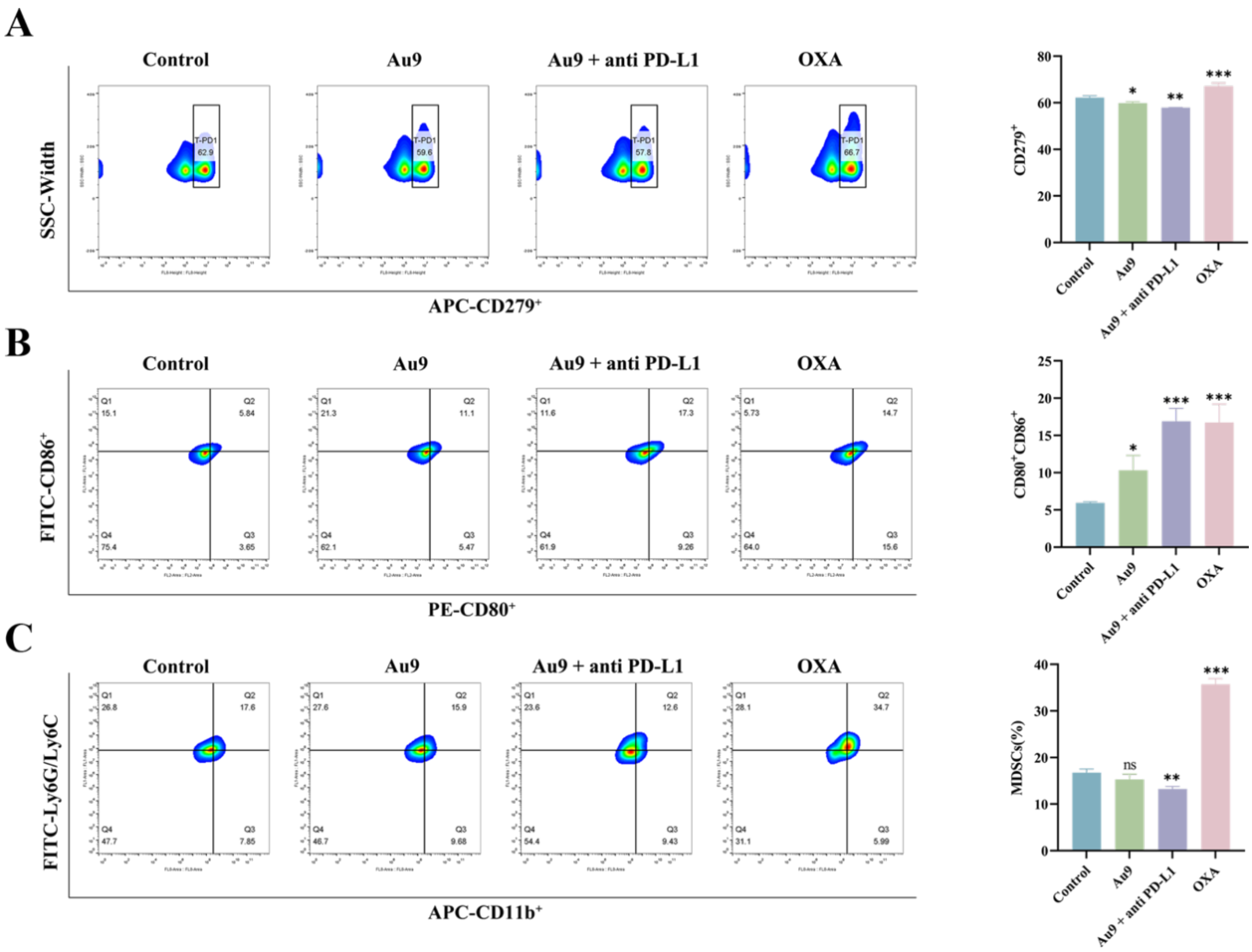

Figure S48. (A) Flow cytometry analysis of intratumoral PD-1 ($CD279^+$) expression among different treatment groups. (B) and (C) Flow cytometric analysis of intratumoral mature DCs and MDSCs in different treatment groups. n = 3; ns, P > 0.05; *, P < 0.05; **, P < 0.01; ***, P < 0.001 when compared with control group.

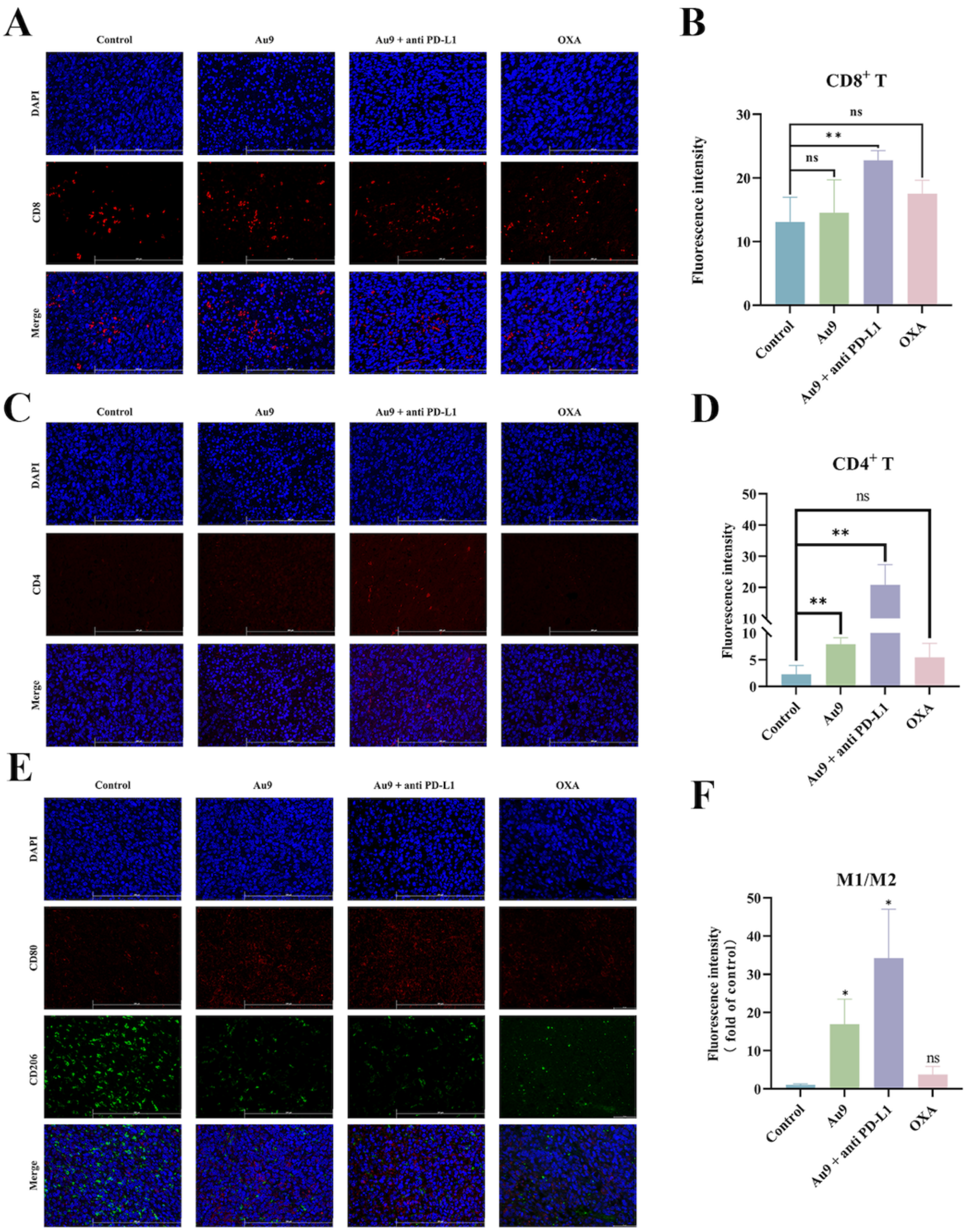

Figure S49. (A-F) Immunofluorescence staining analysis of immunomarker expression within tumors across different treatment groups. n = 3; ns, P > 0.05; *, P < 0.05; **, P < 0.01 when compared with control group. Scale bar: 200 μm.

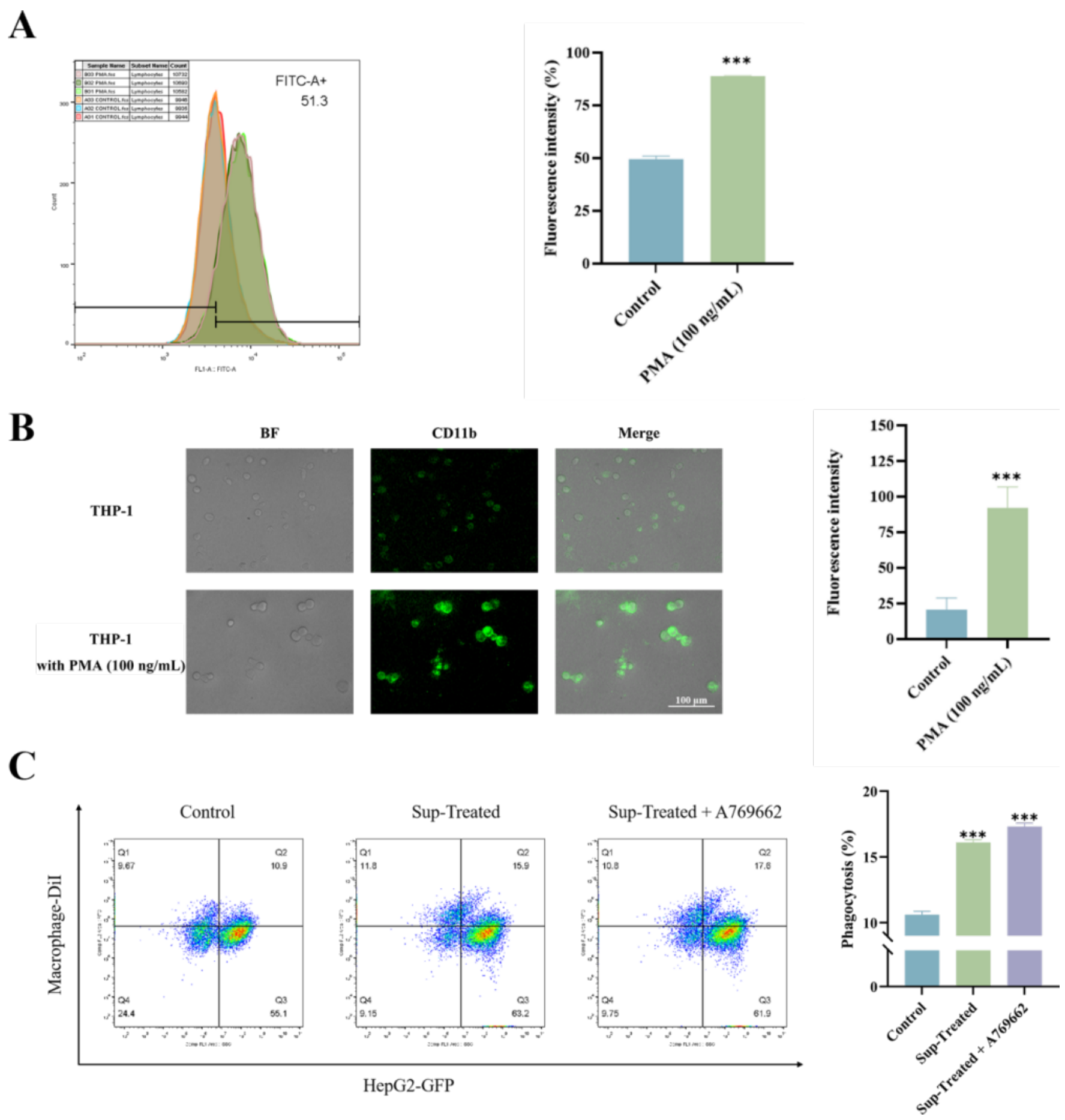

Figure S50. (A) and (B) Flow cytometry and confocal fluorescence analysis of PMA-induced differentiation of THP-1 cells into macrophages. Scale bar: 100 μm. (C) Flow cytometry analysis of macrophage co-culture systems. n = 3; ***, P < 0.001 when compared with control group.

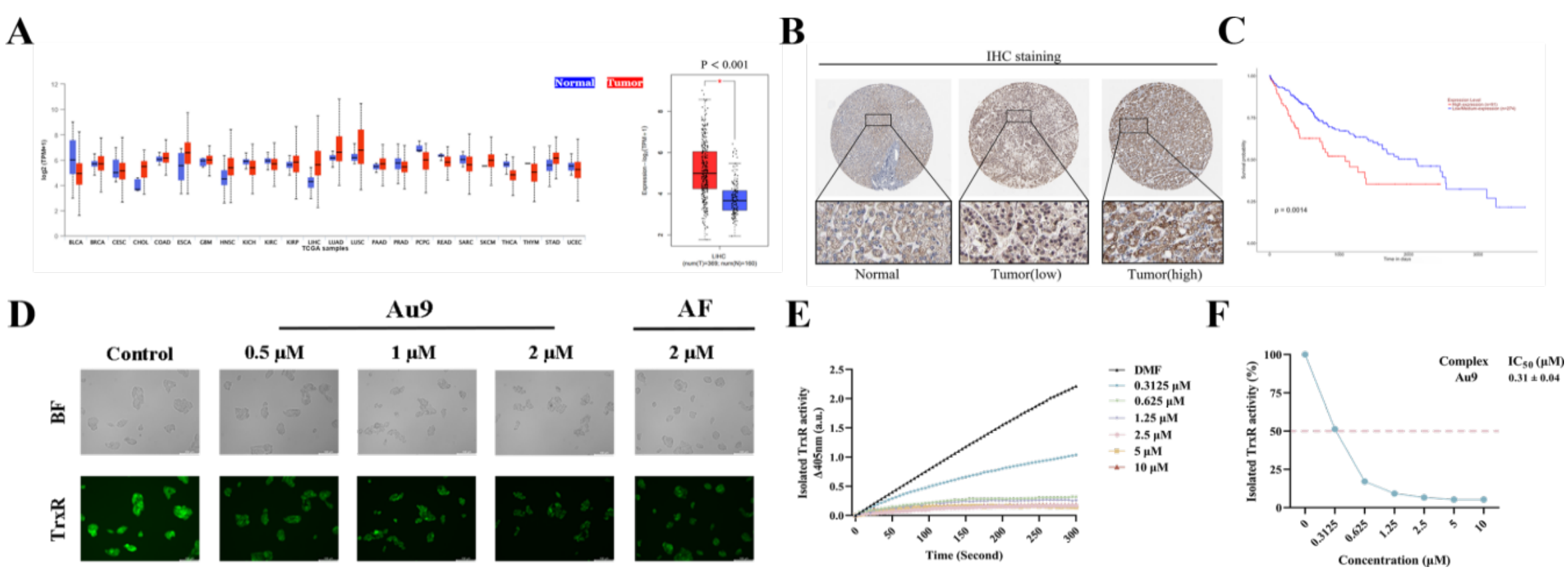

Figure S51. (A) Differential expression of the TXNRD1 (gene name of TrxR) across various cancers. (B) Immunohistochemical staining of TXNRD1 protein in different liver tissues. (C) Kaplan-Meier survival curve showing a significant association between TXNRD gene overexpression and patient survival rate (p = 0.0014). (D) Intracellular TrxR activity was analyzed using the TrxR-green probe. Scale bar: 100 μm. (E) Effect of various concentrations of **Au9** on the isolated TrxR activity. (F) Half-maximal inhibitory concentration (IC_{50}) of **Au9** against isolated TrxR.

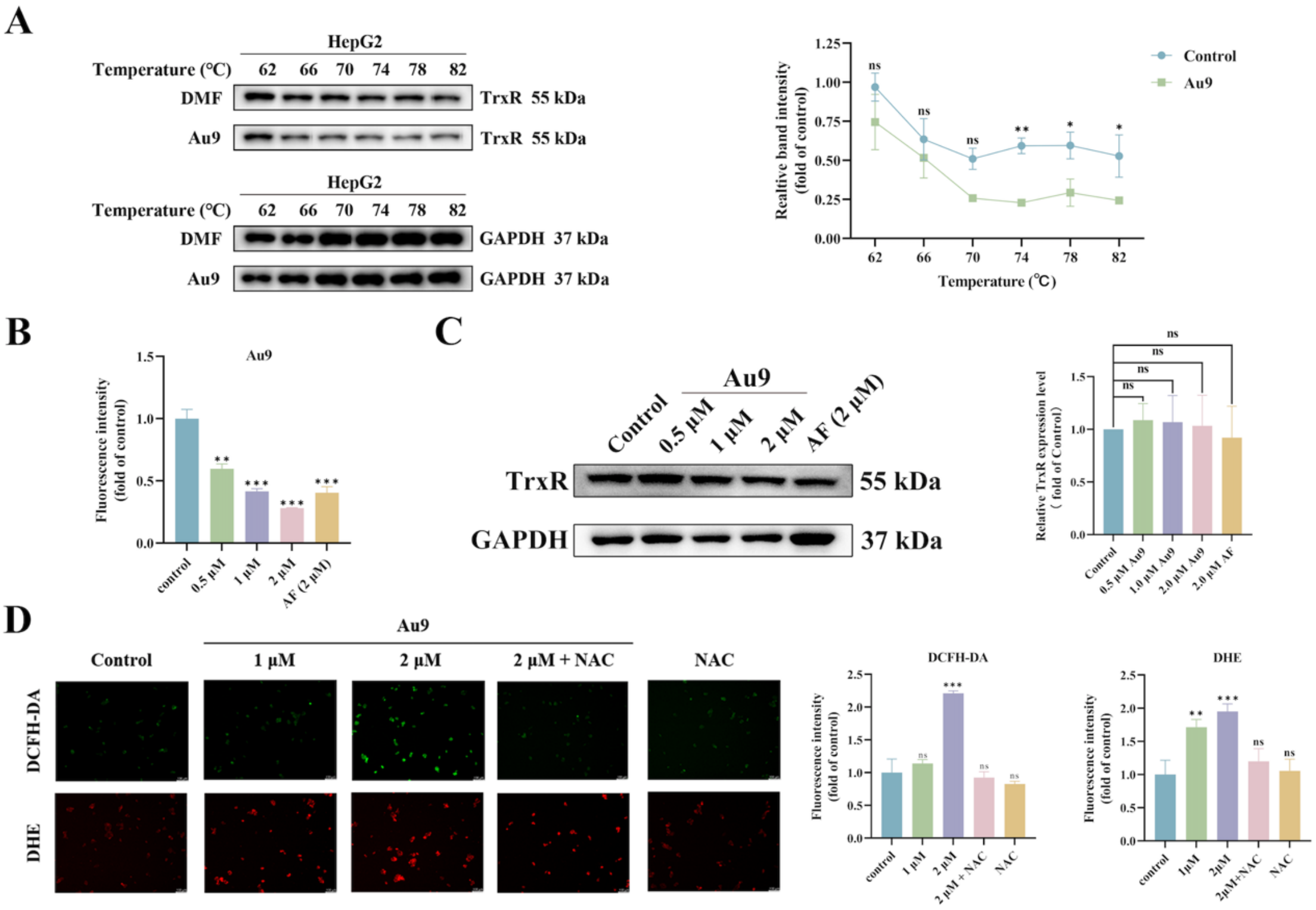

Figure S52. (A) The TrxR protein was analyzed by CETSA. (B) Fluorescence quantification of TrxR activity. (C) The TrxR protein expression levels were analyzed by the western blot assays. (D) DCFH-DA and DHE probes were used to detect intracellular ROS and superoxide anion levels. Scale bar: 100 μm. n = 3; ns, P > 0.05; *, P < 0.05; **, P < 0.01; ***, P < 0.001 when compared with control group.

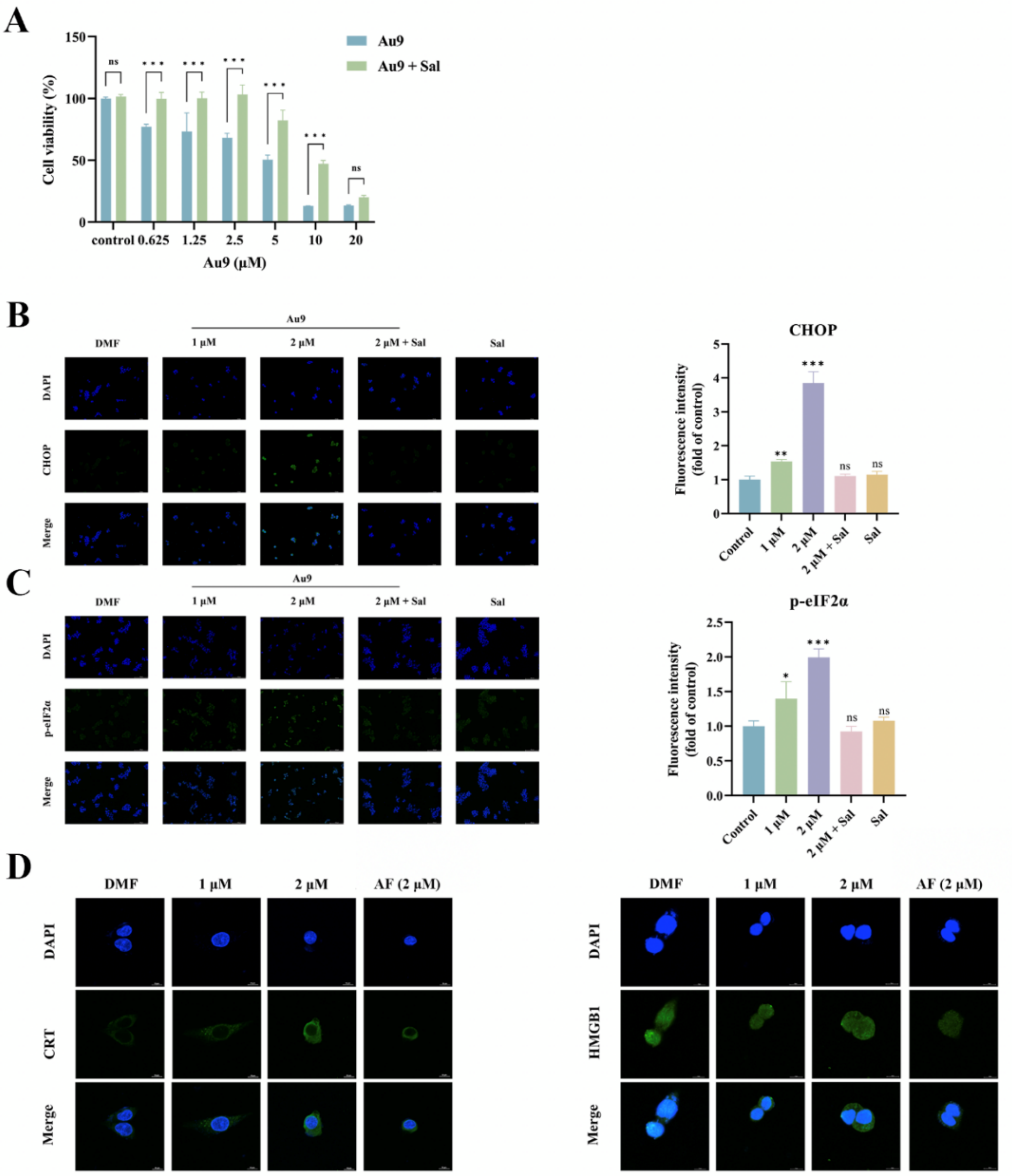

Figure S53. (A) Cell viability following **Au9** treatment with or without salubrinal. (B) and (C) Immunofluorescence analysis of ERS-related markers CHOP and p-eIF2α following different treatments, along with reversal experiments. Scale bar: 100 μm. (D) Immunofluorescence of CRT and HMGB1 as indicators of ICD in HepG2 cells treated with different concentrations of complexes. n = 3; ns, $P > 0.05$; *, $P < 0.05$; **, $P < 0.01$; ***, $P < 0.001$ when compared with control group. Scale bar: 10 μm.

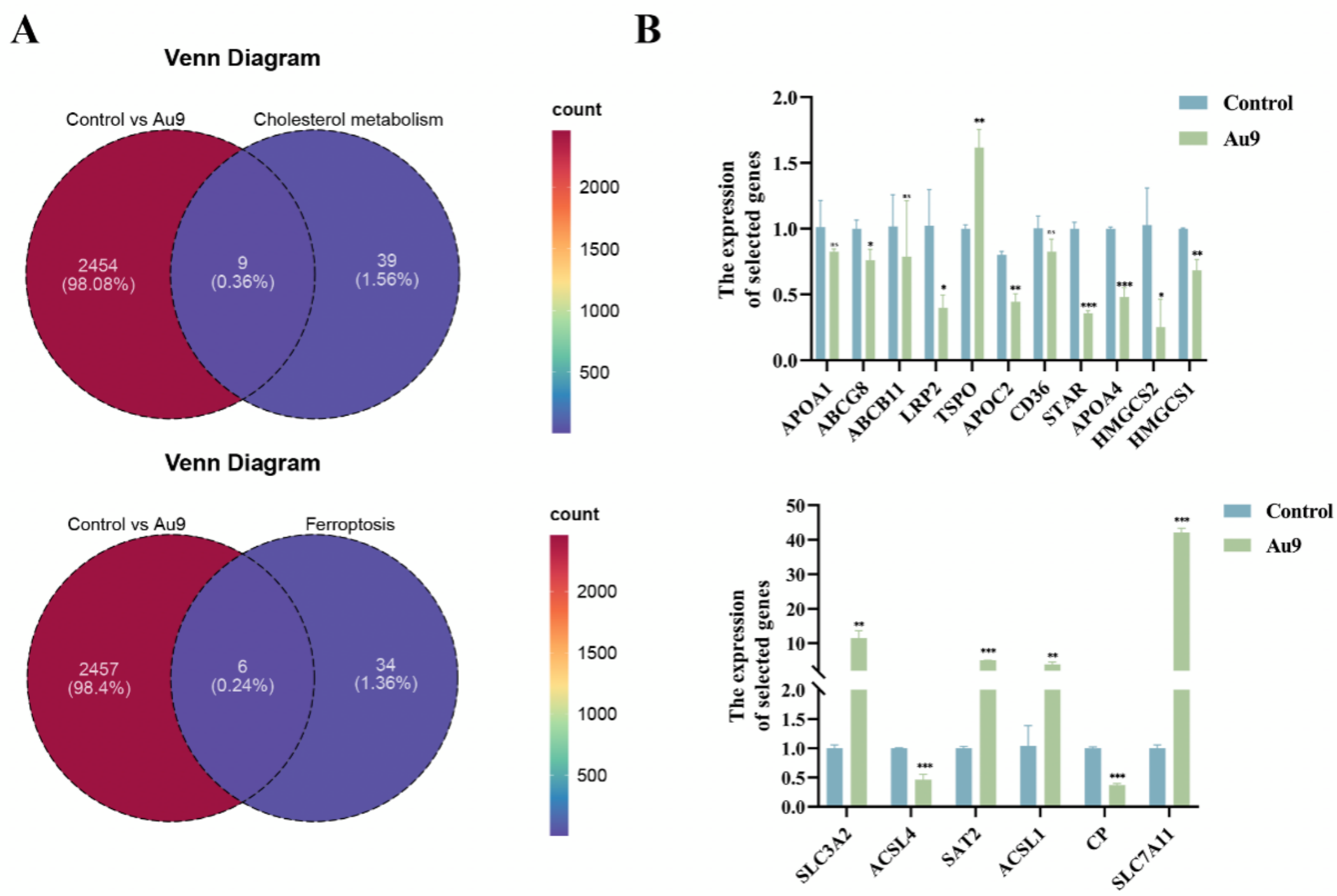

Figure S54. (A) Transcriptome Venn diagram of ferroptosis and cholesterol pathways. (B) qPCR analysis of selected genes in HepG2 cells after **Au9** treatment. n = 3; ns, P > 0.05; *, P < 0.05; **, P < 0.01; ***, P < 0.001 when compared with control group.

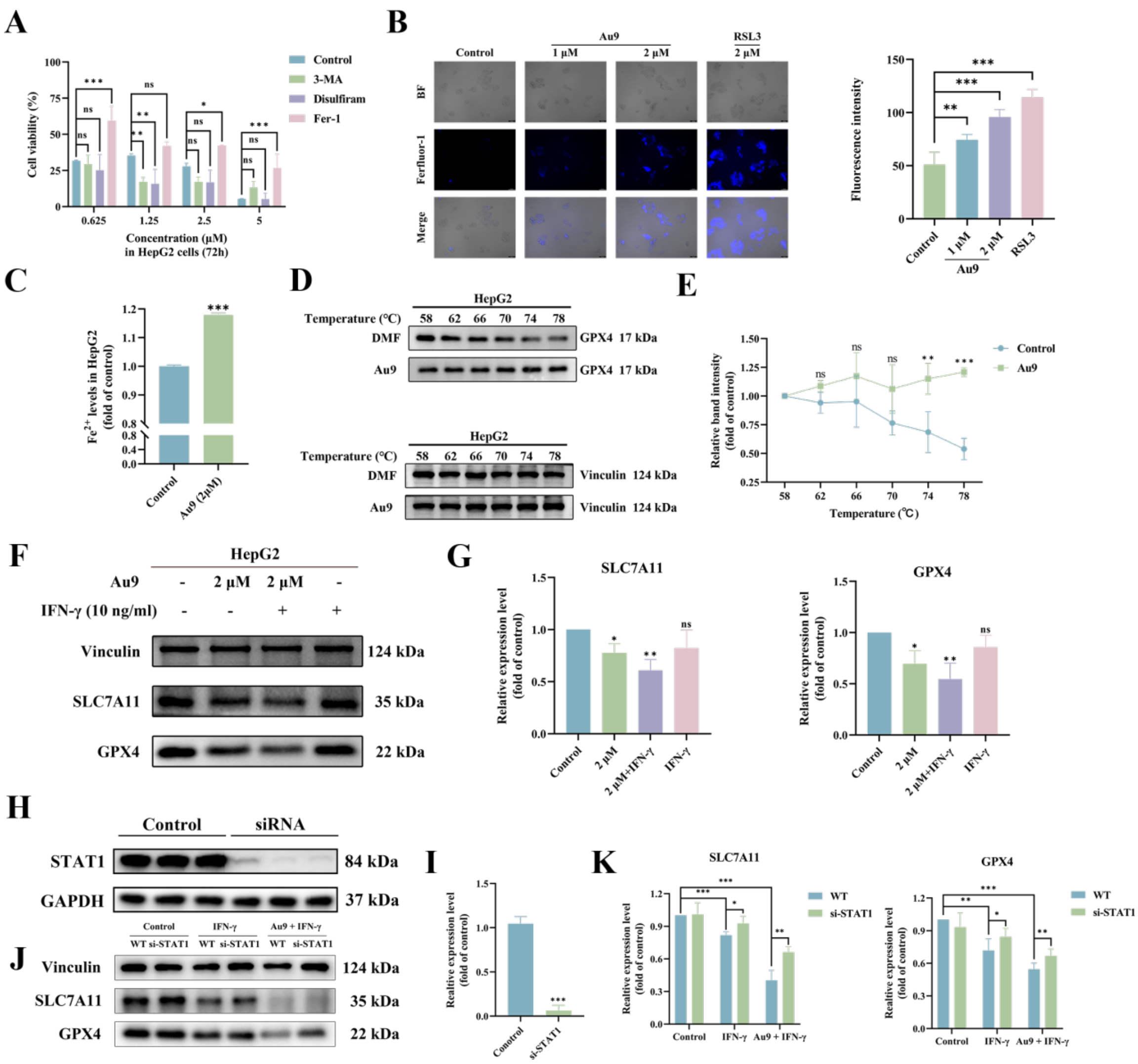

Figure S55. (A) Three inhibitors were employed to analyze cell viability after exposure to **Au9**. (B) Representative Ferfluor-1 fluorescence images of HepG2 cells following the indicated treatments. Scale bar: 50 μm. (C) Analysis of intracellular ferrous ions. (D) and (E) CETSA was employed to analyze GPX4 protein. (F) and (G) The expression of SLC7A11 and GPX4 was analyzed by western blot assays. (H) and (I) Expression of STAT1 protein in HepG2 cells following siRNA-mediated STAT1 gene silencing. (J) and (K) Following STAT1 gene knockdown in HepG2 cells, treatment with **Au9** and IFN-γ for 24 h was performed to assess ferroptosis-related markers SLC7A11 and GPX4. n = 3; ns, P > 0.05; *, P < 0.05; **, P < 0.01; ***, P < 0.001 when compared with control group.

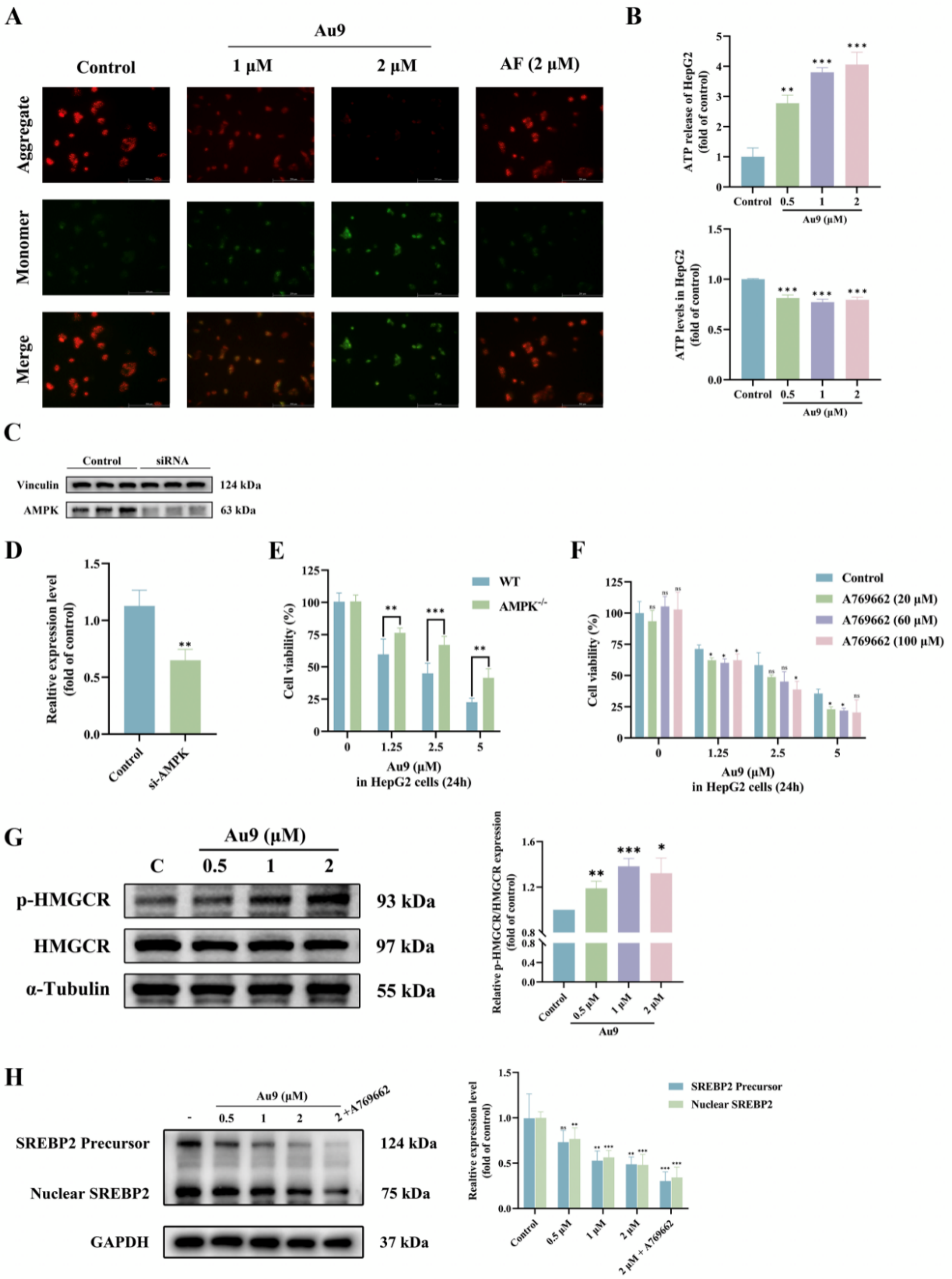

Figure S56. (A) and (B) The mitochondrial membrane potential and ATP levels in HepG2 cells were analyzed after **Au9** treatment. Scale bar: 200 μm. (C) and (D) The validation of siRNA-mediated silencing of AMPK. (E) Cell viability of wild-type (WT) and si-AMPK HepG2 cells treated with **Au9** for 24 h. (F) Cell viability of HepG2 cells treated with **Au9** in the presence or absence of the AMPK activator A769662 (20, 60, and 100 μM) for 24 h. (G) The p-HMGCR/HMGCR in HepG2 cells was analyzed by the western blot assays. (H) The changes

of SREBP2 precursor and nuclear SREBP2 in HepG2 cells treated with **Au9** alone or in combination with A769662. n = 3; ns, P > 0.05; *, P < 0.05; **, P < 0.01; ***, P < 0.001 when compared with control group.

References

[1] Zhai JD, Li D, Long J, et al. Biomimetic semisynthesis of arglabin from parthenolide. *J Org Chem.* **2012**; 77(16): 7103-7107.

[2] Liu W, Bensdorf K, Proetto M, et al. NHC gold halide complexes derived from 4,5-diarylimidazoles: synthesis, structural analysis, and pharmacological investigations as potential antitumor agents. *J Med Chem*. **2011**; 54(24): 8605-8615.

[3] Zhang J, Zou H, Lei J, et al. Multifunctional Au^{I}-based AIEgens: manipulating molecular structures and boosting specific cancer cell imaging and theranostics. *Angew Chem Int Ed Engl.* **2020**; 59(18): 7097-7105.
